# Macrocycles of Saxitoxin: Insights into the Structure of Zetekitoxin AB

**DOI:** 10.1002/cbic.202500170

**Published:** 2025-04-04

**Authors:** Wenyuan Li, Srinivas R. Paladugu, Jordan P. Liles, Manju Karthikeyan, Kevin Chase, Shrinivasan Raghuraman, Matthew S. Sigman, Ryan E. Looper

**Affiliations:** Department of Chemistry, University of Utah, Salt Lake City, UT 84102, USA; Department of Chemistry, University of Utah, Salt Lake City, UT 84102, USA; Department of Chemistry, University of Utah, Salt Lake City, UT 84102, USA; School of Biological Sciences, University of Utah, Salt Lake City, UT 84102, USA; School of Biological Sciences, University of Utah, Salt Lake City, UT 84102, USA; School of Biological Sciences, University of Utah, Salt Lake City, UT 84102, USA; Department of Chemistry, University of Utah, Salt Lake City, UT 84102, USA; Department of Chemistry, University of Utah, Salt Lake City, UT 84102, USA

**Keywords:** guanidine, inhibitors, ion channels, natural products, toxins

## Abstract

Zeteketoxin AB is the only macrocyclic member of the bis-guanidinium ion toxins, and the only member reported to be more potent than the parent (+)-saxitoxin. A rationale for this exquisite potency remains difficult to develop due to the scarcity of natural material and a lack of consensus around the specific structure of the toxin itself. A strategy is reported, leveraging an intramolecular Michael addition to forge macrocycles bridging the saxitoxin core, mimicking the proposed structure of zetekitoxin AB. Intriguingly, these analogs do not form a hydrate at C12. Experimental and computational studies suggest that a macrocyclic framework destabilizes the hydrate, casting doubt on the presence of a macrocycle in zetekitoxin. Preliminary activity screening utilizing calcium imaging-based constellation pharmacology demonstrates several analogs to have potent pharmacological activity similar to (+)-saxitoxin despite the lack of the C12 hydrated ketone.

## 1. Introduction

**Figure 1.**
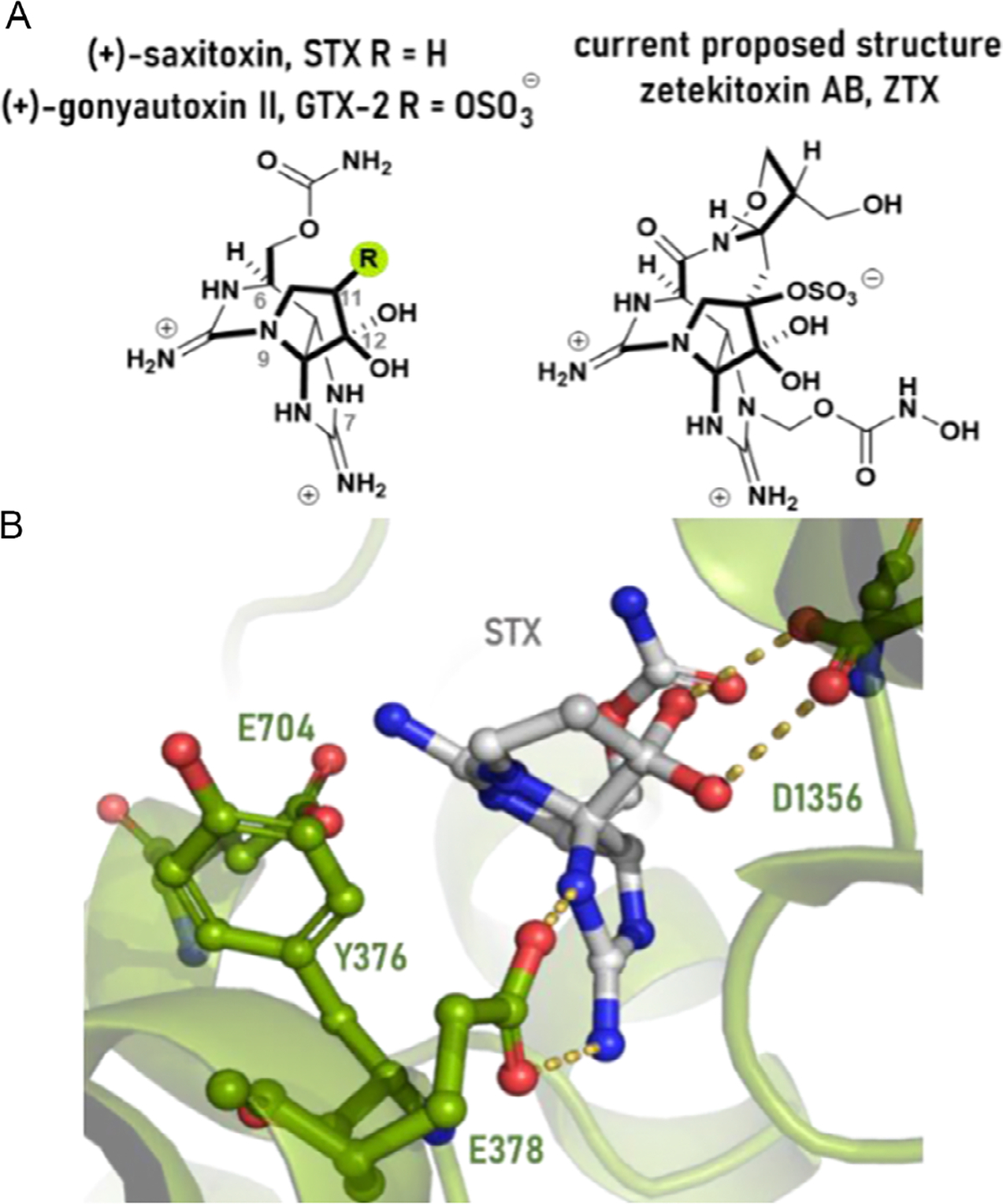
A) Structure of STX and proposed structures of ZTX. B) Structure of (+)-STX bound to the VGSC (PDB ID:6A91).

In 1969, Mosher and co-workers were investigating the toxic components of the Panamanian golden frog *Atelopus zeteki*. The primary toxin, referred to as atelopidtoxin A, was exceptionally toxic in mice (LD_50_ = 16 μg kg^−1^). The toxin demonstrated pharmacology similar to saxitoxin ((+)-STX) and tetrodotoxin but displayed 60–600× higher potency against voltage-gated sodium ion channel (VGSC) isoforms, compared to (+)-STX.^[1]^ That same year, *A. zeteki* was declared critically endangered, halting research on zeteketoxin AB. The scarcity of toxin quantities and limitations in structure elucidation techniques has posed challenges to determine the chemical structure of zeteketoxin AB. In the late 1990 s, Yotsu-Yamashita recovered a preserved 0.3 mg sample of the toxin and renamed the principal toxin zetekitoxin AB, honoring an intervening reclassification of the frog’s taxonomy. Bringing modern analytical techniques to bear on the structural assignment, they ultimately arrived at the structure for ZTX shown in Figure 1. While similar to (+)-STX, it is differentiated by its macrocyclic structure comprising an isoxazolidine bridging C6 and C11, the unique hydroxy carbamate functional group at N7, and a sulfate ester at C11 (which can be found in (+)-STX relatives like gonyautoxin-II (+)-GTX2). Even with modern analytical data, the structural assignment of this toxin was not straightforward and several alternative structures have been proposed that do not incorporate the C6–C11 macrocyclic bridge.^[2,3]^

Our understanding of how these guanidinium ion toxins interact with VGSCs is evolving and has recently been advanced with recent structures of the VGSC-STX complex (Figure 1B).^[4,5]^ This structure supported synthesis-guided structure–function relationships that require the C12 hydrate → D1356 and N7 → E378 interactions to maintain significant potency.^[6,7]^ Since N7 is substituted in ZTX, it remains unclear why the ZTX scaffold would retain such significant potency. The presence of the proposed macrocycle in ZTX can potentially impart conformational restrictions and increase molecular volume, increasing the strength of electrostatic interactions or the off-rate of the toxin, both of which could lead to an increase in binding affinity. Given the scarcity of the natural product and uncertainty surrounding its structure, we aimed to understand the structural features of this toxin and their impact on bioactivity. By identifying which structural features render it significantly more potent than STX itself, we can devise pharmacological probes to investigate sodium channel functions and develop therapeutics. Herein, we describe results that begin to build an understanding of these structure–function relationships.

The chemist’s ability to synthesize the core of these toxins in the laboratory has efficiently evolved over the last 50 years.^[8–13]^ Only more recently have we been faced with an appreciation of the difficulties associated with functionalizing the toxin (particularly at C11).^[14]^ Indeed, the Nagasawa laboratory has recently reported a parallel investigation posing similar questions about the structure–function relationships in ZTX. By installing a vinyl group at C6, they successfully coordinated the introduction of an alkenyl-*N*-hydroxyamide at C13 for subsequent Ring-closing metathesis (RCM) to forge the macrocycle. Unfortunately, the proposed intramolecular RCM proved difficult, and they were only able to obtain a single STX macrocyclic analog with an 18-membered ring that was found to be inactive at inhibiting VGSCs.^[15]^

## 2. Results and Discussion

**Scheme 1.**
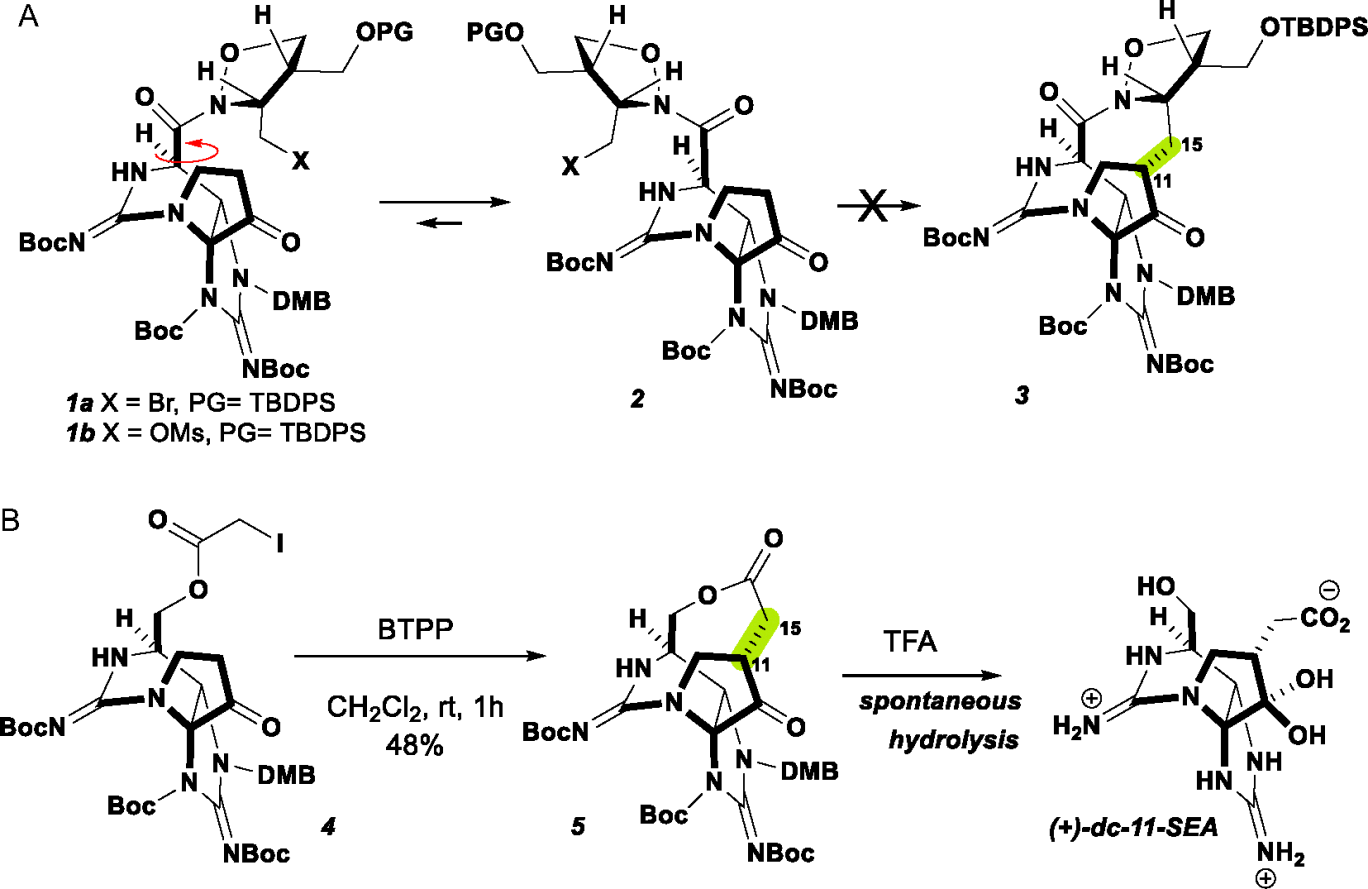
Previous synthetic explorations of the ZTX macrocycle. A) Unsuccessful direct alkylation due to unfavorable conformer. B) Spontaneous hydrolysis of macrocycle **5**.

Our interest in constructing these macrocycles led us to target the 9-membered macrocyclic forms of ZTX. Using chemistry developed in our lab, we previously attempted to construct the ZTX macrocycle (**3**) directly from the isoxazolidine amides **1a,b**^[16]^ by direct alkylation at C11 but were ultimately unsuccessful (Scheme 1A). We presumed C13 in the acid oxidation state prefers the conformer (**2**) that positions the oxazolidine away from the tricyclic core, precluding reaction with C6.

Similar observations were made by Nagasawa et al. unable to form smaller rings. We successfully demonstrated that C13 at the alcohol oxidation state could be appended with an α-haloacetate and that the C─C bond could be constructed between C11 and C15 by direct alkylation (Scheme 1B). This provided the 9-membered macrolactone **5**. Deprotection of this intermediate was accompanied by spontaneous hydrolysis to give the natural product (+)-11-saxitoxinethanoic acid.^[17]^ Therefore, we were unable to study the properties of the intact macrocycle.

**Scheme 2.**
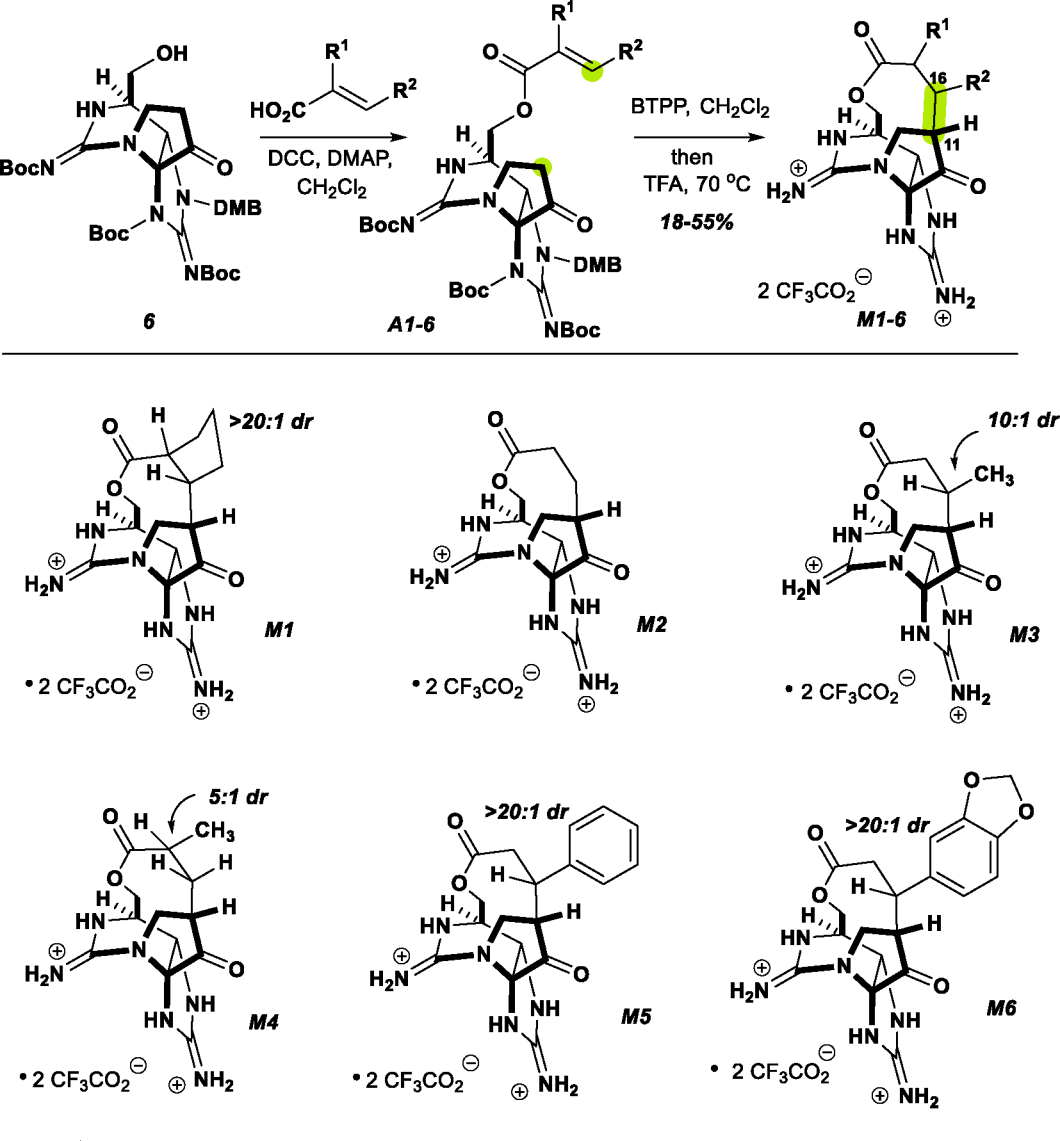
Preparation of macrocyclic ZTX analogs.

We reasoned that a subtle expansion of the macrocycle ring size would likely reduce the ring strain and correspondingly, spontaneous hydrolysis (Scheme 2). To this end, an acrylic acid could be coupled to the C13 alcohol of compound **6** under standard N,N’-Dicyclohexylcarbodiimide conditions. Exposure of the acrylic esters **A1–A6** to the phosphazene base, Phosphazene base P1-t-Bu-tris(tetramethylene), proved effective at triggering the intramolecular Michael addition, furnishing a single diastereomer of the corresponding macrolactone.

Global deprotection with TFA at 70 °C successfully removed all protecting groups, to give **M1–M6** while leaving the 10-membered lactone intact. This sequence allowed the preparation of macrolactones from unsubstituted, disubstituted, and mono-α- and mono-β-substituted acrylates in a similar fashion. Note, due to potential toxicity concerns, we chose to prepare a focused set of compounds, with only small amounts of material processed the bis-guanidinium ion product. These Michael additions appear to proceed in a thermodynamically controlled manner, wherein the bridging substituents are ultimately positioned exo- to the tricyclic core with a range of 5:1 → >20:1 diastereoselectivity correlated with steric demand.

**Figure 2.**
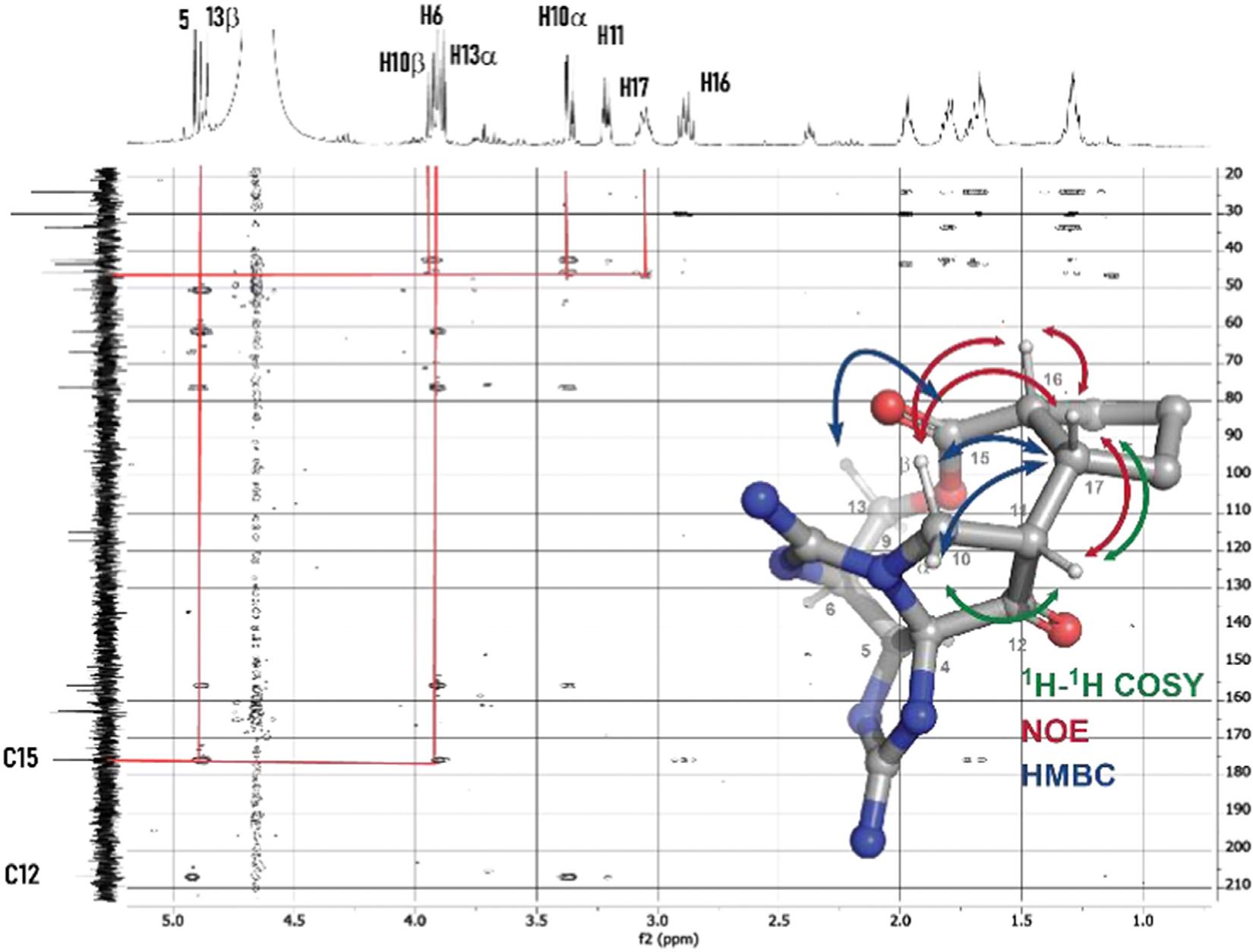
HMBC and NOE correlations observed with M1.

NMR analysis, including heteronuclear multiple bond correlation (HMBC) correlations observed between H13 → C15, H17 → H10, and H10 → H17 and ^1^H-^1^H-COSY (Correlation spectroscopy) correlations between H11 → H17, supports the presence of the C11─C17 bond and the intact macrocyclic structure (Figure 2). The nuclear overhauser effect (NOEs) observed between H16 ↔ H17, H17 ↔ H11, and H17 ↔ H10α are consistent with the lowest energy conformer (LEC) of the cis-exo isomer identified from DFT calculations (vide infra).

The most striking feature of the prepared analogs is that C12 exists exclusively in the keto-form. We initially expected that any carbonyl, flanked by two potent electron-withdrawing guanidinium ions, would be sufficiently electron poor and exist primarily in its hydrated form. To our surprise, the C12 ketone in these macrocycles is not hydrated, like in all other naturally occurring STX analogs; all synthetic analogs, **M1–M6**, were found to exist exclusively in the ketone form.

To probe this experimentally observed preference for ketone versus hydrated C12 position, we turned to DFT calculations to compute the difference in the Gibbs free energy for C12 hydration ($\Delta G_{\text{hyd}}$).^[18]^ As a first step, conformational ensembles were acquired for both the ketone and hydrated forms of **M1–M6**, as well as STX, GTX, dc-STX, ZTX, and water. All conformers were optimized by DFT at the ωB97xD/def2-SVP/SMD(water) level of theory.^[19]^ Single-point corrections were obtained (M062X-D3/def2-TZVP/SMD(water)) and used to compute the quasiharmonic corrected Gibbs free energy of each conformer.^[20,21]^ The global LECs were used to compute $\Delta G_{\text{hyd}}$ relative to STX $\left(\Delta G_{\text{hyd}}=0\;kcal\;{mol}^{-1}\right)$ by the following equation: $\Delta G_{\text{hyd}}=G_{\text{hydrate}}-\left(G_{\text{ketone}}+G_{\text{water}}\right)$.

**Figure 3.**
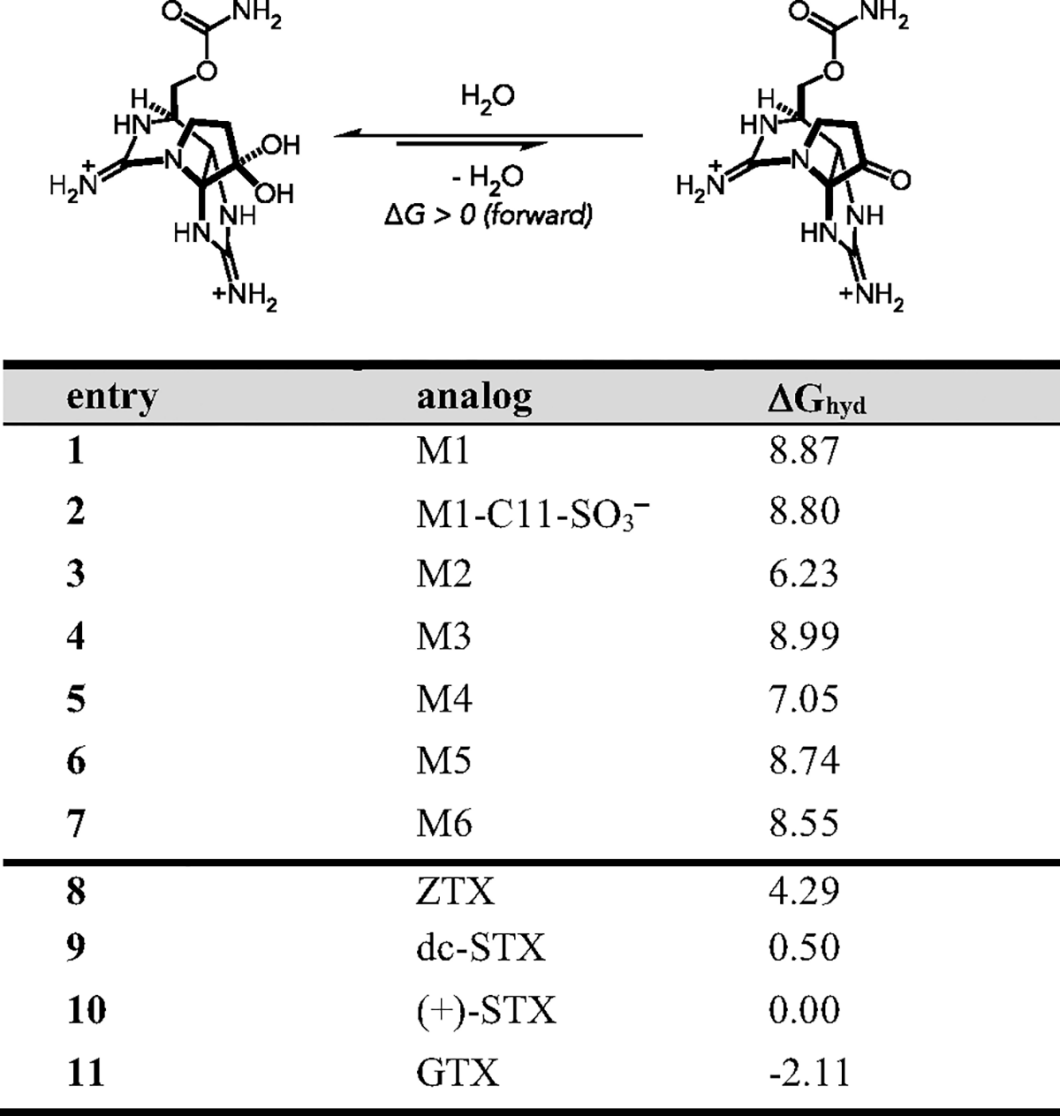
Difference in Gibbs free energy for C12 hydration for synthetic analogs and several bis-guanidinium ion toxins relative to (+)-STX ($\Delta G_{\text{hyd}}=0$). Positive ΔG values correspond to ketone energetic preference.

Initial observations revealed an unanticipated ≈11 kcal mol^−1^ spread in computed $\Delta G_{\text{hyd}}$ across these ten structurally similar analogs (Figure 3). Additionally, these solvent-corrected calculations estimate all synthetic analogs **M1–M6** to have a $\Delta G_{\text{hyd}}>+6\;kcal\;{mol}^{-1}$ consistent with the observation that these compounds exist exclusively in the keto-form.^[13]^ The absolute $\Delta G_{\text{hyd}}$ for both (+)-STX (−1.97) and dc-STX (−1.47) was negative, consistent with the known hydrated structures (Table S1, Supporting Information). To our surprise, the currently established structure of ZTX displays a computed $\Delta G_{\text{hyd}}=+4.29\;kcal{\;mol}^{-1}$ relative to (+)-STX (+2.32 kcal mol^−1^ absolute $\Delta G_{\text{hyd}}$). While **M1–M6** demonstrated an additional energetic preference for the ketone, these calculations suggest the proposed macrocyclic structure of ZTX should also exist primarily as the C12 ketone in its bis-guanidinium form, contrary to the reported hydrate structure. Note, the addition of the of the C11-sulfate to **M1** (hypothetical structure, entry 2) does not appreciably alter $\Delta G_{\text{hyd}}\;\left(<1\;kcal{\;mol}^{-1}\right)$ as compared to a >2 kcal mol^−1^ stabilization observed in analogs not containing a macrocycle (compare (+)-STX–GTX). This suggests that the presence of the sulfate is not sufficient to overcome the hydration penalty associated with the macrocycle.

**Figure 4.**
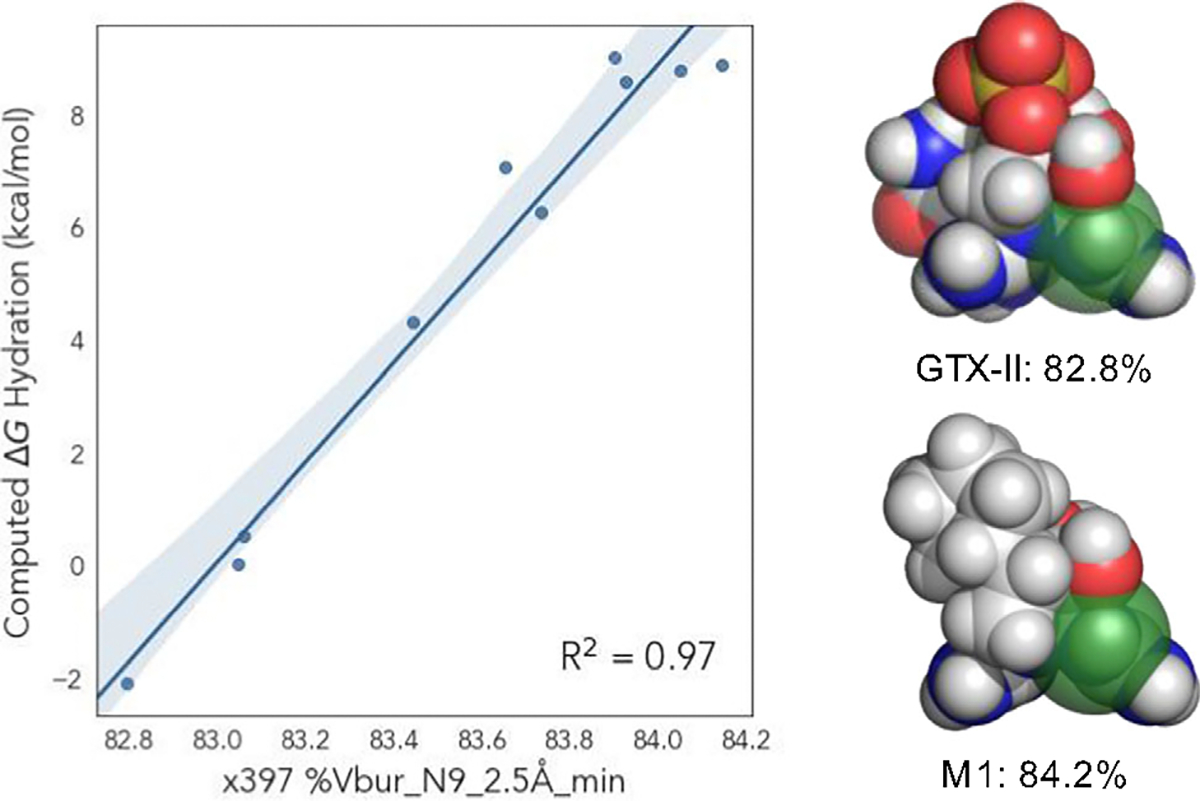
Univariate correlation between the buried volume at N9 (Vbur(N9)) and computed difference in Gibbs free energy of C12 hydration. Visualization of GTX-II and M1 demonstrate modest range corresponding to large changes in $\Delta G_{\text{hyd}}$.

To gain further insights into the structural features that led to the spread of computed hydration energies, we developed a simple univariate correlation composed of a single DFT-derived molecular feature of the hydrate for each analog. While several strong univariate correlations were observed (see Supporting Information for details), the buried volume centered on N9 at 2.5 Å (Vbur(N9)) proved highly effective at describing the relative C12 hydration trends (Figure 4). This model indicates increased steric bulk at N9 leads to a proportional increase in computed $\Delta G_{\text{hyd}}\;\left(R^{2}=0.97\right)$. However, the range of Vbur leading to these changes in computed $\Delta G_{\text{hyd}}$ is relatively small (≈1.5%), indicating small perturbations in the steric environment about N9 lead to significant changes in hydration energy. For example, the buried volume of GTX-II is 82.8% and favors the hydrate by −2.11 kcal mol^−1^; M1 displaysa +8.87 kcal mol^−1^ preference for the ketone with a fractionally larger 84.2% buried volume.

Interestingly, the major factor contributing to the observed changes in buried volume was the orientation of O12α relative to N9 and correspondingly O12β to N3. Thus, we reasoned subtle changes in buried volume may be more directly correlated to stabilizing/destabilizing orbital interactions in the hydrate.

**Figure 5.**
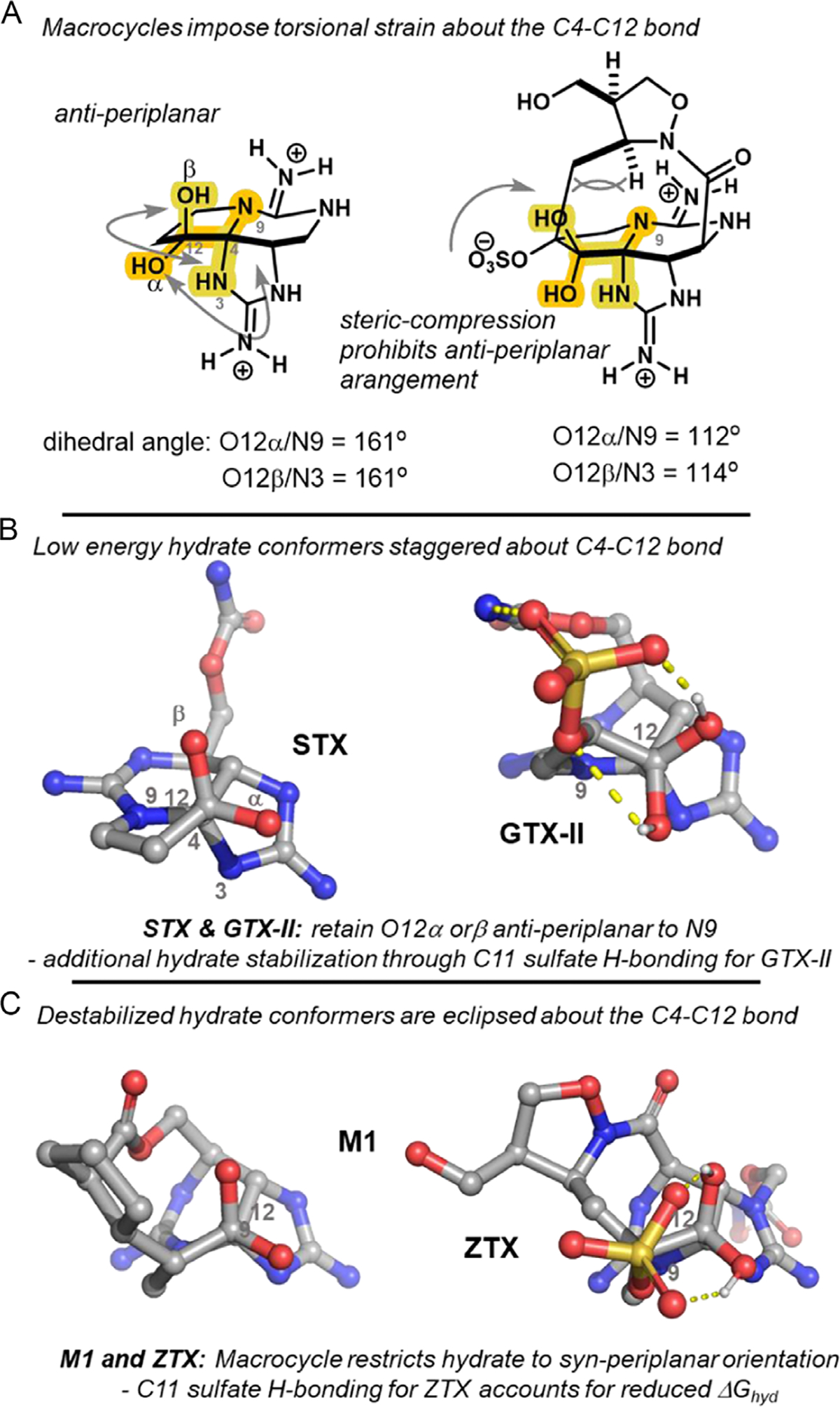
Dihedral angles of N9–C4–C12–O12α for a series of STX analogs. A) An ideal antiperiplanar arrangement observed for GTX-II and STX. B) Macrocyclic connection at C11 restricts the hydrate to a less stable syn-periplanar arrangement. C) Macrocycle destablized hydrate conformers.

Indeed, analysis of the minimum buried volume conformer for a series of analogs revealed a key structural feature influencing hydration at C12. By orienting the C4─C12 bond into the plane of the page, an ideal antiperiplanar arrangement is observed between N9–C4–C12–O12α for compounds lacking a macrocyclic bridge (e.g., GTX-II and STX, Figure 5A). This minimizes torsional strain in the hydrate and all substituents on C4 and C12 are staggered. The increased hydration preference found for GTX-II suggests H-bonding between the C11 sulfate and hydrate provides additional stabilization of the hydrate form (Figure 5B).

Conversely, all analogs with a macrocyclic connection at C11 bear a syn-periplanar arrangement of N9–C4–C12–O12α, presumably due to steric compression introduced by the macrocycle (Figure 5A,C). This syn-periplanar conformation of both O12α/N9 and O12β/N3 increases torsional strain in the hydrate and thus the hydration energy. For example, both ZTX and M1 demonstrate significantly reduced dihedral angles of −7.0° and 14.2° with computed hydration energies of +4.29 and +8.87 kcal mol^−1^, respectively. The reduced $\Delta G_{\text{hyd}}$ for ZTX in comparison with the other synthetic analogs also appears to arise from stabilization of the hydrate through an H-bonding network with the C11 sulfate as in GTX. Correlation of the C12─O12b bond length to stabilization of the hydrate was also observed, suggesting that the staggered conformers are additionally stabilized by electronic interactions ($n_{O12\beta }\to {\sigma^{*}}_{CN3},n_{O12\alpha }\to {\sigma^{*}}_{CN9}$).

Taken together, computational analysis suggests that multiple factors contribute to the overall stability of the hydrate relative to the ketone in these toxins. However, the large energetic difference between the acyclic and macrocyclic analogs (≥≈3.0 kcal mol^−1^) suggests the macrocyclic linkage generally disfavors hydration of the ketone. Apparently, this steric compression that results in an eclipsing conformer of the hydrate is accurately described by Vbur(N9).

With insights into the structural differences between the synthetic and natural analogs, analogs **M1–M6** were evaluated by constellation pharmacology, a calcium fluorescence imaging method, which has proven to be a powerful screening tool for neuronal drug discovery.^[22]^ We evaluated the bioactivity of analogues that lack the C12 hydrate, previously identified as a critical element for VGSC binding affinity.

**Figure 6.**
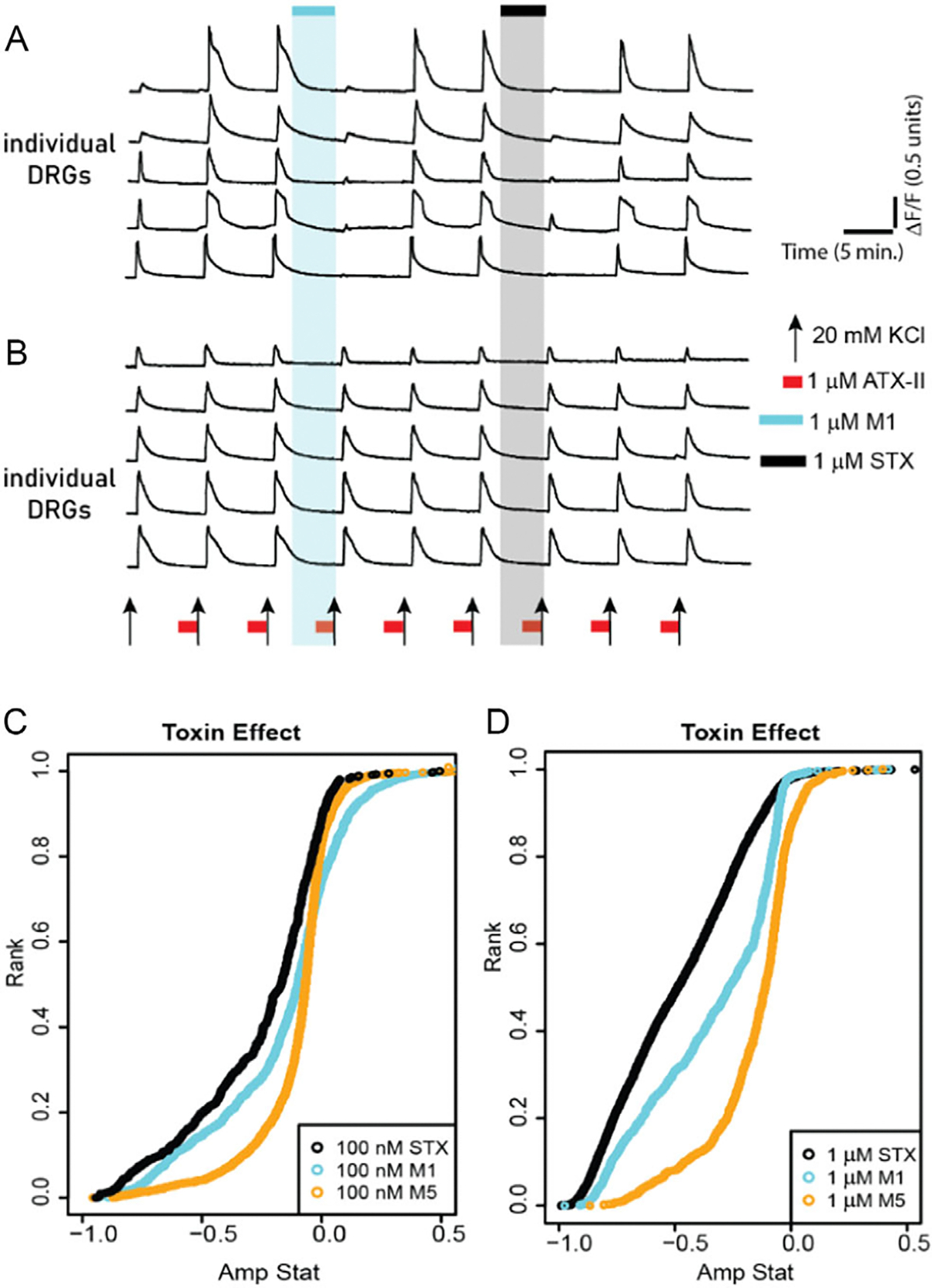
Activity of analog **M1** and **M5** compared with saxitoxin. Intracellular calcium levels were monitored in ≈1000 individual DRG neurons in response to pharmacological stimuli. A) Selected calcium traces from five neurons in which analog **M1** and saxitoxin blocked KCl + ATXII-induced calcium influx. B) Representative calcium traces from five neurons that were not modulated by analog **M1** or saxitoxin. C) CDF graph showing the activity of analog **M1** and **M5** compared with saxitoxin, tested at 100 nM. D) CDF graph comparing the activity of M1, M5, and saxitoxin tested at 1 μM. Calcium traces in (A) and (B) are from the same experiment. *n* = 3 trials/concentration, 1000 neurons per trial.

Experiments were performed on primary neuronal cell culture obtained from mouse dorsal root ganglion (DRG) as previously described^[23,24]^ (Figure 6). The intracellular calcium levels were monitored in response to elevating extracellular KCl concentrations. To assess VGSC activity, we elevated KCl in the presence of 1 micromolar ATX-II, a potent anemone derived neurotoxin, which delays the inactivation of sodium channels. In general, VGSCs activate and deactivate very fast and shorter than the capture rate. The coapplication of ATX-II with KCl increases the magnitude of calcium influx to the depolarizing stimulus (20 mM KCl) (Figure 6A). After establishing two control pulses, 1 μM testing compound was applied and the effects were monitored on the subsequent depolarizing pulse. An active VGSC blockage results in a suppressed Ca^2+^ signal. After washing out the test compound, two additional control pulses were recorded followed by the addition of (+)-STX. The amplitude of the Ca^2+^ response inhibited by the test compound was compared with the activity of (+)-STX.

All analogs were tested at 1 μM and we identified that analog **M2, M3,** and **M4** did not attenuate KCl+ATX-II-induced Ca^2+^ response, while **M6** modestly blocked the calcium signals. However, analogs **M1** and **M5** significantly blocked Ca^2+^ influx. As shown in Figure 6, **M1** blocked KCl + ATX-II-induced calcium influx, suggesting its activity as a sodium channel blocker. Depolarization-induced calcium signals reverted immediately after washing out **M1** (two subsequent pulses to monitor the reversal of **M1** activity), suggesting that **M1** has a fast off-rate. To further validate the activity of M1 as a sodium channel blocker, we applied 1 μM (+)-STX and observed similar effects on the same population of neurons that were targeted by **M1**. The effects of (+)-STX were also quickly reversible. The bioactivity of each compound was simultaneously evaluated on ≈1000 indiidual neurons in culture. Figure 6B shows subsets of neurons that were unaffected by ATX-II and were not targeted by **M1** and (+)-STX. These results suggest a common mechanism of action between analogs and (+)-STX-block of VGSCs.

Both **M1** and **M5** were active at 1 μM and 100 nM concentrations with analog **M1** proving significantly more active at lower concentrations. To evaluate the frequency of neurons targeted by **M1** and the magnitude of calcium signals blocked, we plotted the data in the cumulative distribution function (CDF) graphs (as shown in Figure 6C,D). Each point on the curve is a single neuron and the graph shows the distribution of responses from ≈1000 neurons, ranging from cells that were most potently blocked by test compound to the least blocked. As shown, (+)-STX was the most potent blocker of the three compounds tested and **M1** displayed a very similar inhibition profile at 100 nM. Primary culture contains different neuronal cell types, and each cell type expresses a diversity of sodium channel complement. We demonstrate that analogs **M1** and **M5** are bioactive in native neurons, with comparable activity to STX.

## 3. Conclusion

In conclusion, a range of macrocyclic analogs of saxitoxin were successfully synthesized through an intramolecular Michael addition strategy. In doing so, we uncovered an important structural requirement to form the hydrate at C12 in these toxins. A new constellation pharmacology model was used to observe the bioactivity of analogs M1 and **M5** on VGSCs expressed in sensory DRG neurons. We found that the analogs have biological and pharmacological activity similar to the natural product (+)-STX, despite being found exclusively in ketone form. By using this analysis platform, new avenues to further characterize the unique pharmacology of these analogs have been opened. Taken together, these results encourage the continued synthesis and characterization of structurally diverse (+)-STX analogs to study the pharmacology of VGSCs. These results provide an opportunity to reconsider alternative structures for ZTX to confirm the exquisite structural and pharmacological properties of this natural product. In addition, studies to understand the structure–function relationships for the VGSC inhibitory activity of these macrocyclic ketone analogs are ongoing.

## Supplementary Material

Supplemental data

## Data Availability

The data that support the findings of this study are available in the supplementary material of this article.
