# Supplementary material for: Macrocycles of Saxitoxin: Insights into the Structure of Zetekitoxin AB
Source: Chembiochem. Author manuscript; Available in PMC 2025 May 29. (PMC12118334; doi:10.1002/cbic.202500170)
Supplement: Supplemental data [file NIHMS2076534-supplement-Supplemental_data.pdf]

Macrocyclic Analogs of (+)-Saxitoxin: insights into the structure of Zeteketoxin AB

Authors Wenyuan Li, Srinivas R. Paladugu, Jordan P. Liles. Manju Karthikeyan, Kevin Chase, Shrinivasan Raghuraman, Matthew Sigman and Ryan E. Looper*

Supporting Information

1. General Experimental Considerations:

Solvents as dichloromethane (CH_2Cl_2), tetrahydrofuran (THF), dimethylformamide (DMF) were degassed with N_2 and passed through a solvent purification system. Methanol (MeOH) was distilled from activated molecular sieves prior to use. Diglyme was commercially available.

Mass spectroscopy data were obtained at the University of Utah Mass Spectrometry Facility. ^{1}HNMR and ^{13}CNMR spectra data were obtained at 500 MHz and 125 MHz, or 400 MHz and 101 MHz, respectively. Proton peaks for ^{1}HNMR were reported as chemical shift (δ, ppm), multiplicity (s = singlet, d = doublet, t = triplet, dd = doublet of doublet, m = multiplets, br= broad), coupling constants (Hz), integration, and relative to $CDCl_3$ peak (7.26 ppm), D_2O peak (4.80 ppm), deuterated MeOH-d_4 (3.31 ppm), dimethylsulfoxide-d_6 (2.49 ppm). 13CNMR were reported as chemical shift (δ, ppm), and relative to $CDCl_3$ peak (77.0 ppm), deuterated MeOH-d_4 (49.1 ppm), and dimethylsulfoxide-d_6 (39.5 ppm). Optical rotations were collected on Perkin-Elmer PE-343 Polarimeter and reported as: $[\alpha]^{20}_{D}$ (c: g/100 mL, in solvent $CHCl_3$). Mass spectra were determined on a Micromass Quattro II (ESI/APCI-TOF) for HRMS at the University of Utah Mass Spectrometry Facility.

Experimental procedures:

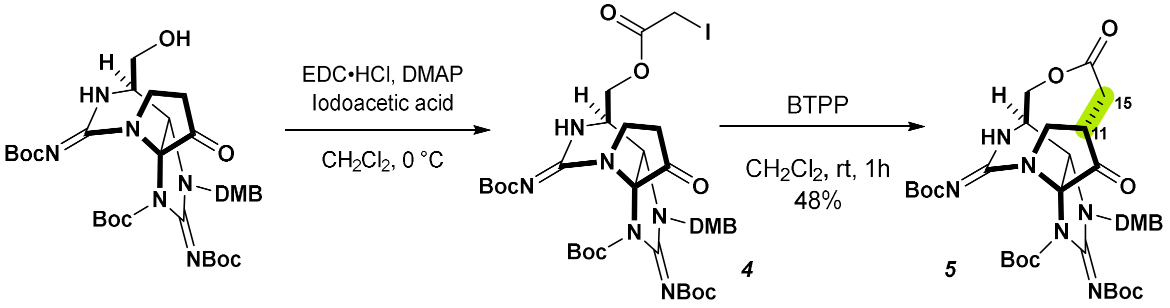

Synthesis of tricyclic iodo-acetate (tert-butyl (2E,3aS,4R,6Z,10aR)-2,6-bis((tert-butoxycarbonyl)imino)-3-(2,4-dimethylbenzyl)-4-((2-iodoacetoxy)methyl)-10-oxooctahydro-1H,8H-pyrrolo[1,2-c]purine-1-carboxylate) (4)

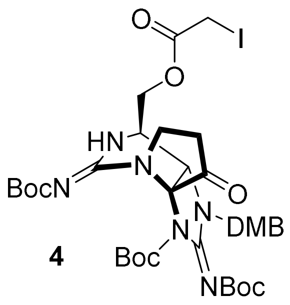

4

To a solution of the ketone-alcohol (1.3625 g, 1.98 mmol, 1 equiv.) in CH_2Cl_2 (20 mL) at 0 °C was added EDC·HCl (N-(3-Dimethylaminopropyl)-N′-ethylcarbodiimide hydrochloride) (0.759 g, 3.96 mmol, 2 equiv.), iodoacetic acid (0.5523 g, 2.97 mmol, 1.5 equiv.) and DMAP (4-Dimethylaminopyridine) (0.242 g, 1.98 mmol, 1 equiv.). The reaction mixture was allowed to stir for 1h at 0 °C. The mixture was diluted with CH_2Cl_2 then was washed with water. The aqueous layer was

extracted with 2x CH_2Cl_2 and the combined organic layers were dried over Na_2SO_4 and concentrated under reduced pressure to give a colorless foam that was used in next step without further purification

Synthesis of macrocyclic lactone (tert-butyl (2E,4R,6Z,10aR)-2,6-bis((tert-butoxycarbonyl)imino)-3-(2,4-dimethylbenzyl)-10,12-dioxooctahydro-1H,8H-9,4-(ethanooxymethano)pyrrolo[1,2-c]purine-1-carboxylate) (5)

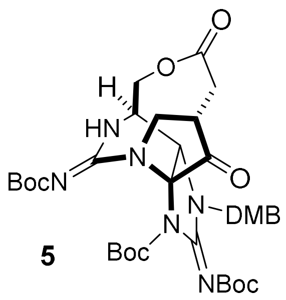

5

To a solution of tricyclic iodo-acetic acid (0.80 g, 0.9338 mmol, 1 equiv.) in CH_2Cl_2 (10 mL) was added BTTP (tert-Butylimino-tri(pyrrolidino)phosphorane) (0.57 mL, 1.868 mmol, 2 equiv.) at room temperature and the mixture was allowed to stir for 40 min. The reaction mixture was concentrated under reduced pressure. Purification by flash chromatography gave macrocyclic lactone (0.373 g, 55%) as a colorless foam.

TLC Rf = 0.2 (3:2 EtOAc:Hexanes);

$[\alpha]^{20}_D$ = –77.7° (c = 0.8, $CHCl_3$);

^{1}H NMR (400 MHz, $CDCl_3$) δ 9.23 (s, 1H), 7.26 (d, J = 8.3 Hz, 1H), 6.48 (dd, J = 8.4, 2.4 Hz, 1H), 6.41 (d, J = 2.3 Hz, 1H), 5.04 (d, J = 15.5 Hz, 1H), 4.99 (d, J = 12.3, 1H), 4.22 (d, J = 15.5 Hz, 1H), 4.17 (dd, J = 11.8, 6.5 Hz, 1H), 4.07 (s, 1H), 3.96 (d, J = 11.8 Hz, 1H), 3.83 (d, J = 4.3 Hz, 1H), 3.78 (d, J = 1.2 Hz, 6H), 3.42 (dd, J = 12.3, 4.5 Hz, 1H), 2.99 (q, J = 4.3 Hz, 1H), 2.80 (dd, J = 13.1, 3.4 Hz, 1H), 2.47 (dd, J = 13.2, 4.8 Hz, 1H), 1.47 (s, 9H), 1.46 (s, 9H), 1.44 (s, 9H);

^{13}C NMR (126 MHz, $CDCl_3$) δ 207.2, 168.0, 163.8, 161.0, 160.1, 158.1, 157.9, 150.1, 131.5, 113.8, 105.2, 98.6, 86.6, 79.4, 79.0, 77.3, 77.3, 77.1, 76.8, 76.7, 66.5, 61.1, 56.0, 55.6, 55.4, 50.1, 48.1, 42.6, 41.0, 37.3, 29.7, 28.4, 28.3, 28.3, 28.0 ppm.

HRMS (ESI) calcd for $C_{35}H_{49}N_6O_{11}$ (M+H): 729.3454, found: 729.3459.

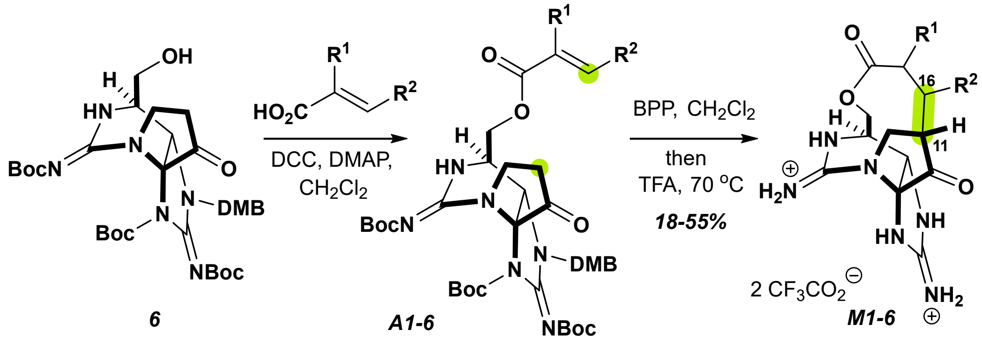

General procedure of synthesis of macrocycles (M1-M6)

To a solution of the tricyclic ketone (50 mg, 0.0726 mmol, 1 equiv.) in dry CH_2Cl_2 (1 mL) was added acrylic acid (0.109 mmol, 1.5 equiv.), EDC·HCl (N-(3-Dimethylaminopropyl)-N′-ethylcarbodiimide hydrochloride) (28 mg, 0.145 mmol, 2 equiv.) and DMAP (4-Dimethylaminopyridine) (14 mg, 0.109, 1.5 equiv.) at 0 °C. The mixture was allowed to stir for 1-4 h. Then the mixture was diluted with CH_2Cl_2 (2 mL) and washed with H_2O. The aqueous layer was extracted with CH_2Cl_2 (2 mL x2), and the combined organic layers were dried in Na_2SO_4 and

concentrated under reduced pressure to give white foam, which was used in next step without further purification. The white foam was dissolved in dry CH_2Cl_2 (1mL) and added BTPP (40 μL, 0.128 mmol, 2 equiv.) at room temperature. The mixture was stirred for 40 min – 2h, then concentrated under reduced pressure, and purified by flash column chromatography to give N-Boc macrocycles **A1-A6** as white solids.

Trifluoroacetic acid (3mL, 0.008M) was added to the lactone (19 mg, 0.0269 mmol) and heated to 70 °C for 72 h. The reaction mixture was concentrated under reduced pressure, co-evapurated with CH_2Cl_2 until dried. The resulting mixture was diluted with H_2O (1mL) and washed with CH_2Cl_2 (2mL x3). The aqueous layers were combined and filtered through a 25-micron PTFE Acrodisc® syringe filter. The filter was washed with additional 1 mL of H_2O. The combined filtrates were evaporated under reduced pressure to give synthetic macrocyclic analogs **M1-M6**.

Cyclopentanelactonemacrocycle (A1) (tert-butyl (2E,3aR,6aS,13R,15Z)-2,15-bis((tert-butoxycarbonyl)imino)-1-(2,5-dimethoxybenzyl)-10,16-dioxododecahydro-13,4-(epiminomethano)-3a,6-methanocyclopenta[i]imidazo[4,5-d][1]oxa[6]azacycloundecine-3(2H)-carboxylate)

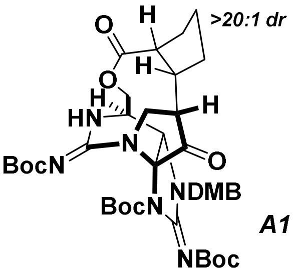

A1

55% yield 2 steps;

TLC Rf = 0.5 (2:3 Hexanes: EtOAc);

$[\alpha]^{20}_D = -48.1°$ (c = 1, $CHCl_3$);

^{1}H NMR (500 MHz, $CDCl_3$) δ 9.33 (s, 1H), 7.25 (dd, *J* = 8.4, 2.6 Hz, 1H), 6.52 (dt, *J* = 8.3, 2.6 Hz, 1H), 6.47 (t, *J* = 2.2 Hz, 1H), 5.11 – 4.99 (m, 1H), 4.28 – 4.20 (m, 1H), 4.16 (td, *J* = 11.8, 7.9 Hz, 1H), 3.85 – 3.79 (m, 7H), 3.79 – 3.73 (m, 1H), 3.45 (dd, J = 12.3, 4.1 Hz, 1H), 3.03 (ddq, *J* = 21.0, 10.2, 3.6 Hz, 1H), 2.07 – 1.98 (m, 2H), 1.92 – 1.76 (m, 2H), 1.57 – 1.48 (m, 27H), 1.48 – 1.38 (m, 2H);

^{13}C NMR (126 MHz, $CDCl_3$) δ 207.98, 172.82, 172.61, 163.91, 161.13, 160.93, 160.71, 158.44, 158.33, 158.03, 156.90, 151.87, 150.56, 131.58, 131.34, 114.87, 114.02, 105.10, 104.98, 98.60, 98.58, 85.88, 79.42, 79.04, 78.74, 77.32, 77.27, 77.07, 76.92, 76.81, 75.10, 66.92, 66.74, 61.86, 60.86, 60.38, 55.60, 55.57, 55.39, 49.07, 48.96, 48.63, 47.84, 47.15, 45.56, 43.28, 43.26, 42.90, 42.41, 41.89, 39.74, 33.95, 33.83, 30.32, 29.71, 28.53, 28.37, 28.34, 28.23, 28.16, 28.03, 27.86, 27.81, 25.64, 24.96, 24.84, 24.58, 21.04, 14.19.;

HRMS (ESI) calc for $C_{39}H_{55}N_6O_{11}$ (M+H): 783.3929, found 783.3937

Cyclopentanelactonemacrocycle (M1) ((3aS,6aS,13R)-10,16-dioxododecahydro-13,4-(epiminomethano)-3a,6-methanocyclopenta[i]imidazo[4,5-d][1]oxa[6]azacycloundecine-2,15(3H)-diiminium)

^{1}H NMR (500 MHz, D_2O) δ 4.91 (d, *J* = 1.0 Hz, 1H), 4.90 – 4.83 (m, 1H), 3.96 – 3.86 (m, 3H), 3.37 (dd, *J* = 12.3, 3.4 Hz, 1H), 3.21 (dt, *J* = 9.6, 3.3 Hz, 1H), 3.05 (dtd, J=10.4, 7.6, 3.2 Hz 1H), 2.88 (ddd, *J* = 10.6, 10.6, 7.7 Hz, 1H), 1.97 (tt, *J* = 9.4, 3.8 Hz, 1H), 1.85 – 1.76 (m, 1H), 1.75 – 1.61 (m, 2H), 1.36 – 1.22 (m, 2H).;

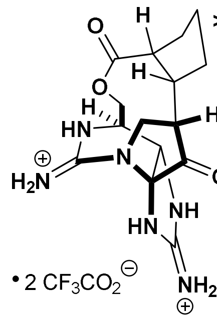

^{13}C NMR (126 MHz, D_2O) δ 207.09, 175.83, 163.00, 162.72, 156.01, 117.40, 115.09, 76.46, 66.86, 61.46, 50.37, 45.96, 43.49, 42.43, 33.69, 30.02, 24.15.;

HRMS (ESI) calc for $C_{15}H_{21}N_6O_3$ (M+H): 333.1670, found 333.1675

Lactonemacrocycle (A2) (tert-butyl (2E,4R,6Z,10aR)-2,6-bis((tert-butoxycarbonyl)imino)-3-(2,5-dimethoxybenzyl)-10,13-dioxooctahydro-1H,8H-9,4-(propanooxymethano)pyrrolo[1,2-c]purine-1-carboxylate)

18% yield 2 steps;

TLC Rf = 0.35 (2:3 Hexanes: EtOAc);

$[\alpha]^{20}_{D}$ = –93.0° (c = 1, $CHCl_3$);

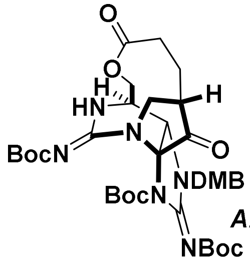

^{1}H NMR (500 MHz, $CDCl_3$) δ 9.34 (s, 1H), 7.25 (d, *J* = 8.3 Hz, 1H), 6.50 (dd, *J* = 8.3, 2.4 Hz, 1H), 6.46 (d, *J* = 2.3 Hz, 1H), 4.98 (d, *J* = 15.2 Hz, 1H), 4.27 (d, *J* = 15.2 Hz, 1H), 4.20 (dd, *J* = 11.7, 8.9 Hz, 1H), 4.02 – 3.97 (m, 3H), 3.96 (s, 1H), 3.82 (s, 6H), 3.77 (s, 1H), 3.17 (tt, *J* = 9.0, 2.8 Hz, 1H), 2.59 (ddd, *J* = 16.8, 12.4, 4.5 Hz, 1H), 2.41 (ddt, *J* = 16.9, 13.0, 4.1 Hz, 2H), 2.06 – 1.96 (m, 1H), 1.52 – 1.46 (m, 27H);

^{13}C NMR (126 MHz, $CDCl_3$) δ 209.51, 170.19, 164.04, 162.05, 161.17, 158.30, 157.94, 151.25, 150.42, 131.48, 113.76, 105.04, 98.55, 85.94, 79.44, 78.84, 77.29, 77.04, 76.78, 75.98, 66.56, 61.38, 55.51, 55.40, 48.79, 48.58, 42.49, 41.72, 29.88, 29.69, 28.50, 28.35, 27.91, 25.28;

HRMS (ESI) calc for $C_{36}H_{51}N_6O_{11}$ (M+H): 743.3616, found: 743.3621

Lactonemacrocycle (M2) ((4R,10aS)-10,13-dioxohexahydro-1H,8H-9,4-(propanooxymethano)pyrrolo[1,2-c]purine-2,6-diiminium)

1H NMR (500 MHz, D_2O) δ 4.72 (s, 1H), 4.30 (d, J = 12.0 Hz, 1H), 4.20 (dt, J = 12.1, 2.1 Hz, 1H), 4.06 – 3.99 (m, 1H), 3.95 (s, 1H), 3.89 (dd, J = 12.5, 3.0 Hz, 1H), 3.20 – 3.13 (m, 1H), 2.58 (ddd, J = 17.0, 12.1, 4.6 Hz, 1H), 2.43 (dt, J = 17.4, 4.7 Hz, 1H), 2.38 – 2.28 (m, 1H), 2.07 (ddt, J = 14.1, 8.9, 5.0 Hz, 1H);

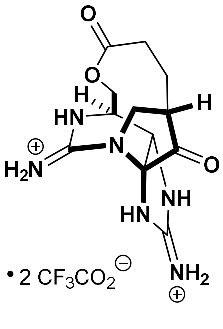

13C NMR (126 MHz, D_2O) δ 209.30, 172.48, 163.10, 162.81, 157.41, 156.95, 75.65, 69.54, 66.30, 61.78, 49.40, 47.00, 41.36, 29.25, 25.13;

HRMS (ESI) calc for $C_{12}H_{17}N_6O_3$ (M+H): 293.1362, found 293.1367

17-Methyllactonemacrocycle (A3) (tert-butyl (2E,4R,6Z,10aR,11R)-2,6-bis((tert-butoxycarbonyl)imino)-3-(2,5-dimethoxybenzyl)-11-methyl-10,13-dioxooctahydro-1H,8H-9,4-(propanooxymethano)pyrrolo[1,2-c]purine-1-carboxylate)

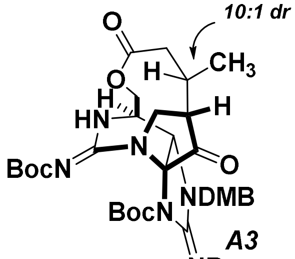

A3

34% yield 2 steps;

TLC Rf = 0.5 (2:3 Hexanes: EtOAc);

$[\alpha]^{20}_D$ = –57.4° (c = 1, $CHCl_3$);

^{1}H NMR (500 MHz, $CDCl_3$) δ 9.30 (s, 1H), 7.25 (d, *J* = 8.3 Hz, 1H), 6.53 – 6.46 (m, 2H), 4.91 (d, *J* = 15.0 Hz, 1H), 4.29 (d, *J* = 15.1 Hz, 1H), 4.24 – 4.12 (m, 2H), 4.03 (s, 1H), 3.87 (dd, *J* = 11.5, 3.4 Hz, 1H), 3.83 (d, *J* = 7.7 Hz, 6H), 3.78 (dd, *J* = 11.9, 2.3 Hz, 1H), 3.66 (s, 1H), 3.06 – 2.99 (m, 1H), 2.84 – 2.74 (m, 1H), 2.37 (dd, *J* = 14.4, 12.0 Hz, 1H), 2.29 (dd, *J* = 14.5, 4.8 Hz, 1H), 1.57 – 1.45 (m, 27H), 1.09 (d, *J* = 7.0 Hz, 3H);

^{13}C NMR (126 MHz, $CDCl_3$) δ 208.66, 169.95, 161.26, 158.39, 157.87, 156.78, 151.24, 150.44, 139.43, 131.61, 125.43, 113.74, 104.96, 98.52, 98.50, 85.90, 79.54, 78.81, 77.29, 77.24, 77.04, 76.79, 75.84, 66.13, 62.06, 55.48, 55.41, 49.86, 49.71, 49.10, 46.38, 42.16, 37.60, 36.71, 33.96, 33.32, 33.23, 29.70, 28.49, 28.35, 28.33, 28.03, 27.88, 25.62, 25.55, 24.96, 24.86, 23.36, 22.69, 17.66.

HRMS (ESI) calc for $C_{37}H_{52}N_6O_{11}$ (M+H): 757.3772, found: 757.3777

17-Methyllactonemacrocycle (M3) ((4R,10aS,11R)-11-methyl-10,13-dioxohexahydro-1H,8H-9,4-(propanooxymethano)pyrrolo[1,2-c]purine-2,6-diiminium)

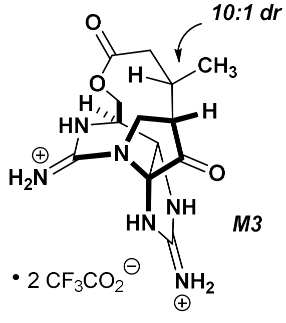

M3

^{1}H NMR (500 MHz, D_2O) δ 4.52 (d, *J* = 12.1 Hz, 1H), 4.19 – 4.05 (m, 3H), 4.05 – 3.98 (m, 1H), 3.95 (dd, *J* = 12.2, 3.7 Hz, 1H), 3.15 (ddd, *J* = 9.6, 3.7, 1.9 Hz, 1H), 2.82 (dtd, *J* = 15.0, 7.4, 3.8 Hz, 1H), 2.49 – 2.43 (m, 2H), 1.08 (d, *J* = 7.0 Hz, 3H).

^{13}C NMR (126 MHz, D_2O) δ 208.01, 171.97, 163.10, 162.81, 157.41, 157.38, 117.51, 115.19, 75.80, 66.87, 65.86, 62.17, 49.68, 49.06, 48.13, 44.69, 37.14, 36.77, 33.49, 21.61, 20.96.;

HRMS (ESI) calc for $C_{13}H_{19}N_6O_3$ (M+H): 307.1513, found 307.1519

16-Methyllactonemacrocycle(A4) (tert-butyl (2E,4R,6Z,10aR)-2,6-bis((tert-butoxycarbonyl)imino)-3-(2,5-dimethoxybenzyl)-12-methyl-10,13-dioxooctahydro-1H,8H-9,4-(propanooxymethano)pyrrolo[1,2-c]purine-1-carboxylate)

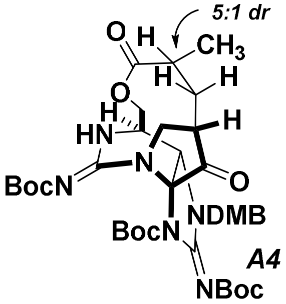

A4

46% yield 2 steps;

$[\alpha]^{20}_D$ = –120° (c = 1, $CHCl_3$);

^{1}H NMR (500 MHz, $CDCl_3$) δ 9.42 (s, 1H), 7.26 (d, *J* = 8.3 Hz, 1H), 6.50 (dd, *J* = 8.4, 2.3 Hz, 1H), 6.45 (d, *J* = 2.3 Hz, 1H), 5.07 (d, *J* = 15.5 Hz, 1H), 4.29 – 4.20 (m, 2H), 4.05 – 3.90 (m, 5H), 3.82 (d, *J* = 3.5 Hz, 7H),

3.20 – 3.10 (m, 1H), 2.83 (dqd, *J* = 10.9, 7.1, 3.4 Hz, 1H), 2.22 (ddd, *J* = 14.3, 10.4, 3.7 Hz, 1H), 1.85 (ddd, *J* = 14.4, 11.6, 4.9 Hz, 1H), 1.52 (s, 9H), 1.49 (s, 18H), 1.14 (d, *J* = 7.2 Hz, 3H);

^{13}C NMR (126 MHz, $CDCl_3$) δ 173.02, 161.02, 158.15, 131.77, 131.24, 113.94, 105.22, 105.10, 98.58, 98.51, 78.93, 77.30, 77.25, 77.04, 76.79, 74.97, 67.08, 60.13, 55.57, 55.53, 55.51, 55.39, 55.37, 49.08, 47.36, 47.07, 42.45, 41.40, 41.15, 37.15, 35.99, 35.35, 34.87, 33.96, 28.50, 28.36, 28.34, 28.00, 27.92, 27.90, 25.62, 24.96, 17.73, 17.59;

HRMS (ESI) calc for $C_{37}H_{52}N_6O_{11}$ (M+H): 757.3772, found: 757.3770

16-Methyllactonemacrocycle (M4) ((4R,10aS,12R)-12-methyl-10,13-dioxohexahydro-1H,8H-9,4-(propanooxymethano)pyrrolo[1,2-c]purine-2,6-diiminium)

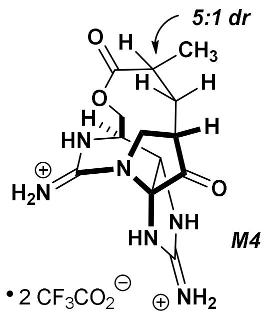

^{1}H NMR (500 MHz, d_2o) δ 4.35, 4.35, 4.33, 4.32, 4.15, 4.15, 4.13, 4.12, 4.01, 4.00, 4.00, 4.00, 3.99, 3.97, 3.97, 3.96, 3.95, 3.94, 3.93, 3.93, 3.92, 3.92, 3.90, 3.88, 3.79, 3.78, 3.76, 3.76, 3.11, 3.10, 3.10, 3.09, 3.09, 3.08, 3.08, 3.07, 3.07, 3.06, 3.06, 2.72, 2.71, 2.70, 2.70, 2.69, 2.68, 2.67, 2.32, 2.32, 2.30, 2.29, 2.29, 2.27, 2.27, 1.84, 1.83, 1.81, 1.81, 1.80, 1.79, 1.78, 1.01, 0.99;

^{13}C NMR (126 MHz, d_2o) δ 208.50, 175.52, 162.94, 162.66, 115.05, 76.37, 67.11, 52.06, 49.16, 41.16, 35.80, 35.16, 16.72.;

HRMS (ESI) calc for $C_{13}H_{19}N_6O_3$ (M+H): 307.1513, found 307.1527

17-Phenyllactonemacrycycle (A5) (tert-butyl (2E,4R,6Z,10aR,11R)-2,6-bis((tert-butoxycarbonyl)imino)-3-(2,5-dimethoxybenzyl)-10,13-dioxo-11-phenyloctahydro-1H,8H-9,4-(propanooxymethano)pyrrolo[1,2-c]purine-1-carboxylate)

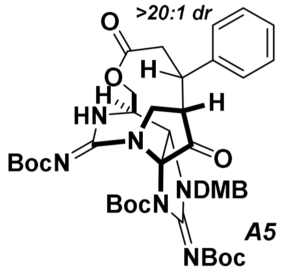

30% yield 2 steps;

TLC Rf = 0.5 (2:3 Hexanes: EtOAc);

$[\alpha]^{20}_D$ = –24.9° (c = 1, $CHCl_3$);

1H NMR (500 MHz, $CDCl_3$) δ 7.35 – 7.22 (m, 5H), 7.19 – 7.13 (m, 2H), 6.52 (dd, J = 8.3, 2.5 Hz, 1H), 6.49 (d, J = 2.4 Hz, 1H), 4.95 (d, J = 15.0 Hz, 1H), 4.35 (t, J = 10.3 Hz, 1H), 4.29 (d, J = 15.0 Hz, 1H), 4.25 – 4.15 (m, 2H), 4.07 (s, 1H), 3.94 (dd, J = 11.9, 2.1 Hz, 1H), 3.86 (s, 3H), 3.83 (d, J = 12.1 Hz, 1H), 3.83 (s, 3H), 3.72 (s, 1H), 3.37 (dd, J = 10.6, 7.4 Hz, 1H), 2.88 (dd, J = 14.9, 13.0 Hz, 1H), 2.52 (dd, J = 14.9, 4.1 Hz, 1H), 1.54 (s, 9H), 1.48 (s, 9H), 1.44 (s, 9H).

13C NMR (126 MHz, $CDCl_3$) δ 208.55, 169.49, 161.28, 158.40, 157.90, 154.00, 151.20, 150.35, 143.65, 131.64, 129.22, 127.39, 126.58, 113.69, 104.96, 98.53, 86.07, 79.67, 78.90, 77.30, 77.25, 77.05, 76.79, 75.63, 66.49, 62.24, 60.44, 55.54, 55.44, 49.61, 49.40, 47.37, 43.82, 42.17, 37.77, 33.94, 32.76, 30.80, 29.72, 28.48, 28.37, 27.82, 26.40, 25.60, 25.50, 25.30, 24.95, 24.70, 14.22.;

HRMS (ESI) calc for $C_{42}H_{55}N_6O_{11}$ (M+H): 819.3923, found: 819.3925

17-Phenyllactonemacrycycle (M5) ((4R,10aS,11R)-10,13-dioxo-11-phenylhexahydro-1H,8H-9,4-(propanooxymethano)pyrrolo[1,2-c]purine-2,6-diiminium)

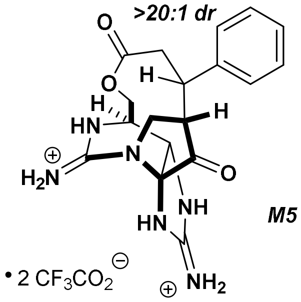

M5

1H NMR (500 MHz, D_2O) δ 7.35 – 7.10 (m, 5H), 4.80 (s, 1H), 4.38 (dt, J = 12.1, 1.7 Hz, 1H), 4.25 (dt, J = 12.1, 2.2 Hz, 1H), 4.21 – 4.04 (m, 2H), 3.97 (d, J = 2.1 Hz, 1H), 3.91 – 3.84 (m, 1H), 3.36 (ddd, J = 9.2, 4.1, 1.8 Hz, 1H), 2.99 (dd, J = 15.4, 13.2 Hz, 1H), 2.60 (ddd, J = 15.8, 4.0, 1.9 Hz, 1H).

13C NMR (126 MHz, D_2O) δ 207.22, 171.04, 163.07, 142.53, 129.25, 127.60, 126.54, 75.50, 66.19, 62.31, 49.37, 48.14, 45.51, 43.10, 36.27, 30.22.;

HRMS (ESI) calc for $C_{18}H_{21}N_6O_3$ (M+H): 369.1670, found: 369.1675

17-Phenyldioxolelactonemacrocycle (A6) (tert-butyl (2E,4R,6Z,10aR,11R)-11-(benzo[d][1,3]dioxol-5-yl)-2,6-bis((tert-butoxycarbonyl)imino)-3-(2,5-dimethoxybenzyl)-10,13-dioxooctahydro-1H,8H-9,4-(propanooxymethano)pyrrolo[1,2-c]purine-1-carboxylate)

30% yield 2 steps;

TLC Rf = 0.5 (2:3 Hexanes: EtOAc);

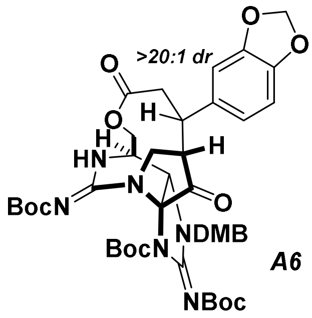

A6

^{1}H NMR (500 MHz, $CDCl_3$) δ 9.34 (s, 1H), 7.26 (d, *J* = 8.3 Hz, 1H), 6.74 (d, *J* = 8.0 Hz, 1H), 6.65 – 6.58 (m, 2H), 6.51 (dd, *J* = 8.3, 2.4 Hz, 1H), 6.49 (d, *J* = 2.4 Hz, 1H), 5.95 (s, 2H), 4.93 (d, *J* = 15.0 Hz, 1H), 4.29 (d, *J* = 15.1 Hz, 1H), 4.25 – 4.16 (m, 2H), 4.16 – 4.08 (m, 2H), 4.05 (s, 1H), 3.96 – 3.90 (m, 1H), 3.84 (d, *J* = 11.6 Hz, 8H), 3.79 – 3.67 (m, 3H), 3.37 – 3.30 (m, 1H), 2.88 – 2.77 (m, 1H), 2.49 (dd, *J* = 14.9, 4.1 Hz, 1H), 2.22 (s, 1H), 2.05 (s, 1H), 1.96 – 1.90 (m, 1H), 1.53 (s, 9H), 1.48 (s, 9H), 1.45 (s, 9H).;

^{13}C NMR (126 MHz, $CDCl_3$) δ 161.29, 158.40, 157.89, 151.22, 148.19, 146.70, 131.63, 119.51, 113.66, 108.67, 107.07, 104.95, 101.26, 98.53, 86.06, 79.67, 78.90, 77.31, 77.25, 77.05, 76.80, 66.47, 62.22, 60.44, 55.53, 55.43, 49.73, 49.61, 43.56, 42.21, 37.89, 33.94, 32.76, 30.80, 29.72, 28.48, 28.37, 27.83, 26.40, 25.60, 25.30, 24.95, 24.70, 14.21.;

HRMS (ESI) calc for $C_{43}H_{55}N_6O_{13}$ (M+H): 863.3822, found: 863.3822

17-Phenyldioxolelactonemacrocycle (M6) ((4R,10aS,11R)-11-(benzo[d][1,3]dioxol-5-yl)-10,13-dioxohexahydro-1H,8H-9,4-(propanooxymethano)pyrrolo[1,2-c]purine-2,6-diiminium)

1H NMR (500 MHz, D_2O) δ 6.80 – 6.50 (m, 3H), 5.81 (d, J = 1.7 Hz, 2H), 4.81 – 4.74 (m, 1H), 4.37 (ddd, J = 12.0, 8.2, 1.7 Hz, 1H), 4.27 – 4.20 (m, 1H), 4.10 (dd, J = 8.2, 3.8 Hz, 1H), 3.98 – 3.91 (m, 1H), 3.82 – 3.70 (m, 1H), 3.31 (ddd, J = 8.3, 4.4, 1.8 Hz, 1H), 2.93 (ddd, J = 15.3, 13.0, 8.5 Hz, 1H), 2.59 – 2.49 (m, 1H).

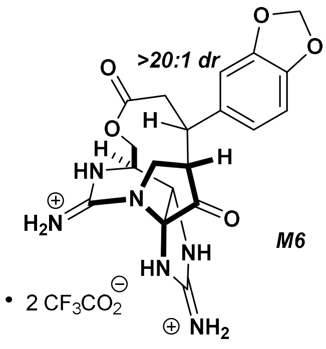

M6

13C NMR (126 MHz, D_2O) δ 207.11, 170.97, 162.82, 157.36, 147.57, 146.22, 136.54, 119.72, 116.45, 108.73, 107.11, 101.18, 75.50, 66.16, 62.30, 49.37, 48.43, 45.43, 42.86, 36.37.;

HRMS (ESI) calc for C19H21N6O5 (M+H): 413.1568, found 413.1574

2. Constellation pharmacology experiments

Calcium imaging based constellation pharmacology experiments were done as described before[1, 2]. Lumbar dorsal root ganglion (DRG) L1-L6 were isolated from 40-70 day old mice, digested with 0.25% trypsin for 20 minutes and dissociated to obtain primary cell culture plated on poly-D-lysine coated plates overnight, supplemented with minimal essential media + supplements. Cells were loaded with 2.5 µM FURA-2AM for an hour before experiment. Calcium imaging was performed using Nikon-Eclipse microscope and fluorescence from FURA-2 was monitored by ratiometric imaging. Video was captured every 2 s and the ratio of excitation wavelengths 340 nM/380 nM was monitored for an hour. In each trial, ~1000 neurons were simultaneously monitored. The changes to intracellular calcium levels were monitored in response extracellular application of pharmacological stimulus. Cells were stimulated with 20mM KCl for 15 s, every 7 minute. After the first KCl application, cells were pre-treated with 1uM ATX-II for 1 minute just before the next KCl stimulus pulse. Cells were incubated for 5-minutes with the test drug (either analog A4 or A5) at 100 nM or 1 uM concentration. The effects of the test drug were monitored on the subsequent KCl+ATX-II pulse. Two more stimulus pulses were given to observe recovery from the effect of test drug and cells were incubated with either 100 nM or 1uM saxitoxin (STX). The effects of test drug were compared with STX in the same experiment.

Analysis of data from Constellation pharmacology experiments

A single calcium imaging experiment measures the intracellular calcium concentration (ICC) for 100s-1000s of cells at 2 second intervals over a time course of minutes to hours. During the experiment specific modifications are made to the cell culture to test for induced changes in ICC. The ICC values for each cell across the entire experiment is referred to as the phenotype of the cell. 20 nM K^+ is added at regular intervals to induce a depolarization phenotype (KDP). The KDP is very specific for each cell and highly reproducible but is conditional upon a complex set of cellular variables.

We test for activity of a toxin of interest by incubating the cell culture in the toxin between K+ pulses and testing for a significant change in the phenotype of all or a subset of cells. A change that alters the KDP after incubation is referred to as a toxin effect (TXE).

-Relative ratio as a measure of the fractional impact of the toxin (Amp Stat). We use the relative ratio of the affected KDP to all other KDPs as a measure of the fractional impact of the toxin (Amp Stat). This is calculated as (KDPe-KDPmean)/(KDPe+KDPmean) where KDPe is the KDP of the pulse immediately after the application of the toxin and KDPmean is the mean of the KDP values across all other pulses. This value varies from -1, complete block to 1, complete amplification, and is roughly analogous to a signed R^2 value. The Amp Stat values from each cell are ranked from lowest to highest according to the Amp Stat values to form a cumulative distribution function (CDF) graph, used to visualize the distribution of the results.

3. ***Computational Details and Methods***

All Saxitoxin, **(+)-STX**, derivatives were subjected to the following general workflow: conformational searching in water, density functional theory (DFT) optimization in water, single point energy calculation in water, triple-zeta energy correction. Conformational searches were conducted for the ketone and hydrate forms of all reported analogs using Macromodel (part of the Schrödinger Suite) and the OPLS4 (release 2022-1)[3] force field (10,000 steps, 5 kcal/mol energy window) **in water**. At this stage, all conformers arising from this search were optimized at the ωb97XD/def2-SVP/SMD(water) level of theory using Gaussian 16 (Revision C.01).[4] All optimized geometries were verified by frequency computations as minima (zero imaginary frequencies) at the optimization level of theory. Single point calculations were run using M062X-D3/def2-TZVP/SMD(water) and parameters were acquired from these ground state structures (*vide infra*).[5] Triple-zeta energy corrections were computed using Paton GoodVibes (v3.2) and the quasi-harmonic Gibbs free energy correction.[6] NBO charges were calculated using NBO 3.1 at the M062X-D3/def2-TZVP/SMD(water) level as implemented in Gaussian 16.[7] ChelpG and Hirshfeld charges (and its derivatives) as well as NMR shifts (GIAO method) were computed as implemented in Gaussian 16 at the M062X-D3/def2-TZVP/SMD(water) level.[8–12] Water (H_2O) was computed in the same manner for hydration analysis. In total, 1215 conformers were optimized in this study. Coordinates for the lowest energy conformer (LEC) for each analog (ketone and hydrate forms), are included in the supplemental information. Coordinates for the minimum buried volume conformers $Vbur(C12)_{1.5Å}$ are also present (used for modeling). all related calculations can be found on Sigman GitHub organization page: https://github.com/SigmanGroup/STX_Hydration_Equilibria.. An atom numbering scheme for the atoms used for modeling is provided (**Figure S1**).

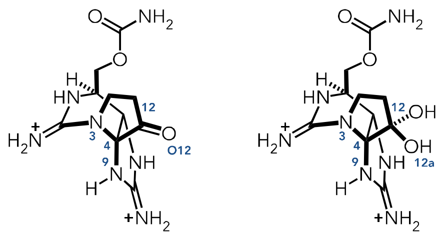

Figure S1. Atom labeling of (+)-STX used for DFT parameterization. Only atoms featuring correlations relevant to this study are displayed.

Gibbs free energy of ketone hydration ($\Delta G_{Hydration}$)

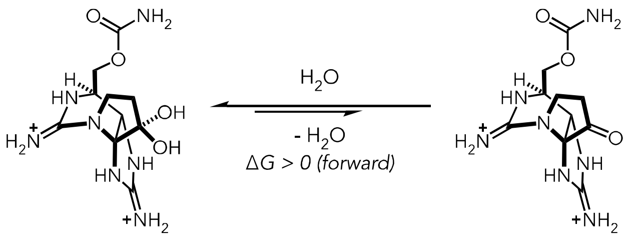

Scheme S1. Example of hydration equilibrium of (+)-STX in the presence of water. For this study, $\Delta G > 0$ denotes preference for the ketone.

Hydration energies ($\Delta G_{Hydration}$) were computed by first identifying the lowest energy conformer (LEC) for the ketone and hydrate of each respective analog. The energy for the LEC ketone was added to water, providing a corrected energy that could be compared directly to the hydrate. This sum was subtracted from the energy of the hydrate (**Scheme S1** & **Equation S1**). This provides positive ΔG values when the hydrate is disfavored, and negative ΔG values when the hydrate is favored. The full table can be found in the main text (**Table 1**). **Note**: gas phase-based conformer models have successfully been applied to the calculation of ketone hydration equilibria by computing the solvation energy (Henry's constant) upon placement in water.[13] However, this technique does not account for the effect of water on conformation, which was found to have a consequential impact on the computed ΔG of ketone hydration.

$$\Delta G_{\text{Hydration}} = G_{\text{Hydrate}} - (G_{\text{Water}} + G_{\text{Ketone}}) \quad \textbf{Equation S1}$$

Univariate Correlations of DFT Descriptors to Computed ΔG of Ketone Hydration ($\Delta G_{Hydration}$)

All univariate correlations were generated using the parameters found in **Supplemental_Document1.xlsx**. Sheet 1 contains all ketone descriptors and Sheet 2 contains all hydrate descriptors. Maximum and minimum descriptor values were obtained for each analog, as well as a LEC set of descriptors, and a 4D Boltzmann-weighted average set of descriptors. **In all cases, the minimum descriptor value is used for ketone modeling.** A combination of descriptor types was used for hydrate modeling.

All correlations were achieved by analyzing analogs in their respective ketone and hydrate forms to enable direct comparison. For example, *ketone* models use parameters derived from **(+)-STX** in its **ketone** form and *hydrate* models use parameters derived from **M1** in its **hydrate** form, respectively. This allows for direct comparison stabilizing/destabilizing interactions across the ketone and hydrate forms independently. Identification of strong univariate correlations was accomplished using an in-house Jupyter notebook with an R^2 threshold of 0.93 for the ketone model search and 0.97 for the hydrate model search.

Steric Ketone Model (Vbur(C12) at 1.5 Å)

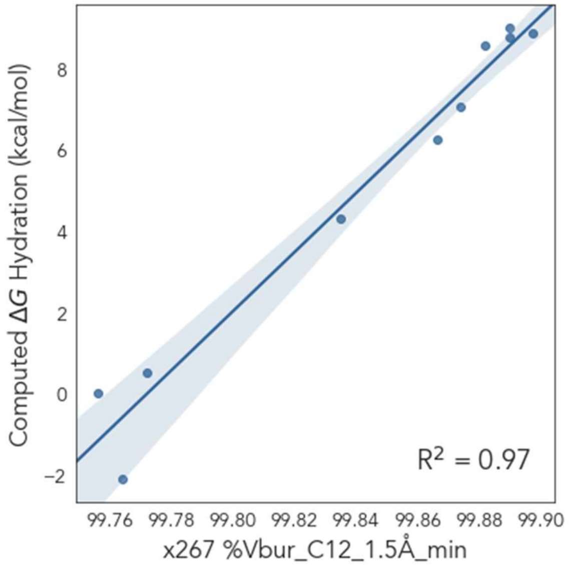

Vbur(C12, 1.5 Å) across several representative intermediates

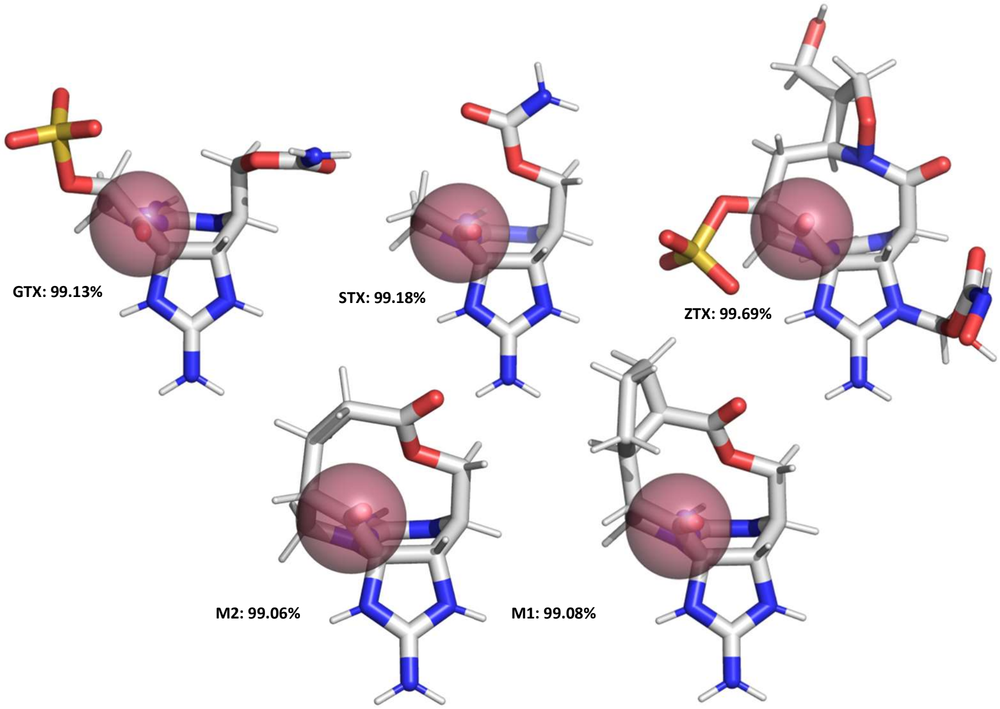

Sample Figure. Range of vbur is very small (~1%). Trend suggests substitution alpha to C12 is not directly influencing vbur (GTX), but the macrocycle impacts this term more heavily. Visualizing does not provide the greatest insight into this correlation since the differences are so small and vDW radii are used for vbur.

Additional Univariate Correlations Between Ketone Descriptors and ΔG of Hydration ($\Delta G_{Hydration}$)

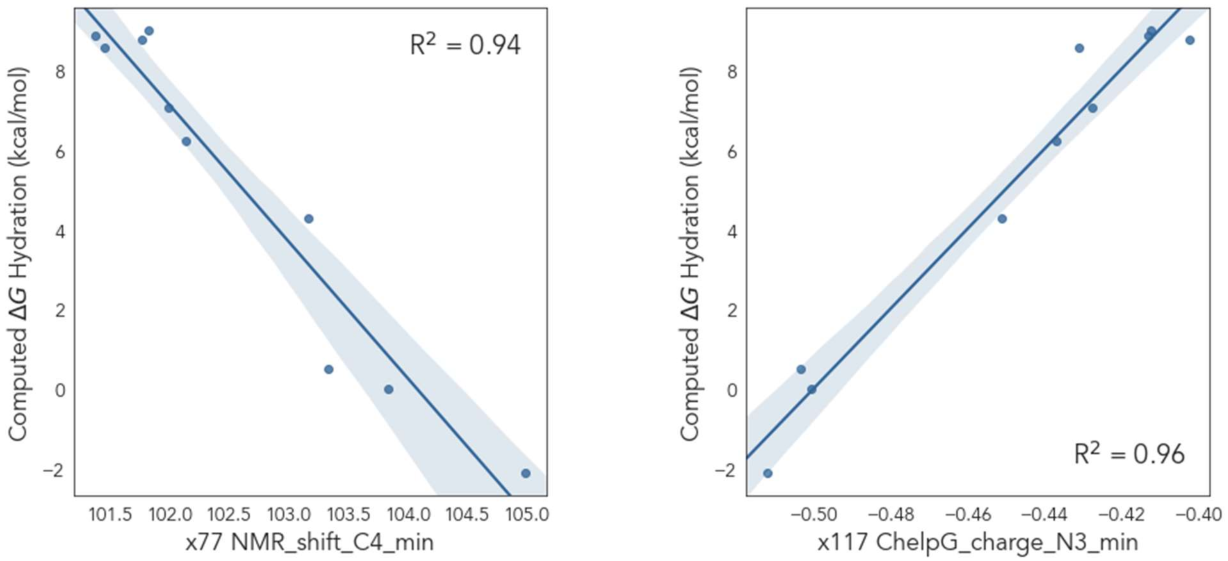

Figure S2. Correlation between ΔG of ketone hydration and computed NMR shift of C4 (left) and ChelpG charge of N3 (right).

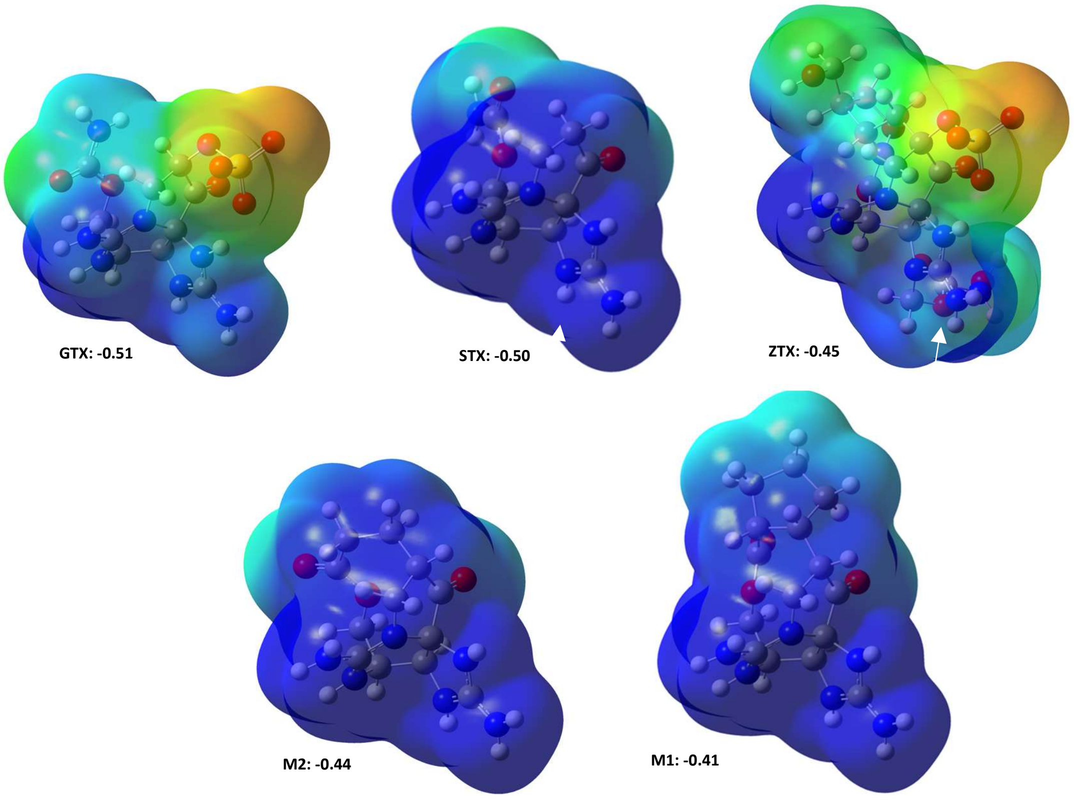

Figure S3. Trend of partial charge build up on N3 position visualized by electrostatic potential map (ESP).

Univariate Correlations Between Hydrate Descriptors and ΔG of Hydration ($\Delta G_{Hydration}$)

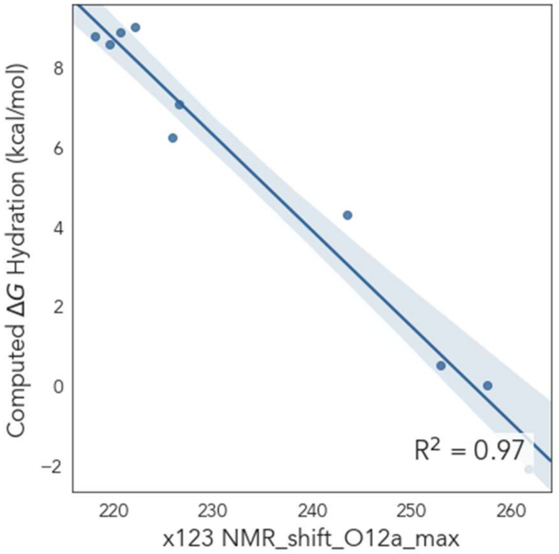

Figure S4. Correlation between ΔG of ketone hydration and computed NMR shift of O12α (left).

While a strong univariate correlation is observed with the computed NMR shift of O12α, this correlation was ultimately deemed difficult to interpret due to the complex quadrupolar interactions arising from ^{17}O bearing a spin of nucleus 5/2.[14]

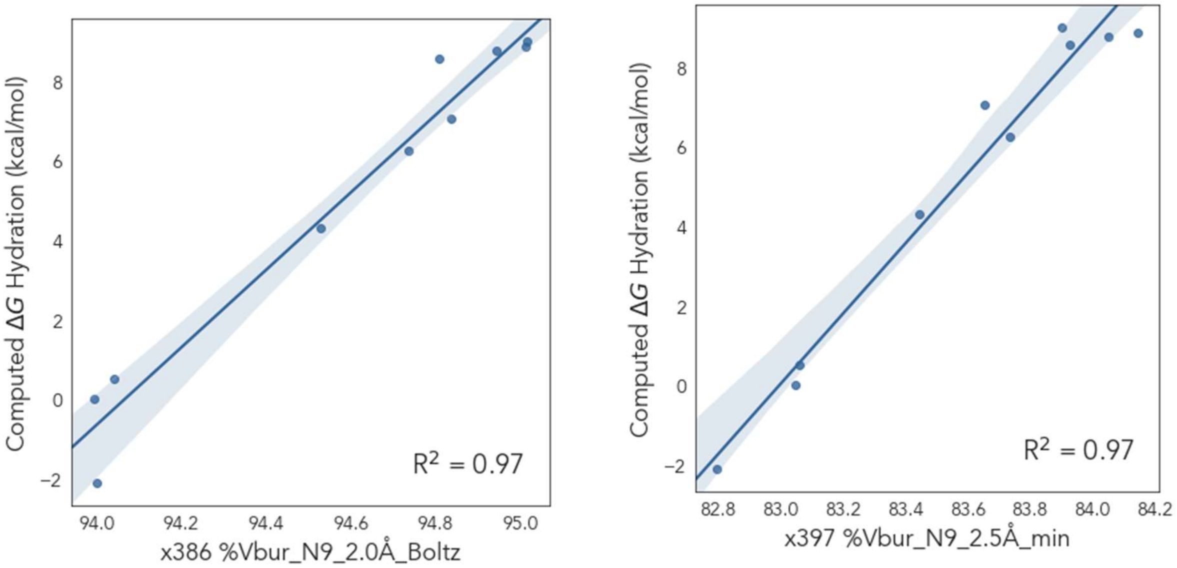

Figure S5. Correlation between ΔG of ketone hydration and computed Vbur(N9) at 2.0 Å (left) and 2.5 Å (right).

XYZ Coordinates for LEC Analog Conformers

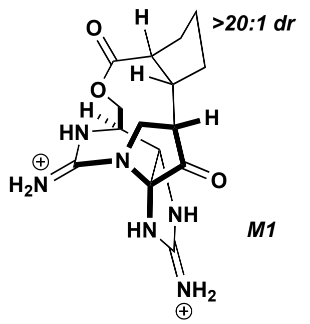

M1 – Ketone

computed as single diastereomer

Energy: -714616.4630019

C	-1.49169	1.59171	-1.16097
N	-1.79228	2.12376	0.16026
C	-1.33535	1.52198	1.26120
C	-0.05453	1.86781	-1.58992
H	-2.14664	2.09416	-1.88339
N	-1.20438	2.19304	2.39266
H	-1.34669	3.19778	2.41100
H	-1.05457	1.70813	3.27095
H	-2.09815	3.08920	0.23850
N	-1.00618	0.21540	1.21939
C	0.04143	-1.35943	-0.15298
C	0.87877	-1.26853	1.09923
C	0.07926	-0.35082	2.04224
O	0.29781	-1.91413	-1.18835
H	0.69585	0.46697	2.43487
H	-0.33619	-0.90808	2.89186
H	0.16109	1.37303	-2.54620
H	0.11028	2.94900	-1.69627
O	0.80371	1.37750	-0.56365
C	2.03625	0.93505	-0.84231
O	2.48031	0.88908	-1.96456
C	-1.81909	0.10605	-1.17690
C	-1.30136	-0.62612	0.07737
N	-2.37816	-1.56632	0.33420
C	-3.49630	-1.19842	-0.31349
N	-3.24401	-0.17327	-1.12708
H	-1.36955	-0.36301	-2.06479
N	-4.66556	-1.77866	-0.16246
H	-2.38846	-2.17287	1.15001
H	-3.88234	0.12006	-1.86048
H	-5.46460	-1.46935	-0.70604
H	-4.77982	-2.54189	0.49617
H	0.87197	-2.29747	1.49735
C	2.74396	0.52780	0.42900
C	2.36244	-0.89276	0.92684
C	3.18822	-1.81937	0.02529
C	4.51201	-1.06576	-0.20981
C	4.25885	0.39477	0.23106
H	2.48477	1.28577	1.18164
H	2.78504	-0.94736	1.94326
H	3.32777	-2.80718	0.48767
H	2.66311	-1.97710	-0.92619
H	4.80687	-1.11916	-1.26711
H	5.33253	-1.50645	0.37397
H	4.64483	1.13257	-0.48506
H	4.74490	0.59229	1.19685

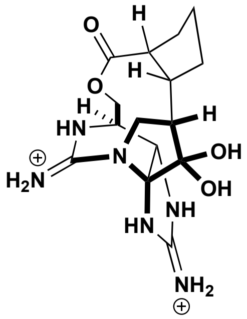

M1 – Hydrate

computed as single diastereomer

Energy: -762588.4831617

C	-1.44908	1.74916	-1.07471
N	-1.78479	2.20893	0.26320
C	-1.29485	1.56252	1.32576
C	-0.02056	2.11640	-1.46864
H	-2.10891	2.26818	-1.78110
N	-1.16422	2.18078	2.48995
H	-1.33483	3.17876	2.55749
H	-1.00072	1.65917	3.34376
H	-2.08485	3.17081	0.38885
N	-0.93316	0.27788	1.19923
C	0.00016	-1.45511	-0.15872
C	0.86183	-1.27790	1.12435
C	0.04602	-0.37398	2.06188
O	-0.33075	-2.80425	-0.25503
H	0.66974	0.38490	2.55125
H	-0.46015	-0.95812	2.84408
H	0.24440	1.65067	-2.42654
H	0.06192	3.20775	-1.56990
O	0.86427	1.69208	-0.44025
C	2.04194	1.14279	-0.75106
O	2.49885	1.16206	-1.87146
C	-1.76704	0.25925	-1.17195
C	-1.28693	-0.55307	0.06096
N	-2.45171	-1.36826	0.34992
C	-3.52309	-0.97973	-0.34757
N	-3.20106	0.01401	-1.17333
H	-1.31136	-0.15091	-2.08072
N	-4.72258	-1.51073	-0.23084
H	-2.46496	-2.09049	1.06259
H	-3.81011	0.33653	-1.91806
H	-5.48695	-1.18204	-0.81067
H	-4.89007	-2.25630	0.43606
H	0.91531	-2.27745	1.57377
C	2.72427	0.59850	0.48372
C	2.32237	-0.83143	0.96680
C	3.23628	-1.81420	0.19376
C	4.36469	-0.95516	-0.40898
C	4.23038	0.41889	0.26001
H	2.50525	1.32253	1.28201
H	2.69661	-0.83261	2.00232
H	3.62236	-2.57198	0.89088
H	2.70344	-2.38344	-0.58325
H	5.35978	-1.39515	-0.25368
H	4.21999	-0.85104	-1.49401
H	4.67208	1.23319	-0.33083
H	4.72539	0.41418	1.24314
O	0.58752	-1.00903	-1.33768
H	-0.89428	-2.92714	-1.03292
H	1.37611	-1.53647	-1.51956

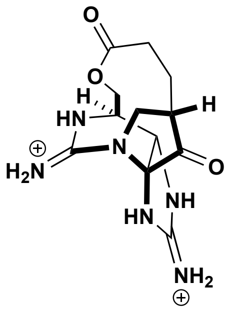

M2

M2 – Ketone

Energy: -641373.4526724

C	-0.42665	-0.46842	-1.94749
N	-0.66529	0.96489	-2.02528
C	-0.52737	1.78424	-0.98031
C	1.04459	-0.83644	-2.05970
H	-0.93633	-0.93283	-2.80119
N	-0.36052	3.08343	-1.17472
H	-0.24178	3.44323	-2.11612

H	-0.48517	3.74715	-0.41840
H	-0.69941	1.38825	-2.94817
N	-0.58585	1.28959	0.27028
C	0.35007	-0.48080	1.46585
C	1.05501	0.78825	1.89515
C	0.08457	1.88695	1.43893
O	0.61273	-1.61322	1.77047
H	0.60018	2.81266	1.16373
H	-0.66428	2.12230	2.20741
H	1.15808	-1.92818	-2.09249
H	1.48580	-0.40108	-2.96675
O	1.70519	-0.30484	-0.91637
C	2.74146	-0.95394	-0.37068
O	3.21086	-1.95995	-0.84566
C	-1.04522	-1.02146	-0.67024
C	-0.85121	-0.10959	0.55612
N	-2.10717	-0.29874	1.25919
C	-3.04028	-0.80650	0.43702
N	-2.49368	-1.13276	-0.73452
H	-0.60090	-2.00271	-0.44223
N	-4.30534	-0.96241	0.75400
H	-2.34037	0.20758	2.10957
H	-2.95610	-1.74423	-1.40115
H	-4.95195	-1.37813	0.09165
H	-4.65086	-0.65565	1.65755
H	1.13217	0.74236	2.99047
C	3.23238	-0.27724	0.88336
C	2.47435	0.97930	1.29098
H	4.28804	-0.02648	0.69676
H	2.39528	1.63650	0.41375
H	3.23956	-1.04113	1.67607
H	3.07261	1.52040	2.03548

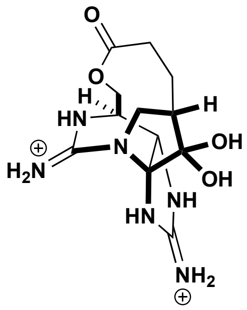

M2 – Hydrate

Energy: -689347.1564024

C	0.49414	-1.84819	-1.01795
N	0.66046	-2.32311	0.34690
C	0.30648	-1.54010	1.37286
C	-0.96829	-1.88219	-1.46219
H	1.03804	-2.53409	-1.67935
N	-0.00914	-2.07081	2.54431
H	-0.11554	-3.07570	2.63617
H	-0.07966	-1.49861	3.37824
H	0.70885	-3.32373	0.51248
N	0.28225	-0.21099	1.20148
C	-0.10454	1.67081	-0.21323
C	-1.03878	1.75213	1.02896
C	-0.49708	0.71427	2.02222
O	0.58350	2.87870	-0.30048
H	-1.29522	0.17214	2.54502
H	0.14710	1.18423	2.77960
H	-1.09668	-1.36345	-2.42145
H	-1.29438	-2.92564	-1.57431
O	-1.75051	-1.27228	-0.44751
C	-2.74317	-0.42940	-0.74218
O	-3.19728	-0.29696	-1.85498
C	1.15992	-0.47952	-1.14491
C	0.88327	0.46081	0.06186
N	2.21616	0.93769	0.37942
C	3.16258	0.29596	-0.31089

N	2.61033	-0.58731	-1.13904
H	0.82082	0.00236	-2.06897
N	4.45581	0.51192	-0.18402
H	2.39974	1.66795	1.05980
H	3.12858	-1.06748	-1.86743
H	5.11876	0.00554	-0.76087
H	4.79859	1.19436	0.48300
H	-0.87509	2.75167	1.44837
C	-3.20574	0.29295	0.49332
C	-2.54751	1.66104	0.74014
H	-3.04901	-0.36685	1.35782
H	-3.05710	2.08323	1.61858
H	-4.28604	0.45950	0.38625
H	-2.79321	2.33901	-0.09196
O	-0.75323	1.38525	-1.40867
H	1.18654	2.83203	-1.05689
H	-1.38355	2.09264	-1.60241

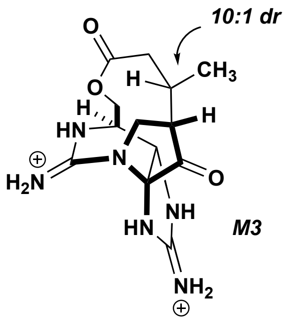

M3 – Ketone

computed as single diastereomer

Energy: -666040.0893259

C	-0.96806	1.77091	0.93571
N	-1.03448	0.85002	2.06013
C	-0.63439	-0.42170	1.98384
C	0.41219	2.38130	0.74535
H	-1.65506	2.59958	1.14836
N	-0.33278	-1.08751	3.08773
H	-0.31066	-0.60639	3.98114
H	-0.23836	-2.09675	3.08203
H	-1.14582	1.24751	2.98821
N	-0.56939	-1.03201	0.78566
C	0.32386	-0.56421	-1.31755
C	1.22864	-1.57282	-0.64644
C	0.33590	-2.14871	0.46141
O	0.49546	0.00744	-2.36092
H	0.90930	-2.45994	1.34104
H	-0.25607	-3.00688	0.11494
H	0.38589	3.12825	-0.05896
H	0.75330	2.86152	1.67267
O	1.30675	1.32485	0.41230
C	2.30611	1.52924	-0.45595
O	2.54931	2.61015	-0.93603
C	-1.45090	1.07136	-0.32735
C	-0.95342	-0.38047	-0.45536
N	-2.09575	-1.02267	-1.07835
C	-3.20475	-0.28284	-0.91061
N	-2.89395	0.89846	-0.37643
H	-1.10702	1.63110	-1.21124
N	-4.41458	-0.67784	-1.23548
H	-2.12689	-2.02046	-1.27188
H	-3.53322	1.68792	-0.37922
H	-5.20717	-0.05539	-1.11671
H	-4.56629	-1.60738	-1.61282
H	1.46997	-2.32442	-1.41263
C	3.07578	0.26603	-0.74256
C	2.56173	-1.00409	-0.06608
H	4.10646	0.46415	-0.40702
H	2.36449	-0.74837	0.98554
H	3.13034	0.16219	-1.83760
C	3.62143	-2.10152	-0.09319
H	4.52901	-1.78043	0.43889

H	3.24834	-3.01649	0.39123
H	3.89918	-2.35277	-1.12893

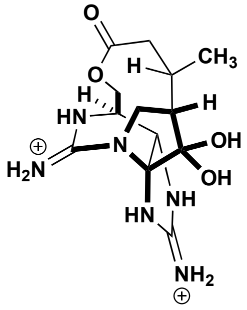

M3 – Hydrate

computed as single diastereomer

Energy: -714010.7913017

C	-0.88001	2.10455	0.10880
N	-0.94948	1.78709	1.52616
C	-0.54212	0.61625	2.02652
C	0.48453	2.61922	-0.32284
H	-1.58408	2.92681	-0.07338
N	-0.22377	0.52570	3.31110
H	-0.18466	1.36325	3.88239
H	-0.13349	-0.37439	3.76815
H	-1.04021	2.56182	2.17682
N	-0.48398	-0.46129	1.23206
C	0.17688	-1.29852	-0.90063
C	1.22100	-1.66356	0.19339
C	0.34937	-1.64762	1.45308
O	0.60179	-0.53767	-1.97670
H	0.93594	-1.56059	2.37407
H	-0.29162	-2.53724	1.53397
H	0.46177	2.89921	-1.38426
H	0.75911	3.50070	0.27298
O	1.44212	1.59910	-0.09603
C	2.29890	1.24345	-1.06015
O	2.45017	1.87658	-2.07946
C	-1.36523	0.90986	-0.71068
C	-0.96983	-0.48197	-0.14119
N	-2.23125	-1.19729	-0.22452
C	-3.25655	-0.39156	-0.51502
N	-2.81916	0.84533	-0.72895
H	-0.98129	0.99562	-1.73471
N	-4.51380	-0.77686	-0.58142
H	-2.33131	-2.17284	0.03710
H	-3.39389	1.57904	-1.13058
H	-5.23849	-0.11808	-0.84518
H	-4.76411	-1.74268	-0.39933
H	1.53597	-2.68789	-0.04425
C	3.08138	0.01759	-0.68019
C	2.49854	-0.81689	0.46109
H	4.06799	0.39227	-0.35764
H	2.25750	-0.10077	1.25984
H	3.26944	-0.56300	-1.59504
C	3.57267	-1.75749	1.01090
H	3.18468	-2.35570	1.84952
H	3.92036	-2.45441	0.23152
H	4.44207	-1.19017	1.37525
O	-0.31801	-2.52754	-1.34462
H	1.23198	-1.06209	-2.49012
H	-0.99934	-2.36113	-2.01207

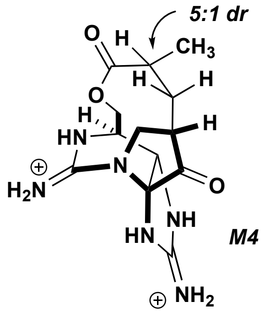

M4

M4 – Ketone

computed as single diastereomer

Energy: -666040.7151788

C	-0.90368	1.74360	-0.96586
N	-1.08874	2.16689	0.41412
C	-0.74020	1.37048	1.42709
C	0.55709	1.79038	-1.40167
H	-1.45723	2.43866	-1.60892
N	-0.48242	1.87520	2.62167
H	-0.42261	2.88018	2.75109
H	-0.40675	1.27699	3.43733
H	-1.22481	3.15449	0.60892
N	-0.64999	0.03839	1.23159
C	0.09997	-1.50228	-0.35458
C	0.94750	-1.71750	0.87130
C	0.35838	-0.78073	1.93740
O	0.28916	-1.93848	-1.45843
H	1.11762	-0.11639	2.36877
H	-0.11041	-1.34298	2.75487
H	0.66321	1.39588	-2.42125
H	0.93222	2.82287	-1.37262
O	1.30251	1.00238	-0.47667
C	2.49233	0.49210	-0.81634
O	2.98213	0.64936	-1.90847
C	-1.50168	0.35533	-1.13461
C	-1.09906	-0.59835	0.00629
N	-2.31507	-1.36926	0.18590
C	-3.36369	-0.73887	-0.36935
N	-2.95214	0.32809	-1.05580
H	-1.16257	-0.08093	-2.08613
N	-4.61239	-1.13126	-0.25263
H	-2.41636	-2.06347	0.92163
H	-3.54136	0.79985	-1.73557
H	-5.35588	-0.62225	-0.71958
H	-4.84538	-1.94071	0.31318
H	0.74091	-2.76317	1.15483
C	3.10375	-0.26357	0.34572
C	2.45563	-1.64272	0.58868
H	2.91897	0.36412	1.23167
H	2.98025	-2.08907	1.44529
C	4.60646	-0.42226	0.14513
H	2.65830	-2.29278	-0.27722
H	5.05368	-0.90908	1.02318
H	4.82194	-1.04150	-0.73823
H	5.09418	0.55329	0.00807

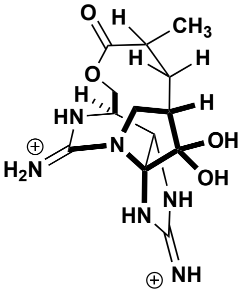

M4 – Hydrate

computed as single diastereomer

Energy: -714013.6573257

C	0.81420	-1.84433	-1.01262
N	0.95832	-2.31185	0.35683
C	0.55361	-1.53807	1.37069
C	-0.63497	-1.92108	-1.49307
H	1.39442	-2.51535	-1.65814
N	0.22221	-2.07762	2.53373
H	0.14559	-3.08529	2.62393
H	0.11206	-1.50832	3.36519
H	1.03309	-3.31015	0.52662
N	0.49352	-0.21068	1.19530
C	0.06809	1.64474	-0.24523
C	-0.89990	1.69517	0.97171
C	-0.33701	0.69261	1.98935
O	0.70912	2.87914	-0.32290
H	-1.12325	0.12803	2.50611

H	0.27486	1.19775	2.75081
H	-0.75534	-1.40620	-2.45525
H	-0.92724	-2.97371	-1.61223
O	-1.46121	-1.33608	-0.49759
C	-2.48025	-0.53869	-0.82034
O	-2.90626	-0.42507	-1.94641
C	1.44377	-0.45764	-1.12446
C	1.09668	0.47802	0.06627
N	2.40023	1.01152	0.41569
C	3.38974	0.39504	-0.23626
N	2.89586	-0.52119	-1.06653
H	1.12314	0.00999	-2.06248
N	4.66971	0.66110	-0.07727
H	2.53582	1.74997	1.09832
H	3.45290	-0.97841	-1.78099
H	5.36741	0.16681	-0.62285
H	4.96903	1.36212	0.59164
H	-0.78897	2.70456	1.38465
C	-3.02691	0.15420	0.40846
C	-2.39712	1.54273	0.65293
H	-2.78272	-0.49432	1.26282
H	-2.93499	1.95618	1.51944
C	-4.54369	0.28902	0.29676
H	-2.65397	2.20498	-0.19007
H	-4.95261	0.73985	1.21218
H	-4.81685	0.93020	-0.55453
H	-5.02088	-0.69149	0.15447
O	-0.53550	1.32552	-1.45532
H	1.33457	2.85240	-1.06200
H	-1.19153	2.00298	-1.66925

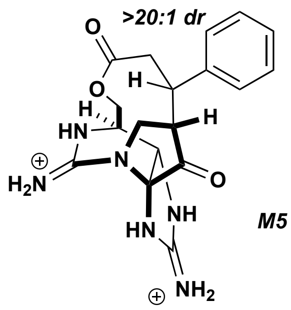

M5

M5 – Ketone

computed as single diastereomer

Energy: -786353.0985479

C	-2.59059	1.27041	1.02361
N	-2.25205	0.33272	2.08257
C	-1.35620	-0.64519	1.93620
C	-1.61065	2.42815	0.91082
H	-3.56712	1.70594	1.27082
N	-0.81802	-1.21362	3.00468
H	-1.00068	-0.83192	3.92709
H	-0.32055	-2.09507	2.93757
H	-2.54655	0.55835	3.02827
N	-1.02297	-1.06885	0.70165
C	-0.42317	-0.10695	-1.33688
C	0.83156	-0.66912	-0.70356
C	0.28277	-1.64975	0.34154
O	-0.51475	0.55357	-2.33637
H	0.93341	-1.72937	1.21866
H	0.13512	-2.65688	-0.07118
H	-1.96214	3.14698	0.15905
H	-1.50305	2.93964	1.87705
O	-0.35065	1.88725	0.52746
C	0.46698	2.57742	-0.27659
O	0.22804	3.69846	-0.65472
C	-2.72888	0.52156	-0.29491
C	-1.65203	-0.55905	-0.50549
N	-2.40541	-1.58243	-1.20834

C	-3.72485	-1.41158	-1.01646
N	-3.95344	-0.25736	-0.38962
H	-2.66756	1.23558	-1.13107
N	-4.64772	-2.26346	-1.40029
H	-2.00055	-2.48095	-1.45882
H	-4.87197	0.17622	-0.36094
H	-5.63066	-2.05161	-1.26235
H	-4.38751	-3.13156	-1.85679
H	1.37330	-1.18392	-1.50957
C	1.70334	1.79400	-0.63744
C	1.78099	0.38459	-0.04371
H	2.55359	2.39148	-0.27350
H	1.47090	0.46927	1.00638
H	1.77637	1.79661	-1.73564
C	5.80919	-1.19401	-0.04579
C	5.24576	-0.71221	-1.22959
C	3.95064	-0.19893	-1.23052
C	3.19932	-0.15309	-0.04840
C	3.77263	-0.63668	1.13161
C	5.06893	-1.15597	1.13444
H	6.82403	-1.59803	-0.04575
H	5.81948	-0.73785	-2.15888
H	3.51678	0.16801	-2.16470
H	3.19631	-0.60498	2.06023
H	5.50071	-1.53072	2.06528

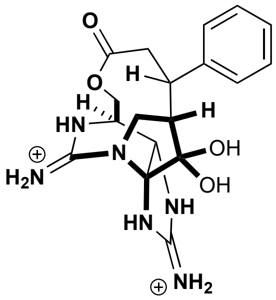

M5 – Hydrate

computed as single diastereomer

Energy: -834324.8389891

C	-2.48663	1.18453	-1.23287
N	-3.13876	1.44818	0.04042
C	-2.51690	1.12642	1.17884
C	-1.35709	2.17205	-1.51555
H	-3.23184	1.32529	-2.02569
N	-2.81369	1.74596	2.31093
H	-3.42795	2.55346	2.30510
H	-2.50980	1.37528	3.20478
H	-3.87475	2.14643	0.08032
N	-1.57954	0.16751	1.16159
C	0.17040	-0.98999	0.01098
C	0.72283	-0.40161	1.33993
C	-0.49414	0.09245	2.13435
O	0.48446	-2.34640	-0.00435
H	-0.33620	1.07993	2.58611
H	-0.74808	-0.61028	2.94080
H	-0.80900	1.88014	-2.42094
H	-1.77321	3.17899	-1.65961
O	-0.49136	2.19501	-0.38812
C	0.83001	2.27988	-0.54609
O	1.34696	2.55926	-1.60415
C	-2.05560	-0.28059	-1.26888
C	-1.39885	-0.75910	0.05680
N	-2.09666	-2.00618	0.30044
C	-3.12562	-2.18254	-0.53227
N	-3.19377	-1.17652	-1.40159
H	-1.35583	-0.43040	-2.09921
N	-3.94885	-3.20970	-0.49019
H	-1.85168	-2.64411	1.05059
H	-3.79317	-1.18616	-2.22026
H	-4.69691	-3.29669	-1.16937
H	-3.83696	-3.92564	0.21924

H	1.13467	-1.26015	1.87984
C	1.54296	2.05865	0.76604
C	1.88533	0.61897	1.22281
H	0.91162	2.51564	1.54031
H	2.19789	0.76605	2.26932
H	2.47730	2.63427	0.73152
C	5.33398	-1.26835	-0.64224
C	4.79235	-0.11745	-1.20664
C	3.68639	0.50581	-0.62131
C	3.09603	-0.01472	0.53705
C	3.66022	-1.17190	1.09902
C	4.76322	-1.79169	0.51986
H	6.19794	-1.75506	-1.09999
H	5.22991	0.30858	-2.11229
H	3.28116	1.40307	-1.09068
H	3.23723	-1.59202	2.01494
H	5.18289	-2.68845	0.98140
O	0.63213	-0.36975	-1.14488
H	0.13256	-2.73113	-0.82068
H	1.59039	-0.50180	-1.20382

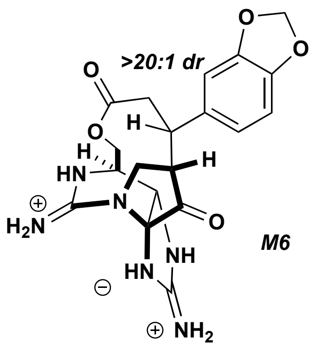

M6 – Ketone

computed as single diastereomer

Energy: -904671.0897564

C	-3.45862	1.23431	0.73851
N	-3.10059	0.49518	1.93931
C	-2.11518	-0.40412	1.98145
C	-2.58158	2.45477	0.50484
H	-4.48401	1.60109	0.87270
N	-1.58652	-0.75154	3.14511
H	-1.86253	-0.26498	3.99169
H	-1.01917	-1.58639	3.23587
H	-3.46640	0.83108	2.82577
N	-1.68186	-0.97729	0.84217
C	-1.03183	-0.26027	-1.27957
C	0.21923	-0.61374	-0.50510
C	-0.31923	-1.49747	0.62749
O	-1.11142	0.24460	-2.36699
H	0.28463	-1.42458	1.53843
H	-0.37622	-2.55492	0.33642
H	-2.94073	3.01519	-0.36836
H	-2.58923	3.11200	1.38500
O	-1.25490	1.98877	0.28410
C	-0.44676	2.61292	-0.58107
O	-0.75435	3.62763	-1.15768
C	-3.44617	0.29802	-0.46229
C	-2.27252	-0.69931	-0.45648
N	-2.89173	-1.87531	-1.03937
C	-4.23050	-1.79776	-0.95281
N	-4.59455	-0.59318	-0.51227
H	-3.38691	0.89129	-1.38810
N	-5.05208	-2.77297	-1.26738
H	-2.39942	-2.75871	-1.14466
H	-5.54502	-0.24441	-0.59607
H	-6.05597	-2.63578	-1.20944
H	-4.68959	-3.67145	-1.56947
H	0.86343	-1.17138	-1.19949
C	0.87594	1.90462	-0.73025
C	1.01629	0.60428	0.06722
H	1.63857	2.62302	-0.39121
H	0.60820	0.80263	1.06685

H	1.04747	1.76229	-1.80810
C	3.30495	0.04516	-0.87192
C	2.46889	0.21300	0.25486
C	2.97461	0.00658	1.53835
C	4.31364	-0.36761	1.76190
H	2.93462	0.18965	-1.88878
H	2.31364	0.14342	2.39758
H	4.70253	-0.53104	2.76756
C	5.10960	-0.51712	0.64845
C	4.61280	-0.31448	-0.63933
O	5.60470	-0.56130	-1.54421
C	6.80199	-0.69003	-0.78693
O	6.41993	-0.89428	0.56763
H	7.37519	-1.55497	-1.14489
H	7.39316	0.23897	-0.86920

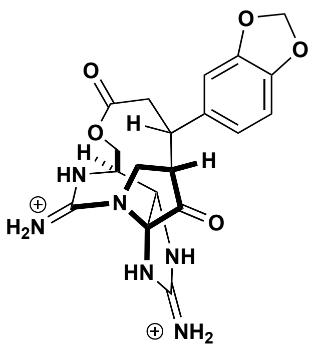

M6 – Hydrate

computed as single diastereomer

Energy: -952642.6505291

C	3.30689	-0.81648	-1.22672
N	3.99401	-0.93600	0.04938
C	3.31879	-0.73001	1.18444
C	2.39080	-2.00743	-1.49878
H	4.06718	-0.81901	-2.01814
N	3.72622	-1.27730	2.31928
H	4.48763	-1.94790	2.31569
H	3.35857	-0.96748	3.21201
H	4.85047	-1.47937	0.09606
N	2.21809	0.03602	1.16031
C	0.27726	0.80062	-0.01142
C	-0.15203	0.14136	1.33000
C	1.13689	-0.08037	2.13431
O	-0.30809	2.06324	-0.05936
H	1.17357	-1.06723	2.61303
H	1.24519	0.68032	2.92066
H	1.79006	-1.83404	-2.40122
H	2.99567	-2.91379	-1.64188
O	1.55374	-2.19396	-0.36496
C	0.27407	-2.53693	-0.51385
O	-0.18142	-2.92708	-1.56521
C	2.60261	0.53730	-1.27686
C	1.86034	0.89320	0.04152
N	2.29404	2.25806	0.26731
C	3.26982	2.62363	-0.56804
N	3.54723	1.63464	-1.41514
H	1.89097	0.54383	-2.11069
N	3.86764	3.79703	-0.55013
H	1.91178	2.85156	0.99613
H	4.12699	1.75795	-2.23881
H	4.58304	4.01840	-1.23437
H	3.60413	4.49568	0.13624
H	-0.72974	0.91171	1.85092
C	-0.46437	-2.44318	0.79921
C	-1.08581	-1.09474	1.24097
H	0.24815	-2.75586	1.57469
H	-1.34852	-1.28514	2.29401
H	-1.26628	-3.19291	0.77654
C	-3.19807	0.27606	1.17114
C	-2.40907	-0.73204	0.56537
C	-2.86843	-1.34918	-0.60063
C	-4.09043	-0.99646	-1.20861

H	-2.89197	0.76792	2.09606
H	-2.27170	-2.12778	-1.07663
H	-4.43531	-1.49224	-2.11655
O	-0.03913	0.07313	-1.15372
H	-0.05225	2.48707	-0.89206
H	-1.00341	-0.00167	-1.21778
C	-4.82699	-0.00598	-0.60313
C	-4.38563	0.61288	0.56625
O	-5.32242	1.51851	0.97227
C	-6.25447	1.62546	-0.09732
O	-6.04429	0.50572	-0.94891
H	-7.27756	1.60230	0.29952
H	-6.06763	2.55780	-0.65840

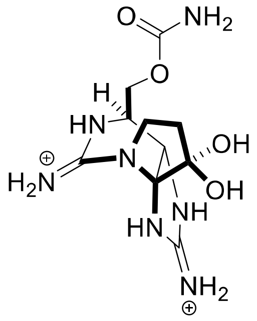

(+)-STX

Energy: -675524.0900058

C	-0.63622	-1.37319	-0.61820
N	-0.92744	-1.25405	0.80484
C	-0.68965	-0.09382	1.43263
C	-1.60489	-0.55955	-1.48010
H	-0.74998	-2.43333	-0.87551
N	-1.37835	0.25402	2.50593
H	-2.22463	-0.25106	2.74798
H	-1.12361	1.06682	3.05510
H	-1.66405	-1.84363	1.18173
N	0.27950	0.69188	0.94839
C	1.65107	1.53090	-0.74036
C	1.58077	2.56782	0.37548
C	0.38456	2.13537	1.21676
O	2.91497	1.50360	-1.30927
O	0.65063	1.71016	-1.69555
H	2.51041	2.52925	0.95976
H	1.46699	3.58116	-0.02992
H	-0.54950	2.62203	0.90109
H	0.54821	2.33432	2.28452
H	-1.43719	0.51733	-1.35963
H	-1.45692	-0.82579	-2.53446
O	-2.94019	-0.89101	-1.14274
C	-3.62618	-0.02715	-0.36011
O	-3.19429	1.06017	-0.01335
H	-5.46520	0.05460	0.49629
N	-4.82205	-0.52811	-0.02469
H	-5.13728	-1.41406	-0.40041
C	0.81075	-0.96677	-0.89290
C	1.28126	0.21134	0.00860
N	2.43186	-0.35369	0.68798
C	2.68227	-1.60725	0.29527
N	1.75702	-2.01743	-0.56514
H	0.91100	-0.68730	-1.95075
N	3.70146	-2.32945	0.71215
H	3.09466	0.20708	1.21489
H	1.74131	-2.94024	-0.98595
H	3.82956	-3.27657	0.37312
H	4.35591	-1.95222	1.38858
H	2.89830	0.88525	-2.05415
H	0.70657	2.61458	-2.03363

(+)-STX Ketone

Energy: -627544.9373161

C	0.10694	-1.64760	-0.99852
N	-0.02212	-2.02813	0.40190

C	-0.28560	-1.12104	1.34479
C	-1.21836	-1.39909	-1.71055
H	0.56523	-2.50051	-1.51491
N	-0.81651	-1.47128	2.50246
H	-1.19155	-2.40606	2.62660
H	-0.87267	-0.81832	3.27607
H	-0.22692	-3.00050	0.61282
N	0.01782	0.16476	1.09376
C	0.27426	1.92393	-0.39209
C	-0.48192	2.46437	0.78953
C	-0.58896	1.31195	1.79079
O	0.41912	2.42063	-1.47573
H	0.08606	3.31825	1.18879
H	-1.45875	2.84035	0.45908
H	-1.63320	1.07188	2.02947
H	-0.05256	1.52487	2.72623
H	-1.02779	-1.38053	-2.79110
H	-1.92269	-2.20948	-1.48392
O	-1.79454	-0.13584	-1.39727
C	-2.80363	-0.09080	-0.49825
O	-3.12485	-1.04045	0.19530
H	-4.10314	1.30643	0.20028
N	-3.38002	1.11775	-0.48248
H	-3.04543	1.86034	-1.08428
C	1.06693	-0.46520	-1.12150
C	0.89661	0.56740	0.01753
N	2.26799	0.77930	0.45483
C	3.10515	-0.10959	-0.09913
N	2.45537	-0.86684	-0.97672
H	0.91443	0.03931	-2.08716
N	4.38547	-0.20270	0.18703
H	2.51265	1.36293	1.25002
H	2.90783	-1.53278	-1.59452
H	4.96539	-0.89474	-0.27555
H	4.80069	0.40785	0.88244

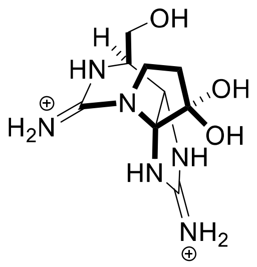

dc-STX

Energy: -569641.2639026

C	-1.36546	-1.26213	-0.58489
N	-1.66011	-1.04667	0.83152
C	-1.38582	0.15950	1.35492
C	-2.29610	-0.45059	-1.47843
H	-1.54264	-2.32630	-0.78853
N	-2.13079	0.67456	2.31864
H	-2.98279	0.20445	2.60607
H	-1.83401	1.49441	2.83644
H	-2.49921	-1.50220	1.18110
N	-0.33182	0.82570	0.87152
C	1.20533	1.42698	-0.77165
C	1.19530	2.52057	0.29100
C	-0.08244	2.26032	1.08240
O	2.48442	1.23502	-1.27154
O	0.27501	1.66314	-1.78410
H	2.08540	2.41299	0.92592
H	1.21246	3.51855	-0.16474
H	-0.93819	2.83309	0.69687
H	0.04624	2.48837	2.14947
H	-2.12392	0.62845	-1.31979
H	-2.04642	-0.67473	-2.53081
O	-3.61623	-0.81960	-1.15864

C	0.10670	-0.96616	-0.86139
C	0.65792	0.19499	0.01489
N	1.69966	-0.45690	0.78673
C	1.87477	-1.72965	0.41266
N	0.97706	-2.07226	-0.50363
H	0.23014	-0.71981	-1.92403
N	2.80476	-2.52645	0.89698
H	2.39066	0.05862	1.32383
H	0.88142	-3.00815	-0.88276
H	2.88059	-3.48300	0.56880
H	3.44994	-2.19153	1.60391
H	2.43559	0.58823	-1.99047
H	0.45062	2.53534	-2.16397
H	-4.21407	-0.19407	-1.58240

dc-STX Ketone

Energy: -521662.9158589

C	-0.43024	-1.60820	-1.06907
N	-0.77084	-1.95343	0.30521
C	-1.04215	-1.00526	1.20454
C	-1.64352	-1.17404	-1.87749
H	-0.02213	-2.50948	-1.54328
N	-1.79485	-1.27799	2.25907
H	-2.25511	-2.17928	2.33232
H	-1.83179	-0.65162	3.05548
H	-1.12807	-2.88664	0.48639
N	-0.53167	0.22861	1.04130
C	-0.14716	1.87521	-0.57458
C	-1.13021	2.44014	0.40963
C	-1.17793	1.44448	1.56828
O	0.20185	2.33669	-1.62754
H	-0.80686	3.44557	0.71271
H	-2.10068	2.53754	-0.09729
H	-2.20633	1.21242	1.86785
H	-0.63150	1.81193	2.44780
H	-2.38461	-1.99159	-1.86748
H	-1.32333	-1.01784	-2.92161
O	-2.16749	0.00423	-1.31004
C	0.66270	-0.54781	-1.05599
C	0.42193	0.55193	-0.00241
N	1.76316	0.76661	0.51848
C	2.57755	-0.24550	0.17925
N	1.96057	-1.07263	-0.66124
H	0.73075	-0.07353	-2.04682
N	3.80962	-0.38474	0.61713
H	1.95392	1.38866	1.29947
H	2.44427	-1.79189	-1.18994
H	4.38382	-1.15771	0.29791
H	4.19812	0.28326	1.27418
H	-2.89301	0.32210	-1.85971

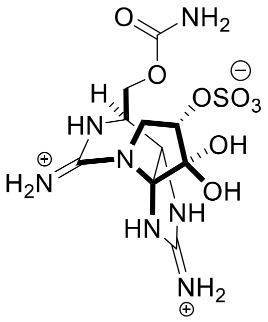

GTX

Energy: -1113935.5850828

C	-2.68979	1.06083	0.02900
N	-2.18282	2.23679	-0.66114
C	-1.04951	2.17018	-1.37504
C	-3.32685	0.08239	-0.96458
H	-3.48451	1.39868	0.70607
N	-0.84293	2.98440	-2.39776
H	-1.58698	3.59632	-2.71654
H	0.07264	3.07600	-2.82424

H	-2.86281	2.93674	-0.94471
N	-0.13145	1.26625	-1.02366
C	0.48935	-0.81634	-0.20377
C	1.63783	-0.29692	-1.10129
C	0.99134	0.84900	-1.87367
O	0.85014	-1.53160	0.91479
O	-0.32743	-1.52504	-1.07580
O	2.69957	0.21133	-0.30918
H	1.99089	-1.08829	-1.77626
H	0.61459	0.51445	-2.84935
H	1.71594	1.66132	-2.02144
H	-3.95407	0.65252	-1.66079
H	-2.56760	-0.46774	-1.53650
O	-4.20257	-0.80974	-0.29072
C	-3.81342	-2.06423	-0.01066
O	-2.68349	-2.49582	-0.21523
H	-4.63216	-3.74021	0.78640
N	-4.80647	-2.77820	0.52320
H	-5.72863	-2.37739	0.64392
C	-1.58491	0.43632	0.88274
C	-0.17865	0.52762	0.22928
N	0.60368	1.19059	1.25785
C	-0.12451	1.46800	2.34099
N	-1.39708	1.13609	2.14163
H	-1.83253	-0.61391	1.08585
N	0.35540	2.00685	3.44284
H	1.62063	1.18790	1.23392
H	-2.11850	1.20790	2.85087
H	-0.25101	2.17970	4.23699
H	1.33786	2.24983	3.50677
H	1.75520	-1.89334	0.79407
H	-1.14192	-1.85347	-0.63582
S	4.01899	-0.77805	-0.07785
O	4.75599	-0.02940	0.93318
O	3.43544	-2.05567	0.39251
O	4.65429	-0.88641	-1.39107

GTX Ketone

Energy: -1065954.4688595

C	2.90880	0.83643	0.02188
N	2.55989	0.63686	1.42410
C	1.53782	-0.17098	1.73800
C	3.68854	-0.36001	-0.53750
H	3.61055	1.67901	-0.01950
N	1.50500	-0.83187	2.87887
H	2.32155	-0.84263	3.48223
H	0.66099	-1.29362	3.20142
H	3.31341	0.70682	2.10330
N	0.53139	-0.26508	0.85082
C	-0.62383	0.05241	-1.13981
C	-1.13768	-1.23754	-0.49582
C	-0.56548	-1.23224	0.92888
O	-0.91550	0.48681	-2.21718
O	-2.52463	-1.42018	-0.62327
H	-0.68737	-2.05544	-1.07735
H	-0.17262	-2.21797	1.20859
H	-1.31719	-0.92498	1.67013
H	3.77976	-0.27348	-1.62861
H	4.69205	-0.34827	-0.09618
O	3.15361	-1.62349	-0.16955
C	2.17001	-2.14767	-0.92500
O	1.65864	-1.55002	-1.86101
H	2.29221	-3.79891	0.28620
N	1.83154	-3.37248	-0.50863
H	1.07971	-3.86000	-0.98056
C	1.67030	1.27115	-0.78182
C	0.34678	0.74883	-0.16219
N	-0.27954	1.95715	0.35230

C	0.36029	3.04858	-0.09656
N	1.47297	2.71004	-0.73880
H	1.77023	0.93105	-1.81855
N	-0.06176	4.28075	0.08780
H	-1.28322	1.93745	0.55233
H	2.17182	3.37749	-1.04865
H	0.46988	5.06535	-0.27371
H	-0.92524	4.45573	0.59026
S	-3.56752	-0.39026	0.17946
O	-3.76956	-1.00281	1.49499
O	-2.83928	0.89421	0.23061
O	-4.73151	-0.40388	-0.69812

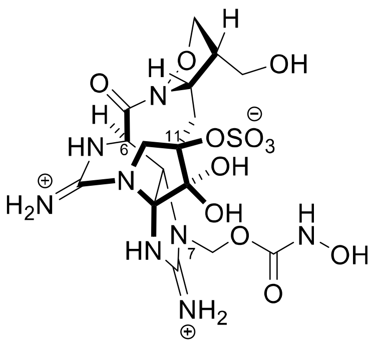

ZTX

Energy: -1483145.6066889

C	1.39107	1.22183	0.65608
N	1.48133	1.55127	-0.74541
C	0.83988	0.86163	-1.68682
C	0.28273	1.83624	1.53967
H	2.28759	1.63540	1.13023
N	0.78489	1.30590	-2.92888
H	1.12956	2.23342	-3.15773
H	2.07586	2.32650	-1.02441
N	0.19988	-0.27189	-1.33872
C	-0.90625	-1.46747	0.41025
C	-1.93490	-0.78483	-0.58862
C	-1.10353	-0.65511	-1.86615
O	-0.97276	-2.85082	0.32054
O	-1.03273	-1.01944	1.71545
H	-1.49776	0.10746	-2.54898
H	-1.05719	-1.62079	-2.38856
O	0.58371	2.02553	2.71387
C	1.41589	-0.29206	0.85886
C	0.51222	-1.04586	-0.13695
N	1.33810	-2.18467	-0.46579
C	2.61135	-2.00145	-0.11474
N	2.73588	-0.87749	0.62321
H	1.09861	-0.52092	1.88420
N	3.60126	-2.80859	-0.41661
C	3.84431	-0.56631	1.48252
H	4.56307	-2.47823	-0.27881
H	-1.93181	-1.26434	2.00267
C	-2.52697	0.56698	-0.14114
O	4.70244	0.41697	0.93193
H	3.45027	-0.12641	2.40531
H	4.40793	-1.47520	1.72716
C	5.70477	-0.00831	0.14122
O	5.89947	-1.17098	-0.16267
N	6.48207	1.03222	-0.22103
H	6.14341	1.98661	-0.11615
O	7.37903	0.82871	-1.23060
H	8.24631	0.74116	-0.80662
H	-3.34187	0.72049	-0.85758
H	-2.99239	0.46259	0.84809
N	-0.94418	2.10925	1.06793
C	-1.70041	1.86136	-0.15810
C	-2.58209	3.13378	-0.13857
C	-2.84438	3.33043	1.35256
O	-1.83241	2.57248	2.03046
H	-1.04858	1.93777	-1.02792

C	-3.83514	3.10580	-0.98720
H	-1.94130	3.95179	-0.50005
H	-2.77420	4.38396	1.65733
H	-3.81727	2.92280	1.66447
O	-3.45856	2.81972	-2.31750
H	-4.54563	2.35496	-0.59848
H	-4.33329	4.08898	-0.91083
H	-4.26140	2.71382	-2.84023
O	-3.00047	-1.66533	-0.92988
S	-4.20035	-2.07139	0.14378
O	-5.12850	-0.94233	0.20505
O	-4.72963	-3.28083	-0.47410
O	-3.46739	-2.30681	1.41528
H	3.42429	-3.61469	-1.00763
H	0.42317	0.72101	-3.67482
H	1.02439	-2.92437	-1.08665
H	-1.82977	-3.10867	0.70286

ZTX Ketone

Energy: -1435170.4188048

C	1.19470	-1.62325	-0.37651
N	1.21793	-1.51449	1.06165
C	0.51931	-0.59828	1.72842
C	0.09905	-2.47557	-1.06153
H	2.11332	-2.14232	-0.66906
N	0.40310	-0.65964	3.04069
H	0.76058	-1.46192	3.55097
H	1.79810	-2.16450	1.58513
N	-0.11242	0.37130	1.03407
C	-1.17480	0.85549	-0.98801
C	-2.23524	0.75052	0.12259
C	-1.42007	0.90396	1.42223
O	-1.37164	0.98093	-2.16133
H	-1.84796	0.31130	2.23954
H	-1.34298	1.94650	1.75129
O	0.45664	-3.32689	-1.85043
C	1.19276	-0.23938	-1.02653
C	0.22310	0.73250	-0.33910
N	0.95284	1.97545	-0.41470
C	2.25694	1.75993	-0.61344
N	2.47364	0.46074	-0.91907
H	0.92134	-0.33147	-2.09096
N	3.19137	2.67641	-0.54721
H	0.53670	2.85184	-0.10088
C	3.66521	-0.05633	-1.53643
H	4.17162	2.37620	-0.50933
C	-2.94223	-0.61368	0.04980
O	4.48317	-0.76866	-0.62803
H	3.37494	-0.78334	-2.30272
H	4.22981	0.75731	-2.00836
C	5.35853	-0.05407	0.10419
O	5.51422	1.14916	0.01272
N	6.00251	-0.86306	0.97128
H	6.00692	-1.86955	0.81507
O	7.11077	-0.36034	1.59247
H	6.84229	-0.16551	2.50301
H	-3.68019	-0.61324	0.86299
H	-3.48418	-0.65736	-0.90654
N	-1.21247	-2.05265	-0.98810
C	-2.05523	-1.84929	0.20009
C	-2.82989	-3.18852	0.22043
C	-2.92948	-3.54382	-1.26389
O	-2.02985	-2.64713	-1.93711
H	-1.44326	-1.80522	1.10363
C	-4.16264	-3.17221	0.93891
H	-2.17622	-3.91629	0.72373
H	-2.63414	-4.58321	-1.46592
H	-3.92950	-3.35850	-1.68132

O	-3.93616	-2.78970	2.27863
H	-4.85946	-2.48039	0.43288
H	-4.60846	-4.18138	0.87735
H	-4.79091	-2.70608	2.71634
O	-3.25490	1.70459	-0.05901
S	-2.88963	3.33595	0.06517
O	-3.11612	3.66047	1.47543
O	-1.47242	3.42112	-0.34372
O	-3.84091	3.92356	-0.87021
H	2.94362	3.62051	-0.26647
H	0.01412	0.11816	3.56399

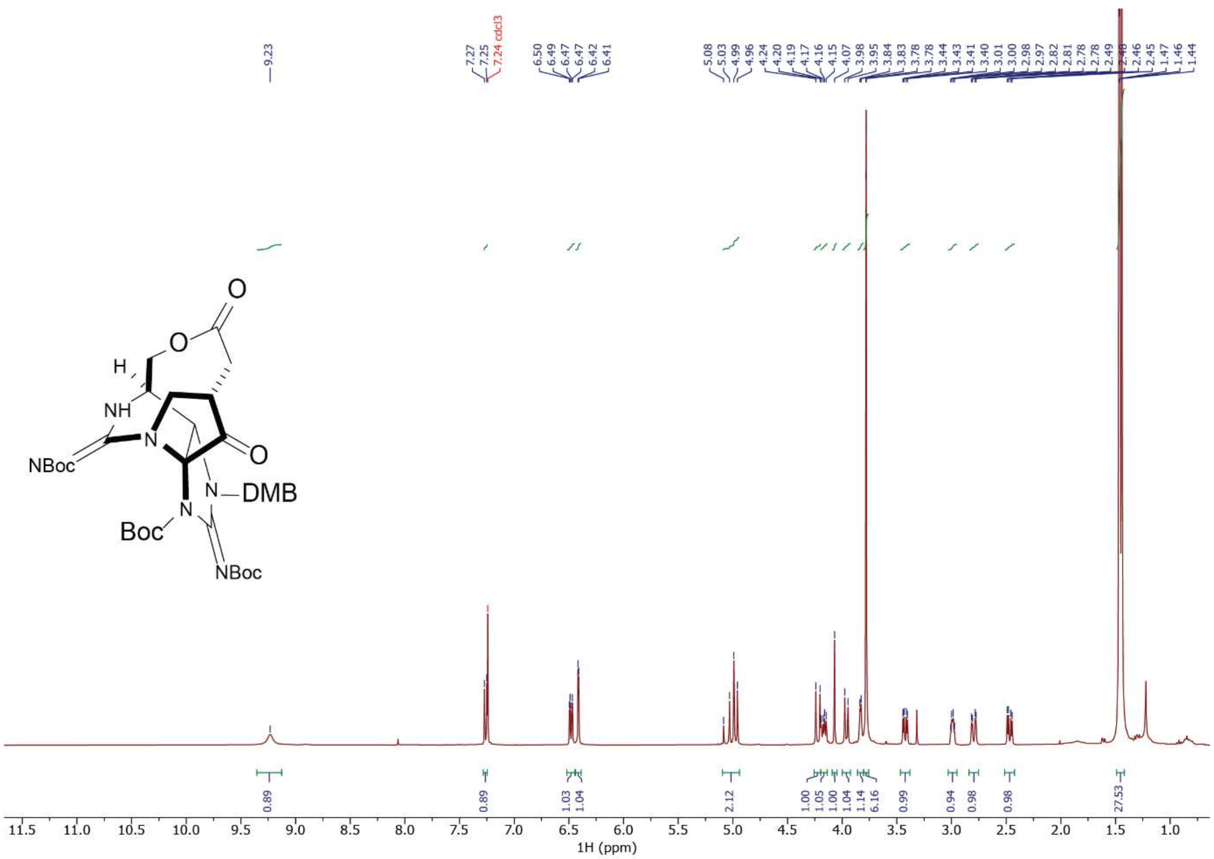

9.23
7.27
7.25
7.24 cdcl3
6.50
6.49
6.47
6.47
6.42
6.41
5.08
5.03
4.99
4.96
4.24
4.20
4.19
4.17
4.16
4.15
4.07
3.98
3.95
3.84
3.83
3.78
3.78
3.44
3.43
3.41
3.40
3.01
3.00
2.98
2.97
2.82
2.81
2.78
2.78
2.49
2.48
2.46
2.45
1.47
1.46
1.44
O
O
H
NH
N
O
NBoc
N
DMB
Boc
N
NBoc
0.89
0.89
1.03
1.04
2.12
1.00
1.05
1.00
1.04
1.14
6.16
0.99
0.94
0.98
0.98
27.53
11.5
11.0
10.5
10.0
9.5
9.0
8.5
8.0
7.5
7.0
6.5
6.0
5.5
5.0
4.5
4.0
3.5
3.0
2.5
2.0
1.5
1.0
1H (ppm)

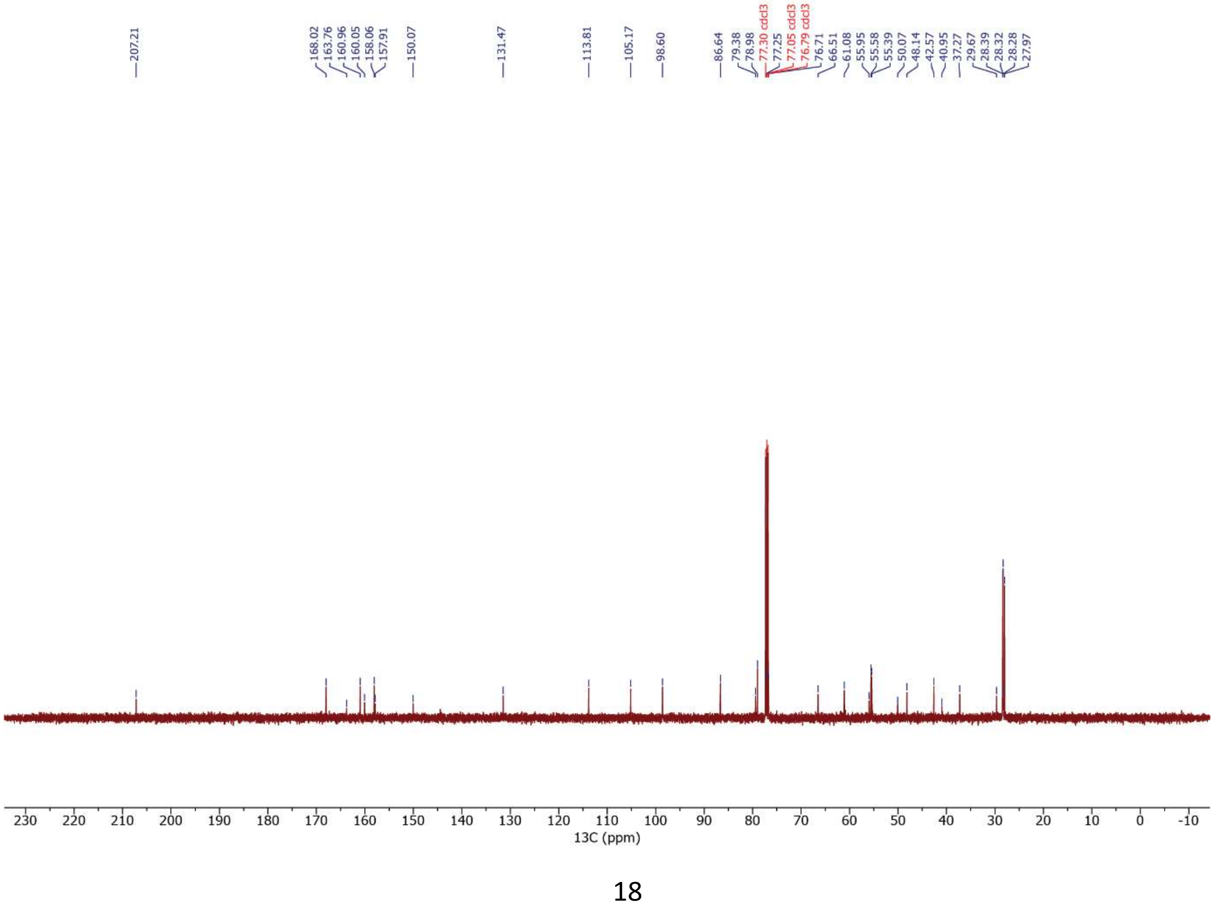

207.21
168.02
163.76
160.96
160.05
158.06
157.91
150.07
131.47
113.81
105.17
98.60
86.64
79.38
78.98
77.30 cdcl3
77.25
77.05 cdcl3
76.79 cdcl3
76.71
66.51
61.08
55.95
55.58
55.39
50.07
48.14
42.57
40.95
37.27
29.67
28.39
28.32
28.28
27.97
230
220
210
200
190
180
170
160
150
140
130
120
110
100
90
80
70
60
50
40
30
20
10
0
-10
13C (ppm)

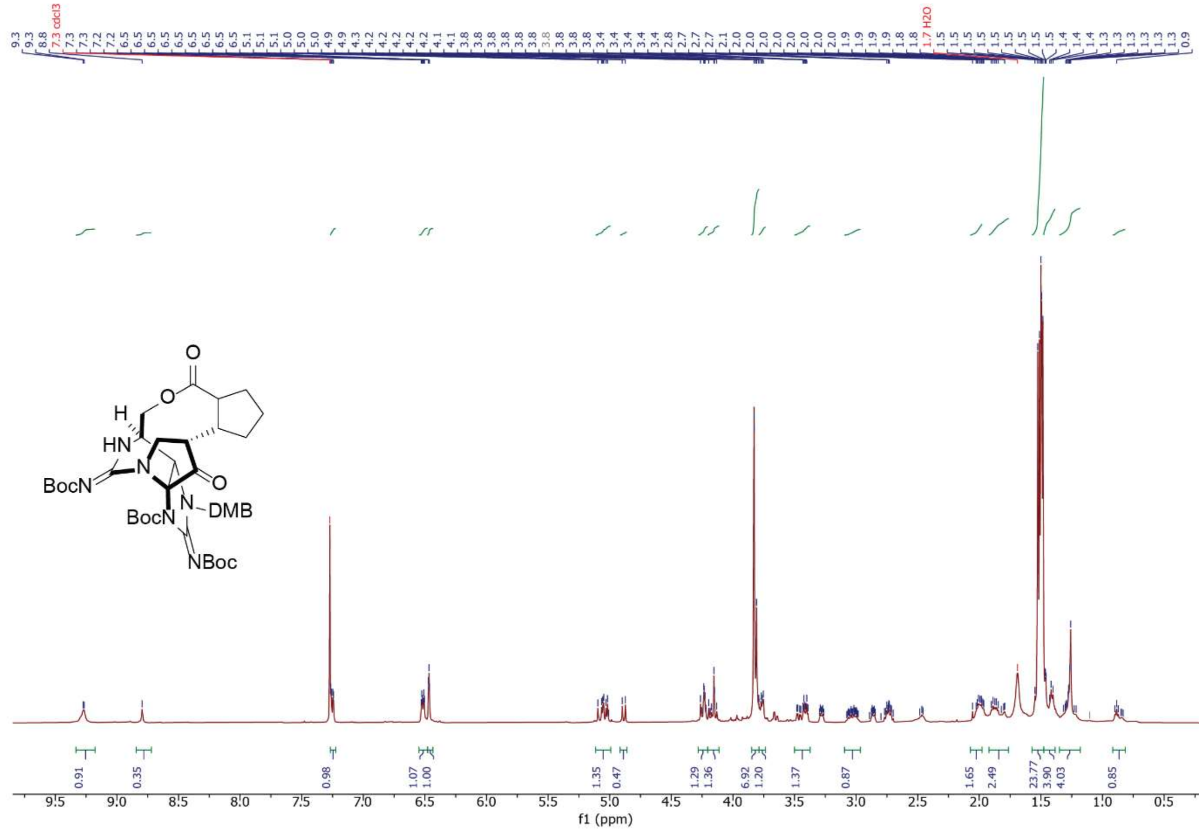
BocN
HN
H
O
O
O
BocN
N
DMB
NBoc
f1 (ppm)

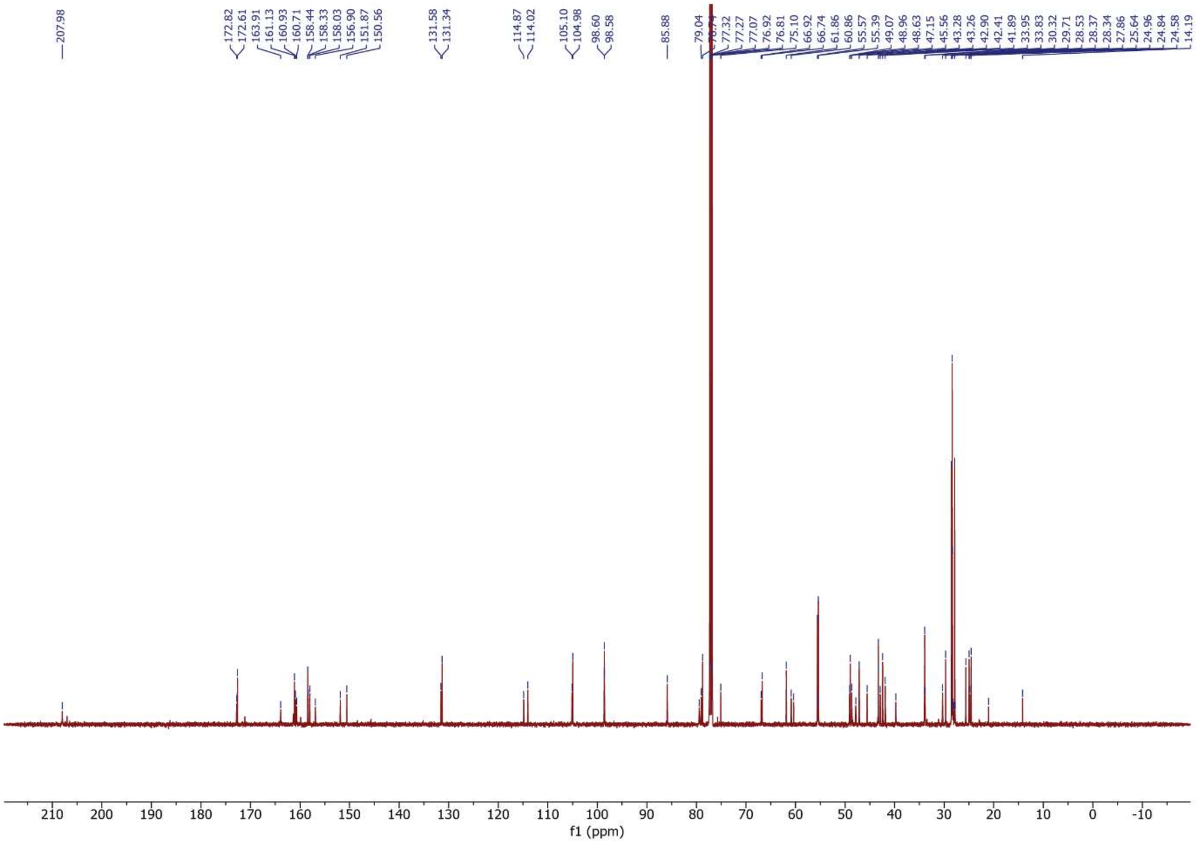
f1 (ppm)

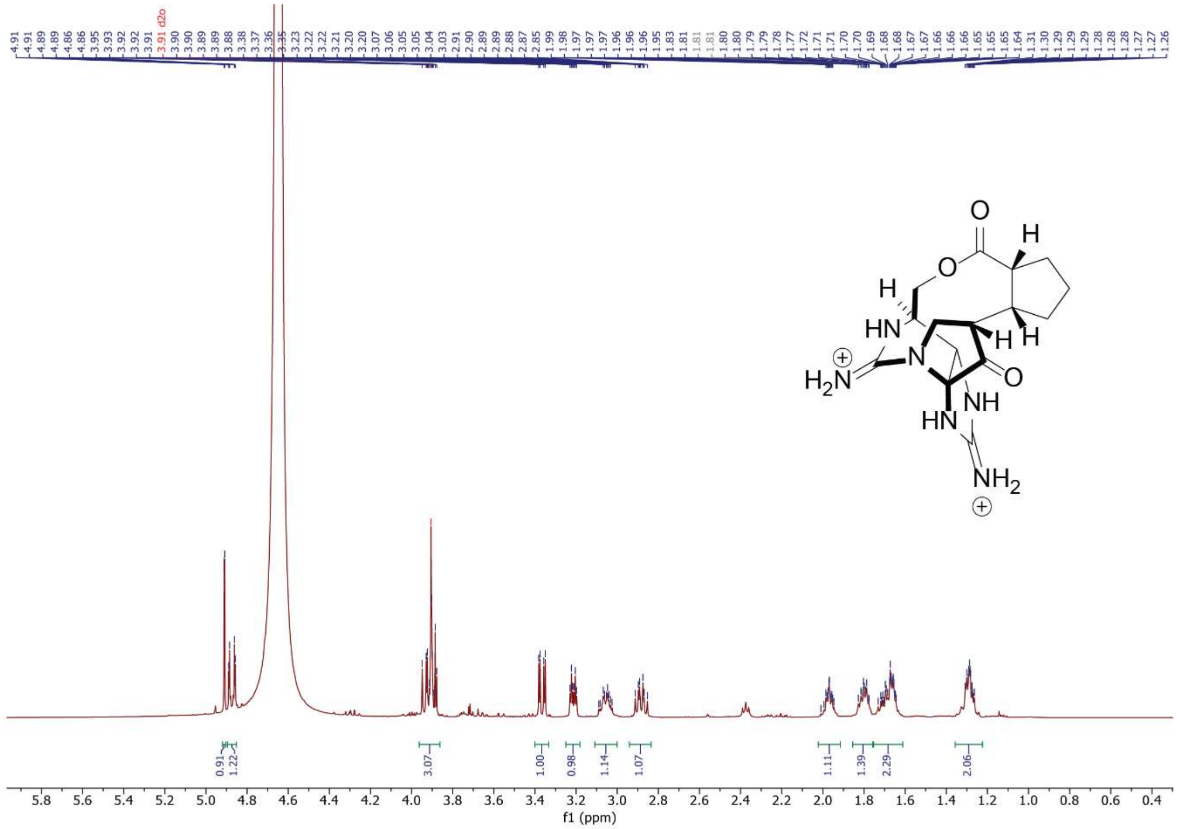
O
H
H
HN
N
H
H
O
NH
HN
NH2
H2N
f1 (ppm)

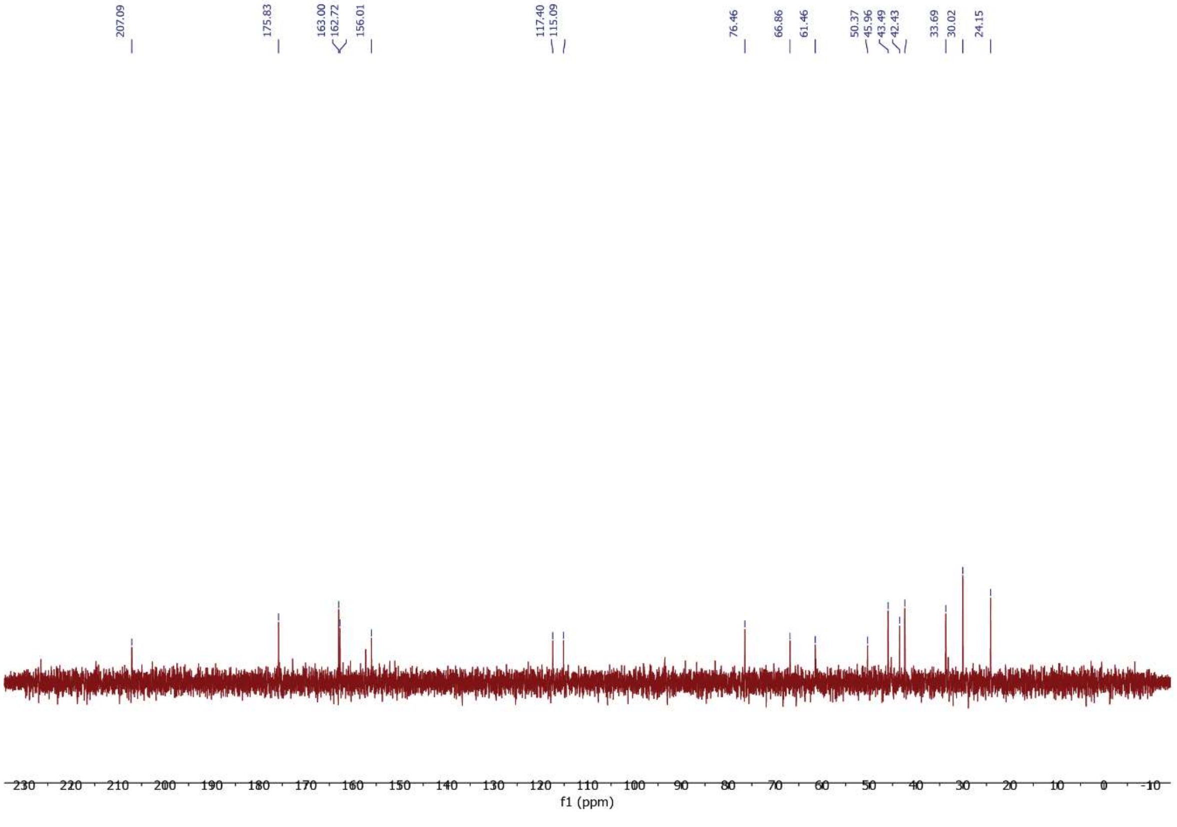
207.09
175.83
163.00
162.72
156.01
117.40
115.09
76.46
66.86
61.46
50.37
45.96
43.49
42.43
33.69
30.02
24.15
f1 (ppm)

gHMBCAD

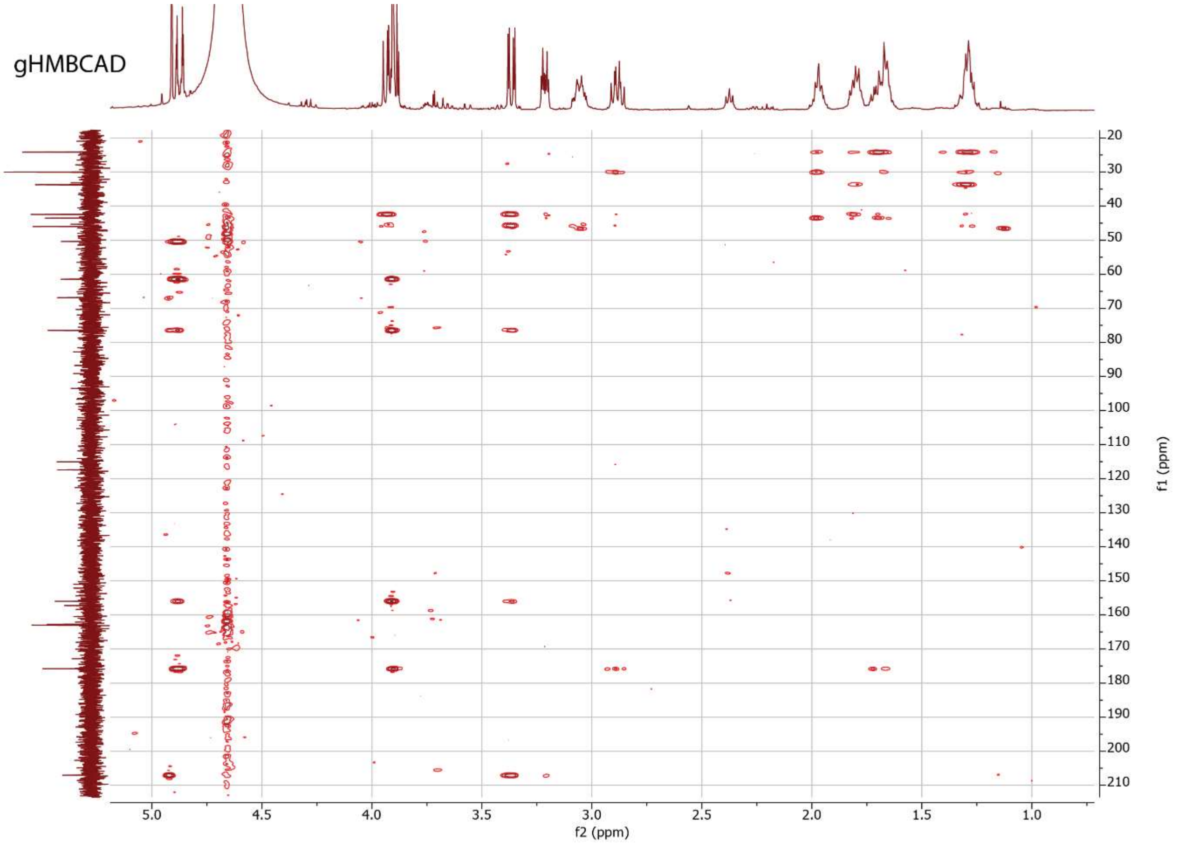

NOESY

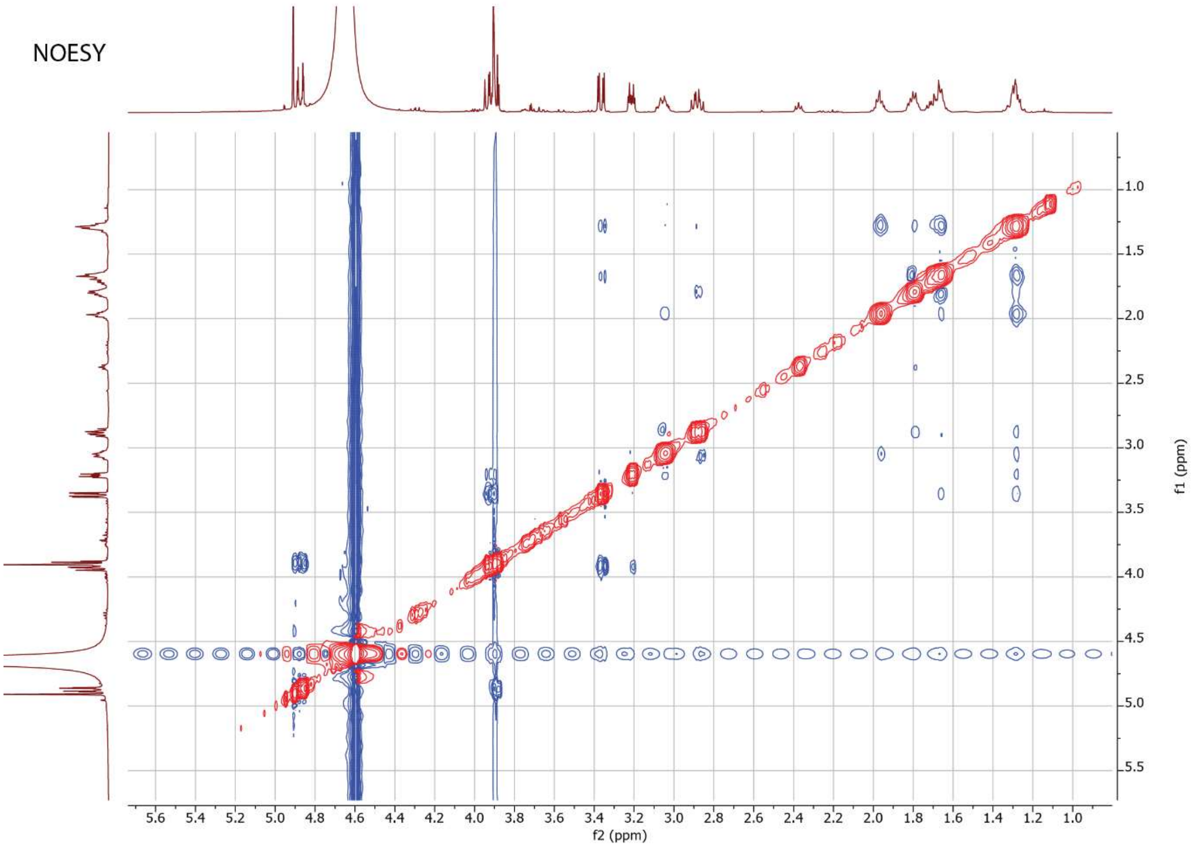

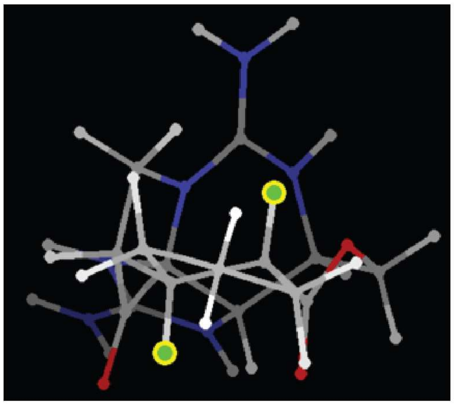

Trans-1

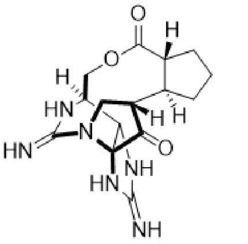

+3.32 kcal/mol

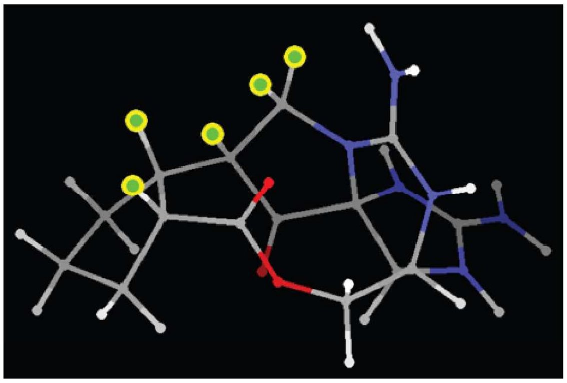

Cis-1

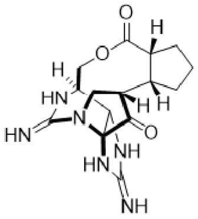

0 kcal/mol

Cis-2

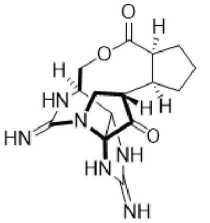

+2.34 kcal/mol

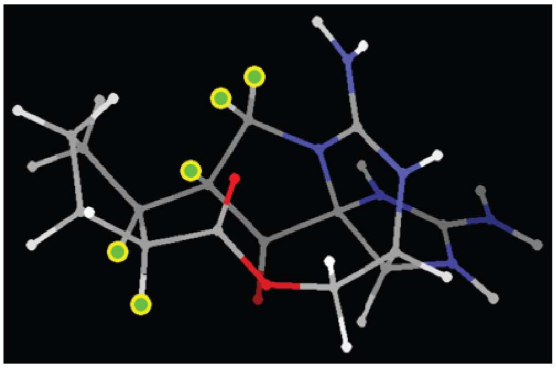

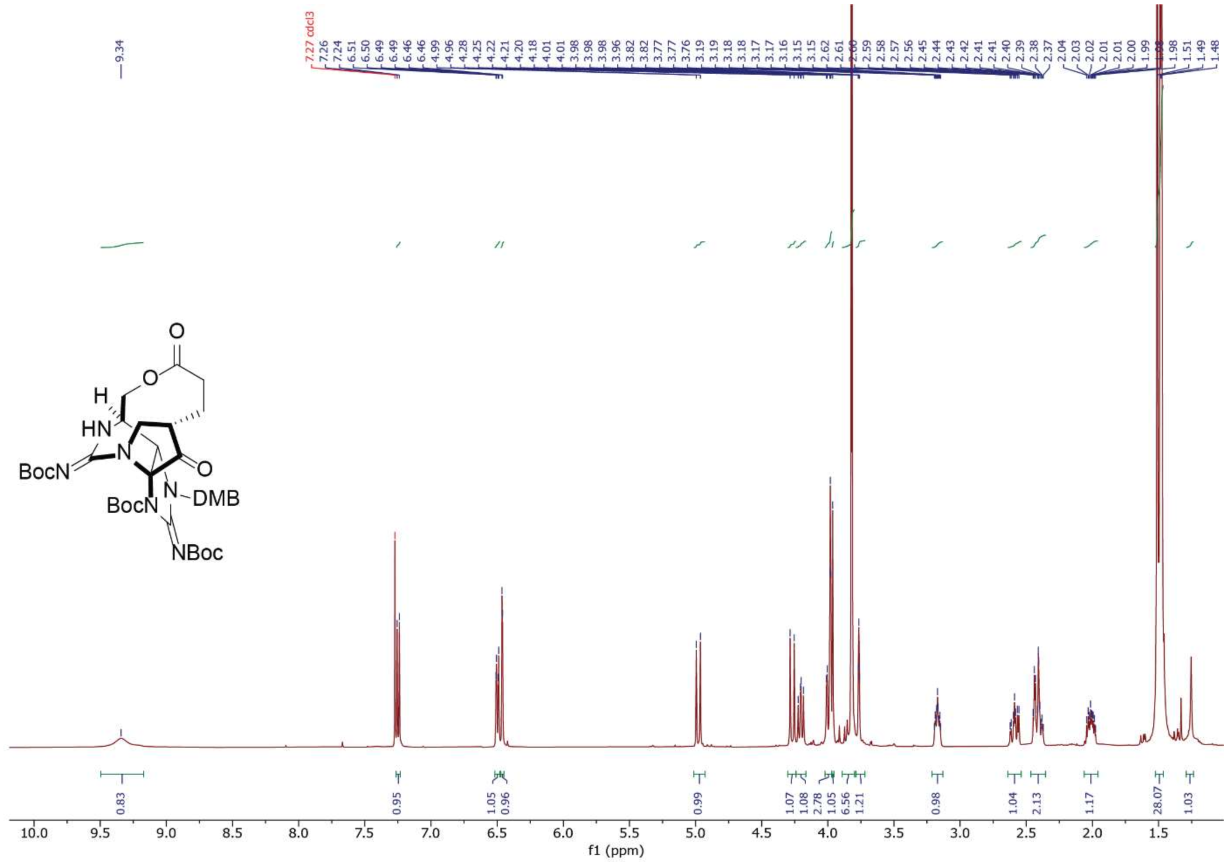
9.34
7.27 cdcl3
7.26
7.24
6.51
6.50
6.49
6.49
6.46
6.46
4.99
4.96
4.28
4.25
4.22
4.21
4.20
4.18
4.01
4.01
3.98
3.98
3.98
3.96
3.82
3.77
3.77
3.76
3.19
3.19
3.18
3.18
3.17
3.17
3.16
3.15
3.15
2.62
2.61
2.59
2.58
2.57
2.56
2.45
2.44
2.43
2.42
2.41
2.41
2.40
2.39
2.38
2.37
2.04
2.03
2.02
2.01
2.01
2.00
1.99
1.98
1.51
1.49
1.48
O
O
H
HN
N
O
BocN
BocN
N
DMB
N
NBoc
0.83
0.95
1.05
0.96
0.99
1.07
1.08
2.78
1.05
6.56
1.21
0.98
1.04
2.13
1.17
28.07
1.03
10.0 9.5 9.0 8.5 8.0 7.5 7.0 6.5 6.0 5.5 5.0 4.5 4.0 3.5 3.0 2.5 2.0 1.5
f1 (ppm)

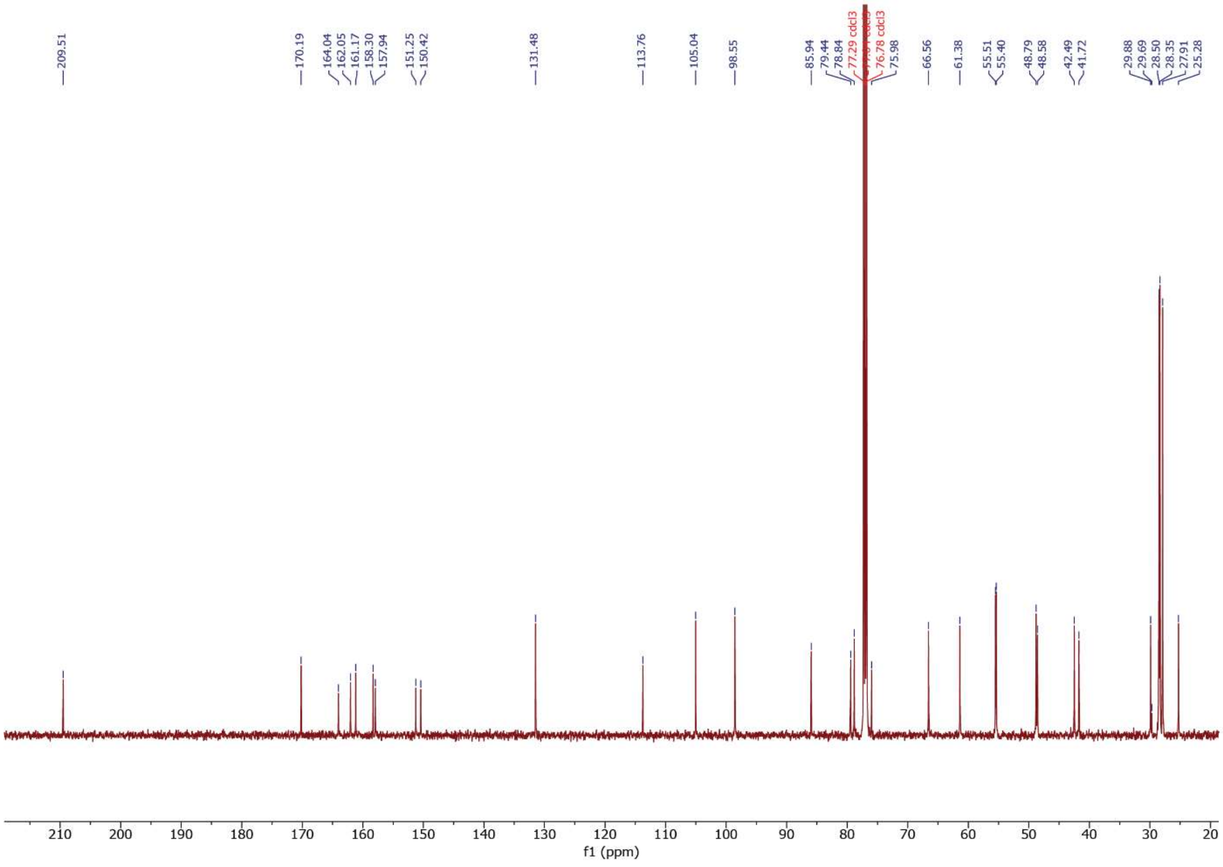
209.51
170.19
164.04
162.05
161.17
158.30
157.94
151.25
150.42
131.48
113.76
105.04
98.55
85.94
79.44
78.84
77.29 cdcl3
76.78 cdcl3
75.98
66.56
61.38
55.51
55.40
48.79
48.58
42.49
41.72
29.88
29.69
28.50
28.35
27.91
25.28
210 200 190 180 170 160 150 140 130 120 110 100 90 80 70 60 50 40 30 20
f1 (ppm)

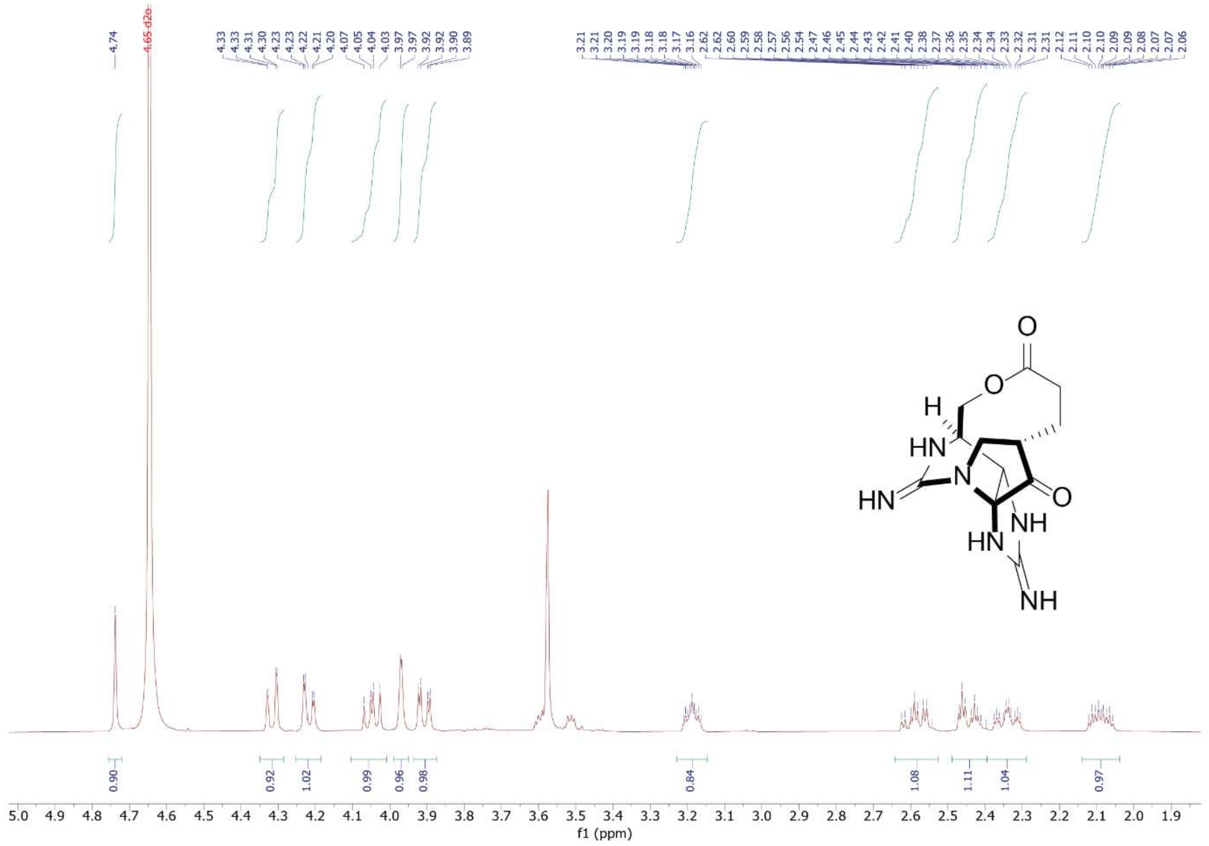
4.74
4.65 d2o
4.33 4.33 4.31 4.30 4.23 4.23 4.22 4.21 4.20 4.07 4.05 4.04 4.03 3.97 3.97 3.92 3.92 3.90 3.89
3.21 3.21 3.20 3.19 3.19 3.18 3.18 3.17 3.16 2.62 2.62 2.60 2.59 2.58 2.57 2.56 2.54 2.47 2.46 2.45 2.44 2.43 2.42 2.41 2.40 2.38 2.37 2.36 2.35 2.34 2.34 2.33 2.32 2.31 2.31 2.12 2.11 2.10 2.10 2.09 2.09 2.08 2.07 2.06
0.90
0.92
1.02
0.99
0.96
0.98
0.84
1.08
1.11
1.04
0.97
f1 (ppm)
O
O
H
HN
N
HN
O
HN
NH
NH

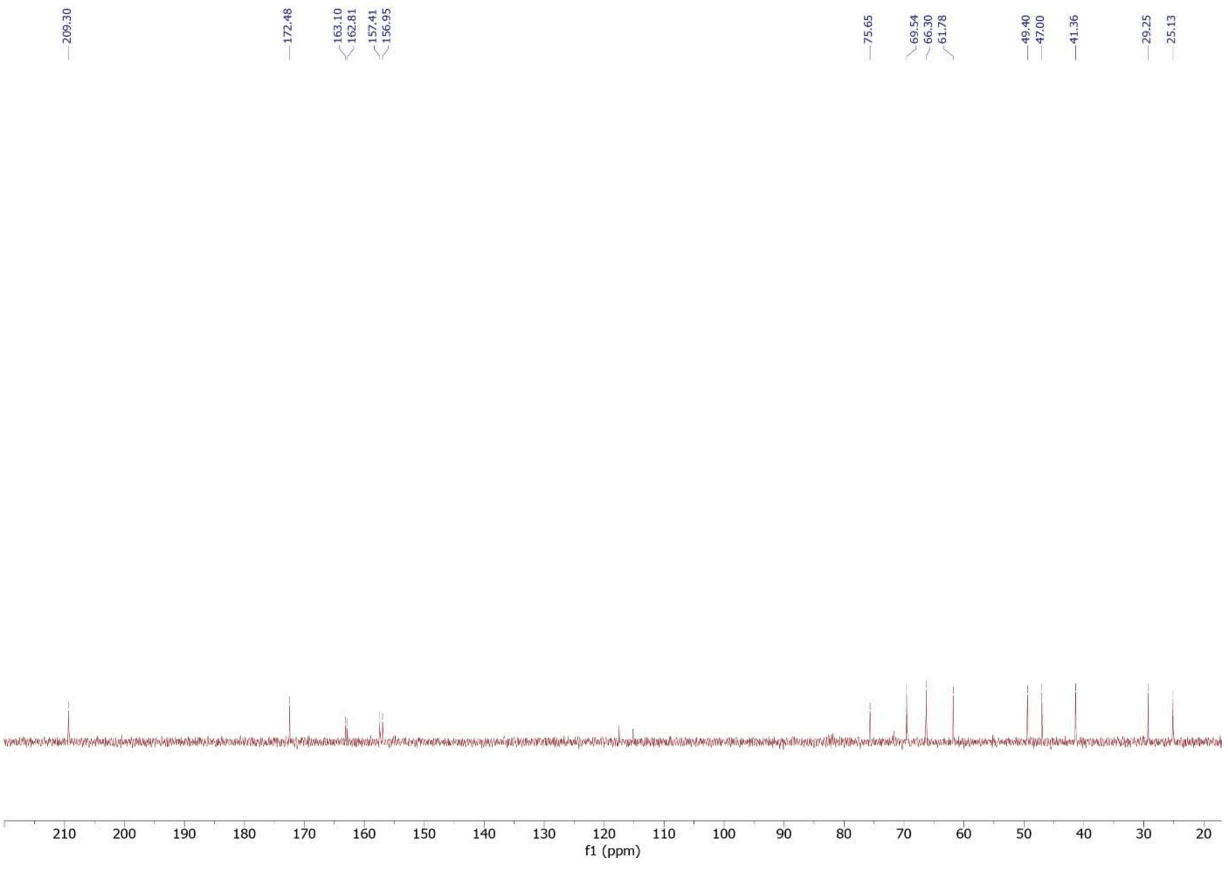
209.30
172.48
163.10 162.81
157.41 156.95
75.65
69.54 66.30 61.78
49.40 47.00
41.36
29.25 25.13
f1 (ppm)

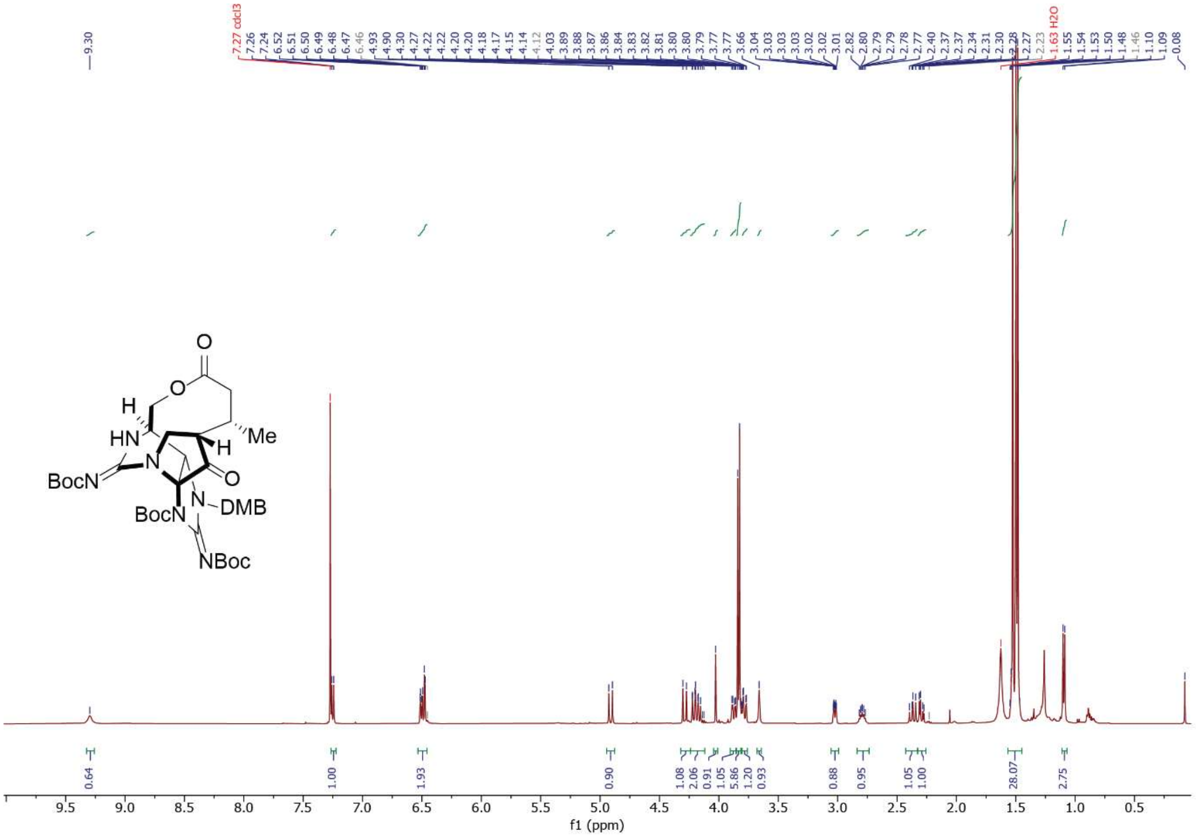
9.30
7.27 cdcl3
7.26
7.24
6.52
6.51
6.50
6.49
6.48
6.47
6.46
4.93
4.90
4.30
4.27
4.22
4.22
4.20
4.20
4.18
4.17
4.15
4.14
4.12
4.03
3.89
3.88
3.87
3.86
3.84
3.83
3.82
3.81
3.80
3.80
3.79
3.77
3.77
3.66
3.04
3.03
3.03
3.02
3.02
3.01
2.82
2.80
2.79
2.79
2.78
2.77
2.40
2.37
2.37
2.34
2.31
2.30
2.28
2.27
2.23
1.63 H2O
1.55
1.54
1.53
1.50
1.48
1.46
1.10
1.09
0.08
O
H
O
HN
H
Me
BocN
N
O
BocN
N
DMB
NBoc
0.64
1.00
1.93
0.90
1.08
2.06
0.91
1.05
5.86
1.20
0.93
0.88
0.95
1.05
1.00
28.07
2.75
9.5
9.0
8.5
8.0
7.5
7.0
6.5
6.0
5.5
5.0
4.5
4.0
3.5
3.0
2.5
2.0
1.5
1.0
0.5
f1 (ppm)

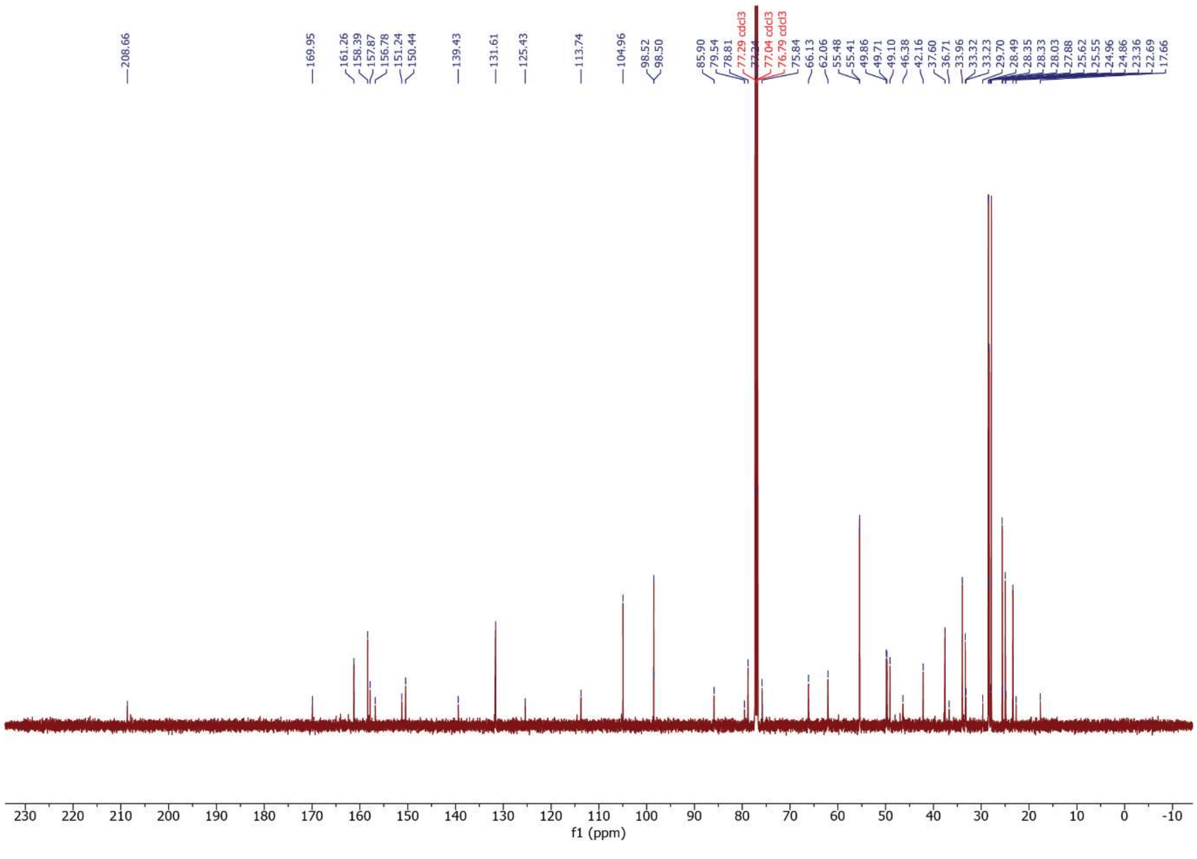
208.66
169.95
161.26
158.39
157.87
156.78
151.24
150.44
139.43
131.61
125.43
113.74
104.96
98.52
98.50
85.90
79.54
78.81
77.29 cdcl3
77.04 cdcl3
76.79 cdcl3
75.84
66.13
62.06
55.48
55.41
49.86
49.71
49.10
46.38
42.16
37.60
36.71
33.96
33.32
33.23
29.70
28.49
28.35
28.33
28.03
27.88
25.62
25.55
24.96
24.86
23.36
22.69
17.66
230
220
210
200
190
180
170
160
150
140
130
120
110
100
90
80
70
60
50
40
30
20
10
0
-10
f1 (ppm)

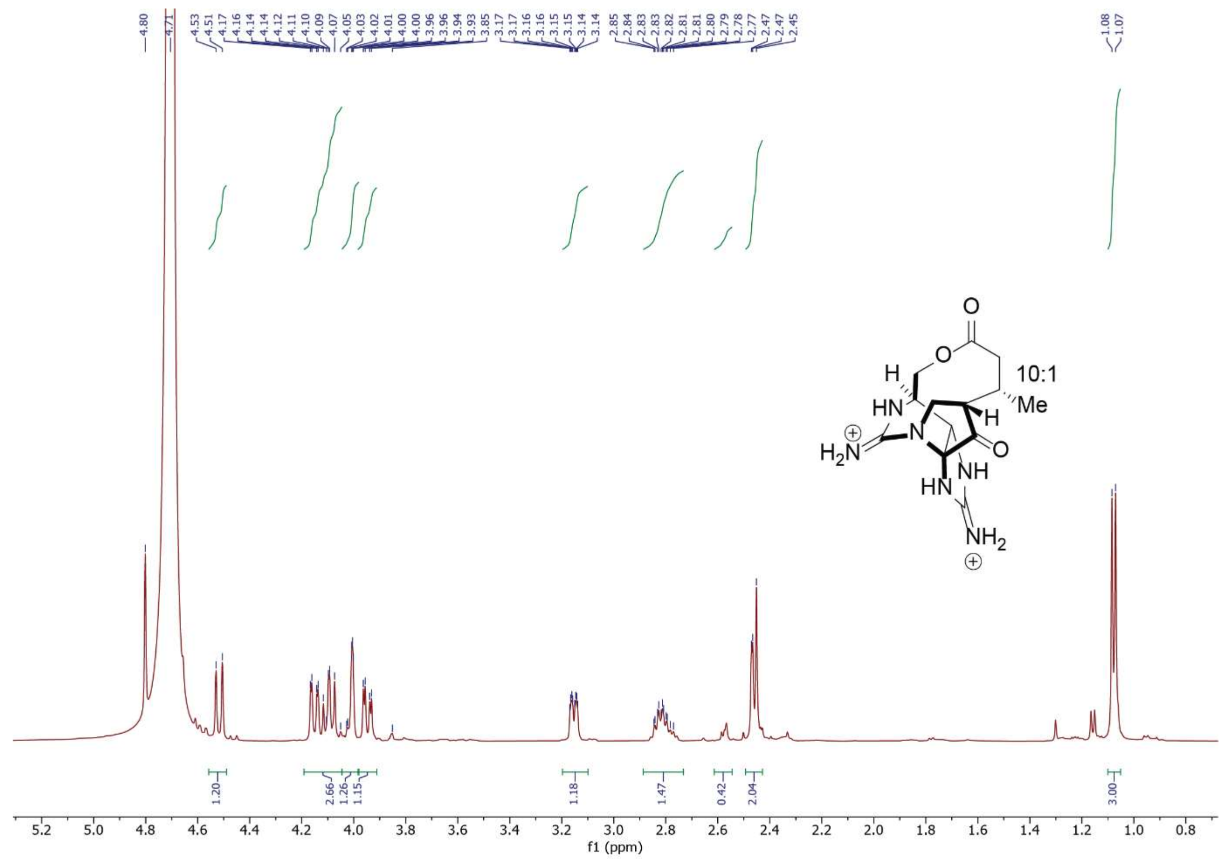
4.80
4.71
4.53 4.51 4.17 4.16 4.14 4.14 4.12 4.11 4.10 4.09 4.07 4.05 4.03 4.02 4.01 4.00 4.00 3.96 3.96 3.94 3.93 3.85
3.17 3.17 3.16 3.16 3.15 3.15 3.14 3.14
2.85 2.84 2.83 2.83 2.82 2.81 2.81 2.80 2.79 2.78 2.77 2.47 2.47 2.45
1.08 1.07
10:1
Me
1.20
2.66
1.26
1.15
1.18
1.47
0.42
2.04
3.00
f1 (ppm)

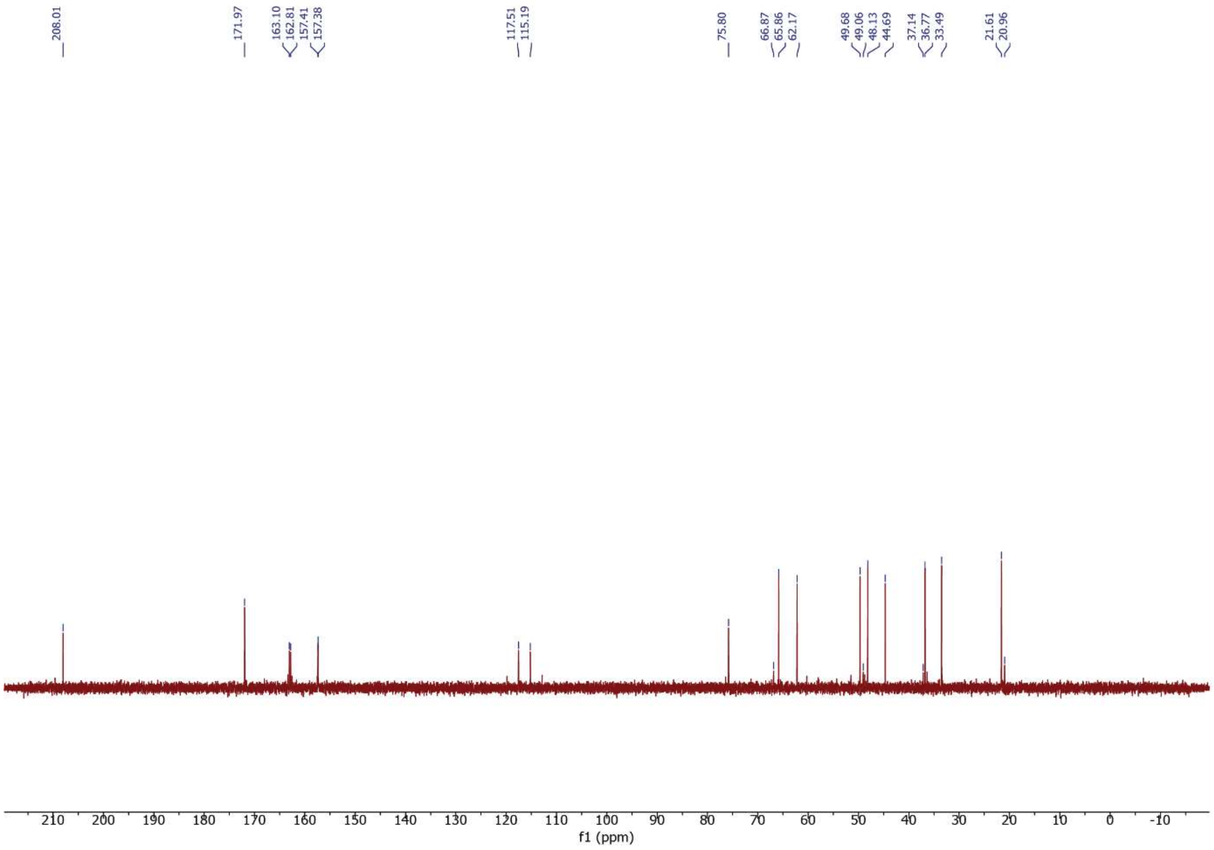
208.01
171.97
163.10 162.81 157.41 157.38
117.51 115.19
75.80
66.87 65.86 62.17
49.68 49.06 48.13 44.69
37.14 36.77 33.49
21.61 20.96
f1 (ppm)

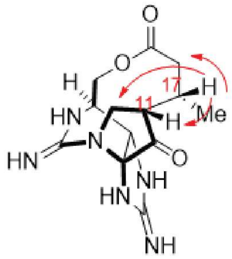

S-isomer
0 kcal/mol

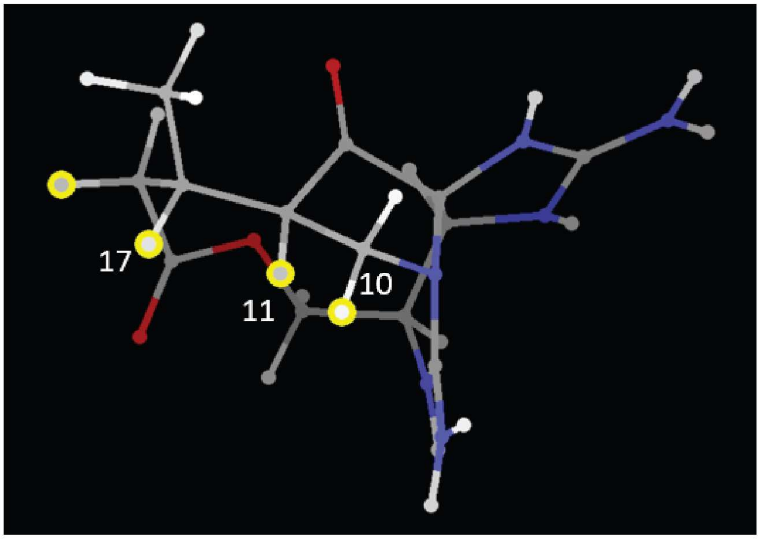

R-isomer
+1.3 kcal/mol

$A^{1,3}$ strain with
C12 ketone

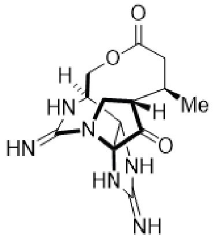

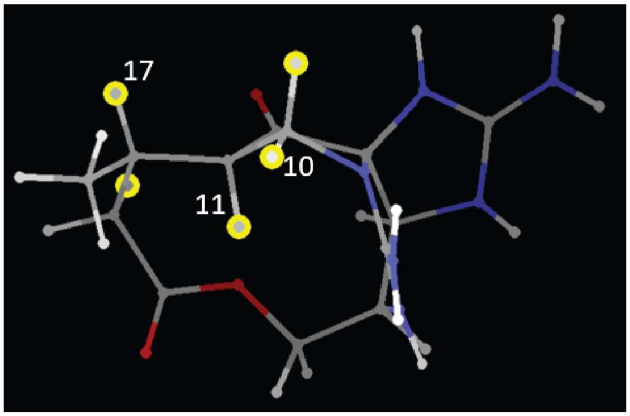

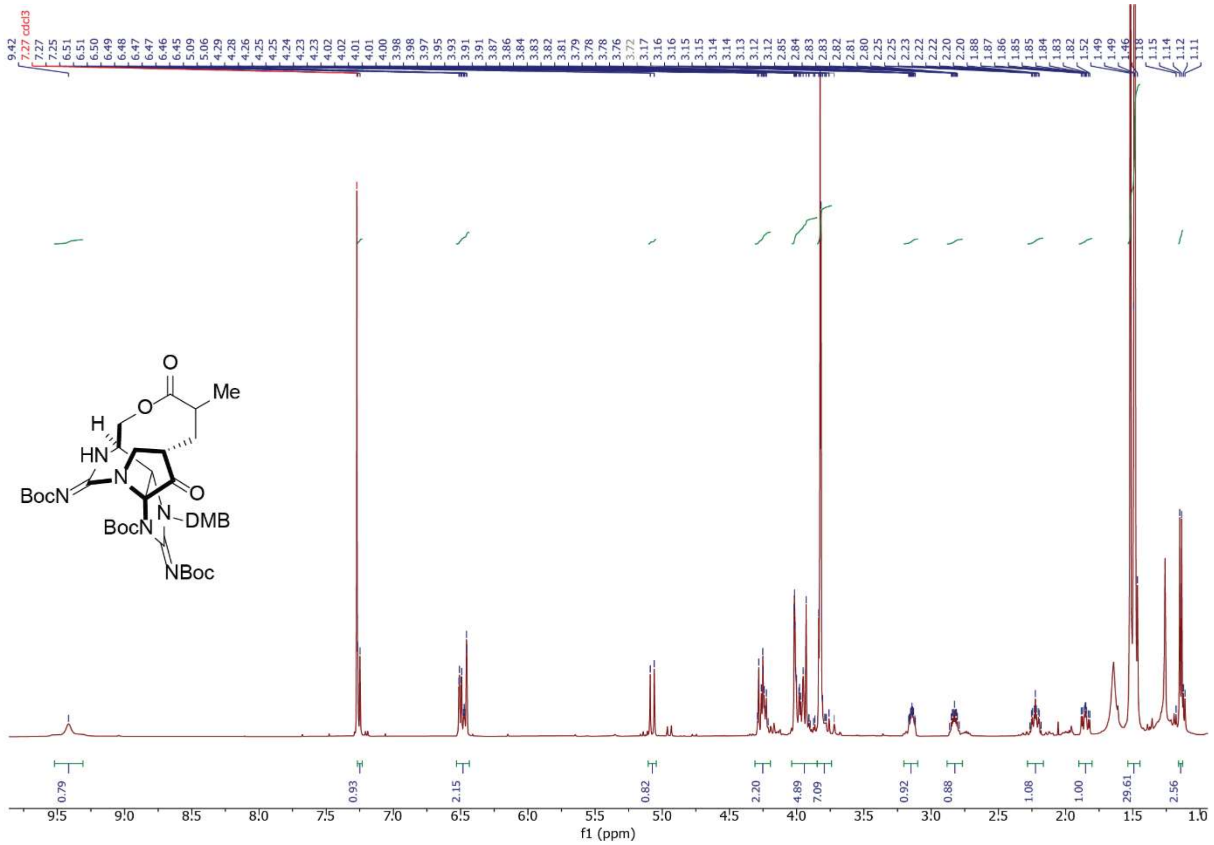

9.42
7.27 cdcl3
7.27
7.25
6.51
6.51
6.50
6.49
6.48
6.47
6.46
6.45
5.09
5.06
4.29
4.28
4.26
4.25
4.25
4.24
4.23
4.23
4.02
4.02
4.01
4.01
4.00
3.98
3.98
3.97
3.95
3.93
3.91
3.91
3.87
3.86
3.84
3.83
3.82
3.81
3.79
3.78
3.78
3.76
3.72
3.17
3.16
3.16
3.15
3.15
3.14
3.14
3.13
3.12
3.12
3.12
2.85
2.84
2.83
2.83
2.82
2.81
2.80
2.25
2.23
2.22
2.22
2.20
2.20
1.88
1.87
1.86
1.85
1.85
1.84
1.83
1.82
1.52
1.49
1.46
1.18
1.15
1.14
1.12
1.11
O
Me
O
H
HN
BocN
N
O
BocN
N
DMB
NBoc
0.79
0.93
2.15
0.82
2.20
4.89
7.09
0.92
0.88
1.08
1.00
29.61
2.56
9.5
9.0
8.5
8.0
7.5
7.0
6.5
6.0
5.5
5.0
4.5
4.0
3.5
3.0
2.5
2.0
1.5
1.0
f1 (ppm)

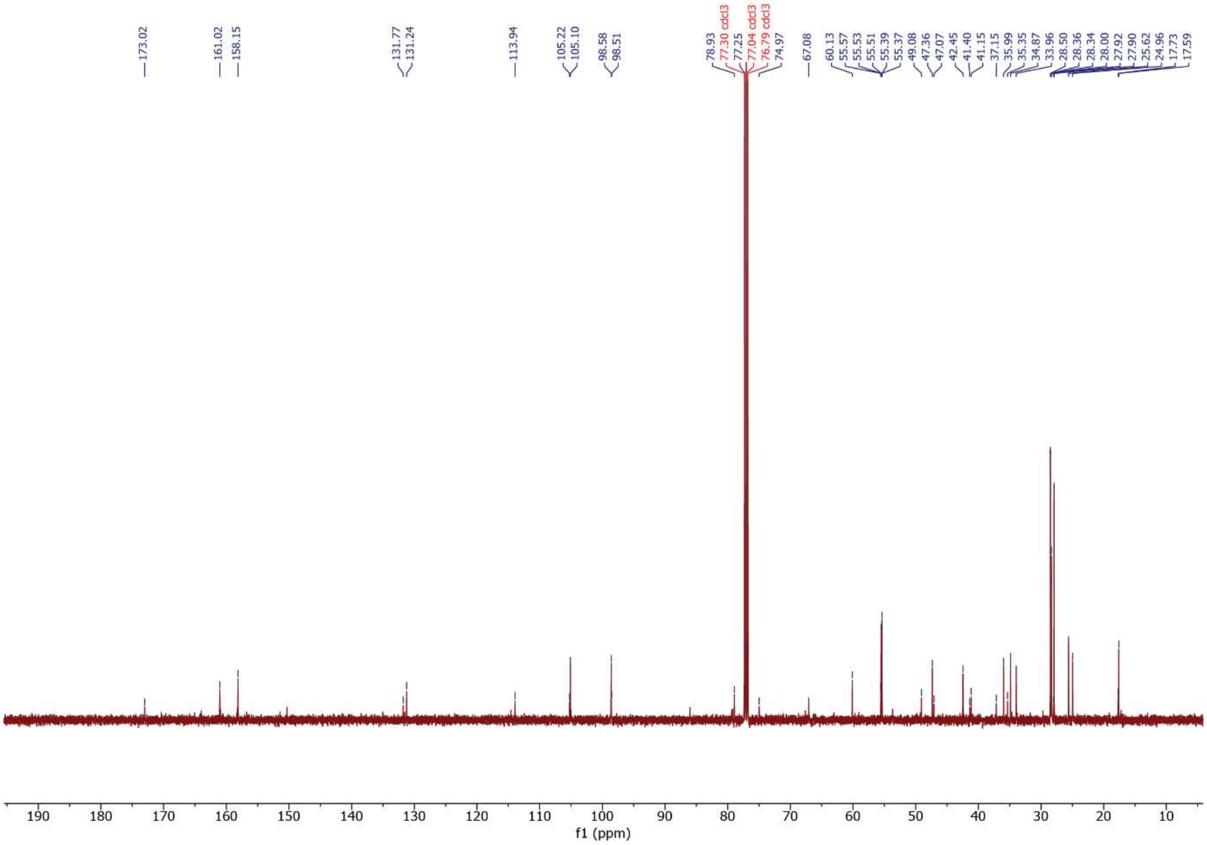

173.02
161.02
158.15
131.77
131.24
113.94
105.22
105.10
98.58
98.51
78.93
77.30 cdcl3
77.25
77.04 cdcl3
76.79 cdcl3
74.97
67.08
60.13
55.57
55.53
55.51
55.39
55.37
49.08
47.36
47.07
42.45
41.40
41.15
37.15
35.99
35.35
34.87
33.96
28.50
28.36
28.34
28.00
27.92
27.90
25.62
24.96
17.73
17.59
190
180
170
160
150
140
130
120
110
100
90
80
70
60
50
40
30
20
10
f1 (ppm)

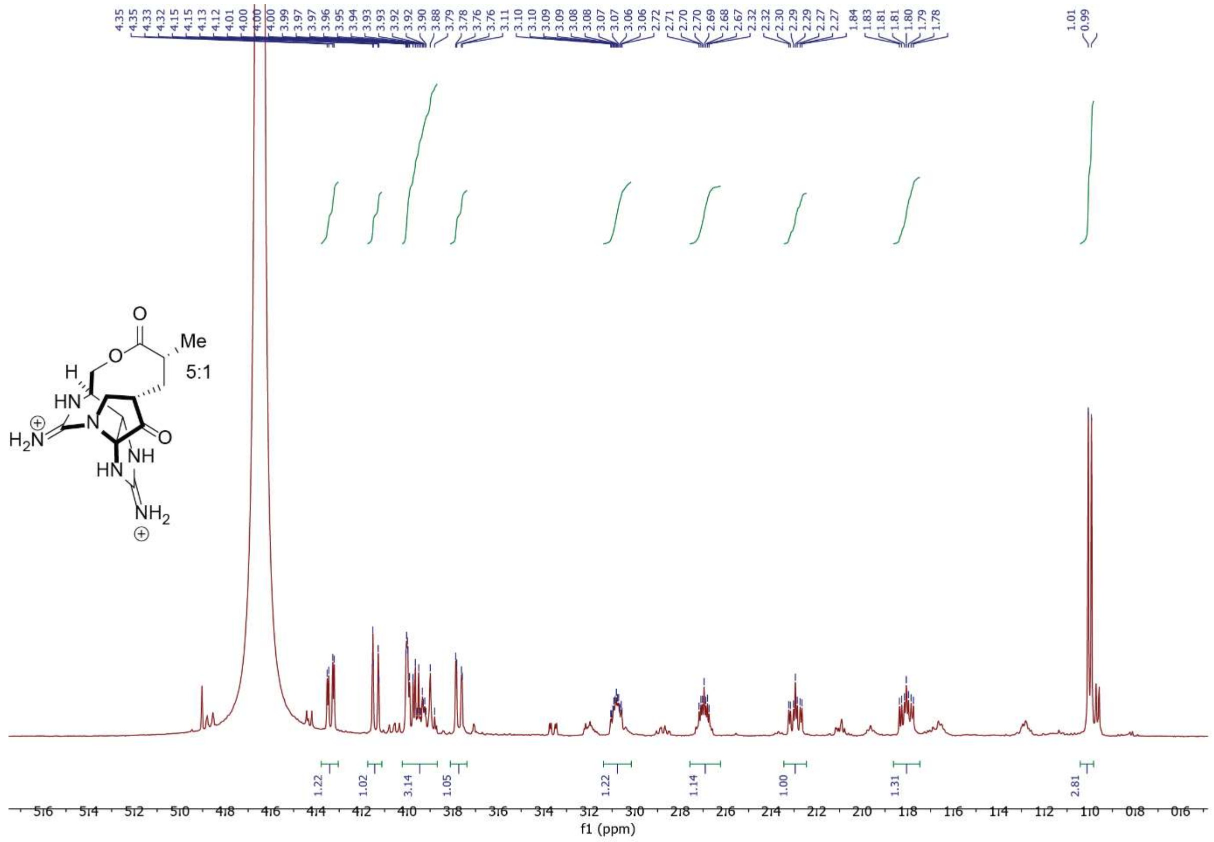
H
O
Me
5:1
HN
N
O
NH
HN
NH2
H2N
f1 (ppm)

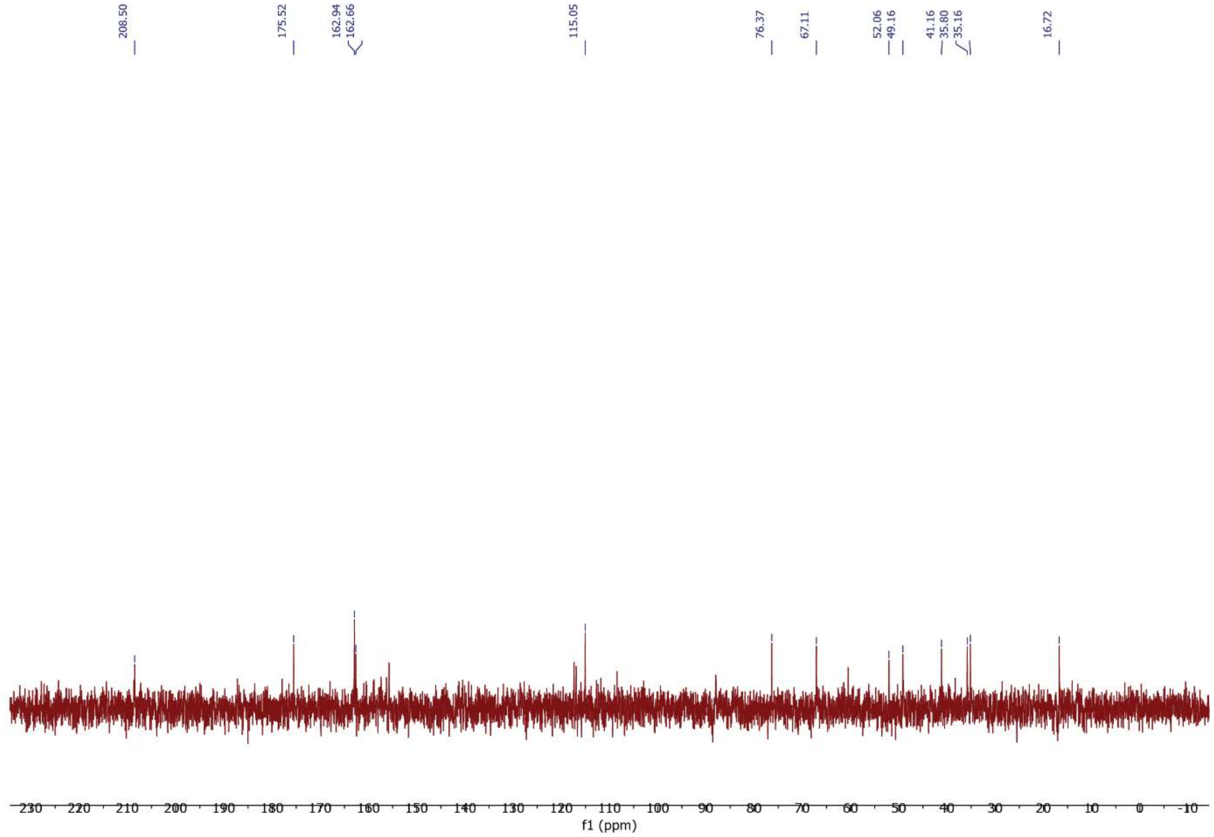
208.50
175.52
162.94
162.66
115.05
76.37
67.11
52.06
49.16
41.16
35.80
35.16
16.72
f1 (ppm)

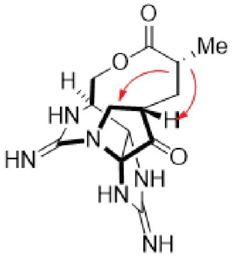

R-isomer
0 kcal/mol

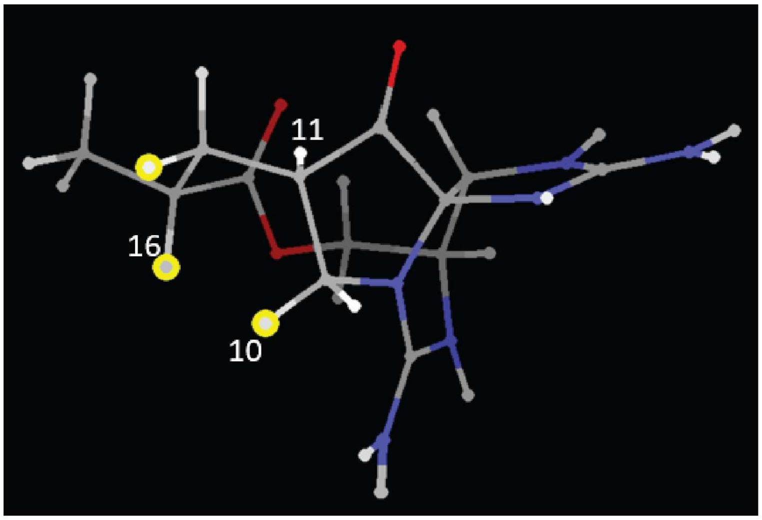

S-isomer
+1.33 kcal/mol

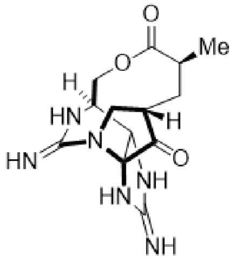

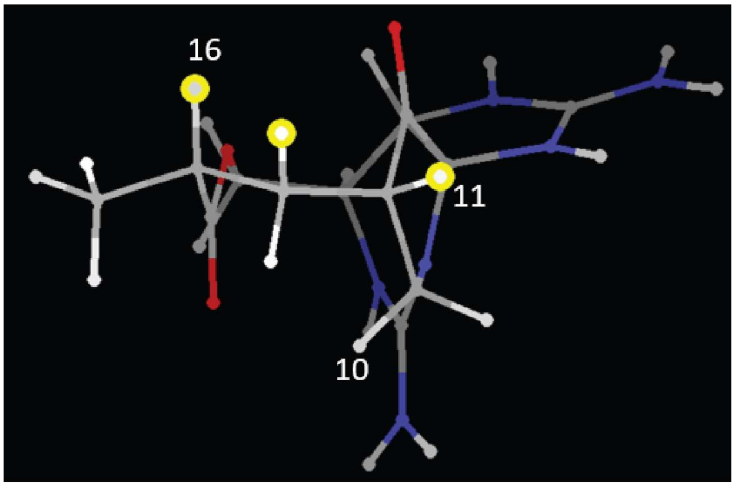

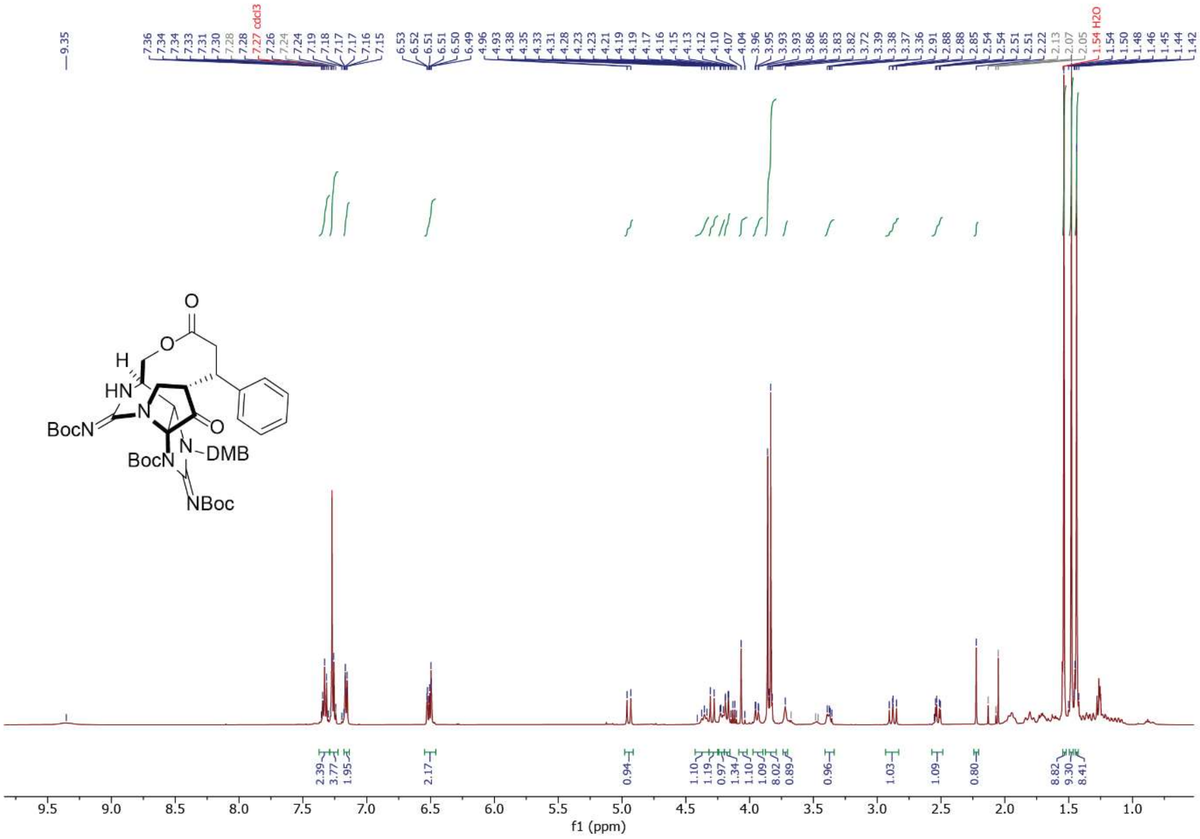
9.35
7.36 7.34 7.33 7.31 7.30 7.28 7.28 7.27 cdcl3 7.26 7.24 7.24 7.19 7.18 7.17 7.17 7.16 7.15
6.53 6.52 6.51 6.51 6.50 6.49
4.96 4.93 4.38 4.35 4.33 4.31 4.28 4.23 4.23 4.21 4.19 4.19 4.17 4.16 4.15 4.13 4.12 4.10 4.07 4.04
3.96 3.95 3.93 3.93 3.86 3.85 3.83 3.82 3.72 3.39 3.38 3.37 3.36
2.91 2.88 2.88 2.85 2.54 2.54 2.51 2.51 2.22 2.13 2.07 2.05
1.54 H2O 1.54 1.50 1.48 1.46 1.45 1.44 1.42
O
O
H
HN
BocN
N
O
BocN
N
DMB
NBoc
2.39 3.77 1.95 2.17 0.94 1.10 1.19 0.97 1.34 1.10 1.09 8.02 0.89 0.96 1.03 1.09 0.80 8.82 9.30 8.41
f1 (ppm)

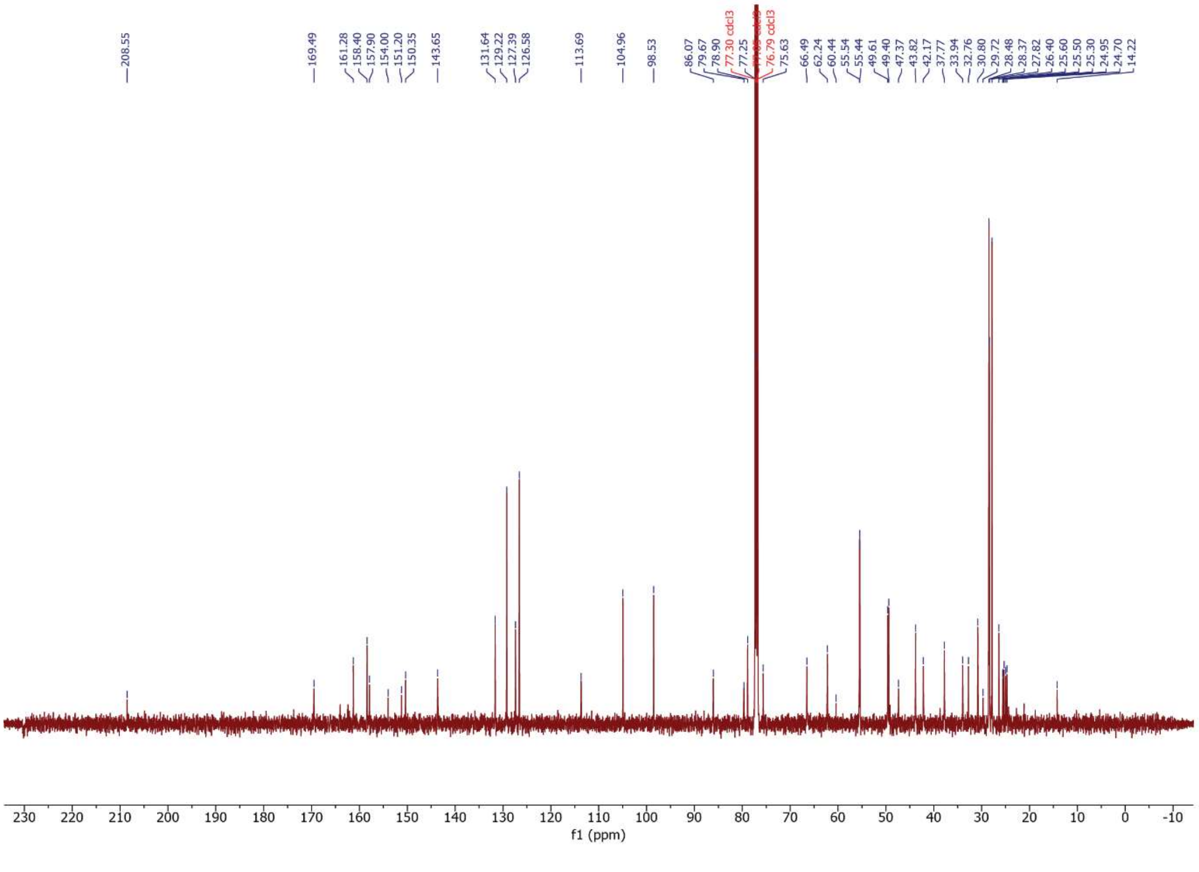
208.55
169.49 161.28 158.40 157.90 154.00 151.20 150.35 143.65
131.64 129.22 127.39 126.58 113.69 104.96 98.53
86.07 79.67 78.90 77.30 cdcl3 77.25 76.79 cdcl3 75.63
66.49 62.24 60.44 55.54 55.44 49.61 49.40 47.37 43.82 42.17 37.77 33.94 32.76 30.80 29.72 28.48 28.37 27.82 26.40 25.60 25.50 25.30 24.95 24.70 14.22
f1 (ppm)

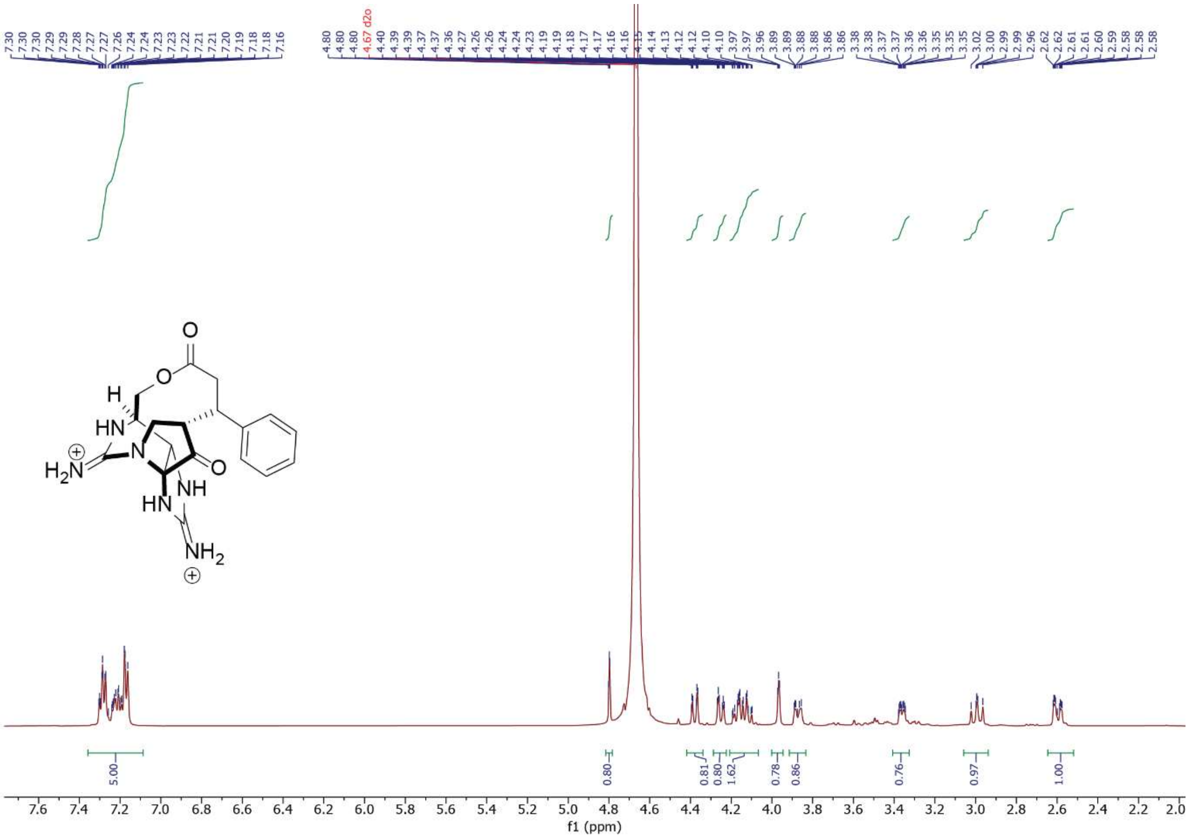
7.30 7.30 7.30 7.29 7.29 7.28 7.27 7.27 7.26 7.24 7.24 7.23 7.23 7.22 7.21 7.21 7.20 7.19 7.18 7.16
4.80 4.80 4.80 4.67 d2o 4.40 4.39 4.39 4.37 4.37 4.36 4.27 4.26 4.26 4.24 4.24 4.23 4.19 4.19 4.18 4.17 4.17 4.16 4.16 4.16 4.15 4.14 4.13 4.12 4.12 4.12 4.10 4.10 3.97 3.97 3.96 3.89 3.89 3.88 3.88 3.86 3.86 3.38 3.38 3.37 3.37 3.36 3.36 3.35 3.35 3.35 3.02 3.00 2.99 2.99 2.96 2.62 2.62 2.61 2.61 2.60 2.59 2.58 2.58 2.58
O
O
H
HN
N
H_2N
⊕
O
NH
HN
NH_2
⊕
5.00
0.80
0.81
0.80
1.62
0.78
0.86
0.76
0.97
1.00
f1 (ppm)

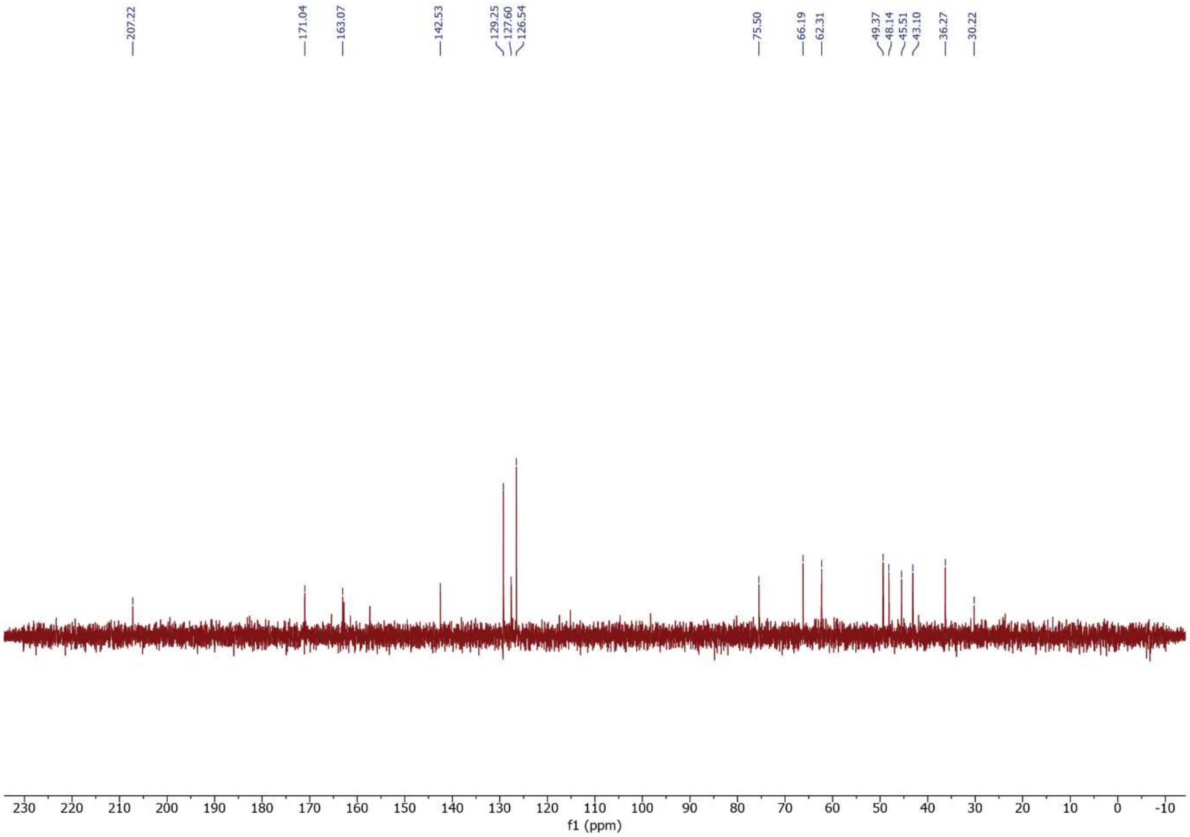
207.22
171.04
163.07
142.53
129.25
127.60
126.54
75.50
66.19
62.31
49.37
48.14
45.51
43.10
36.27
30.22
f1 (ppm)

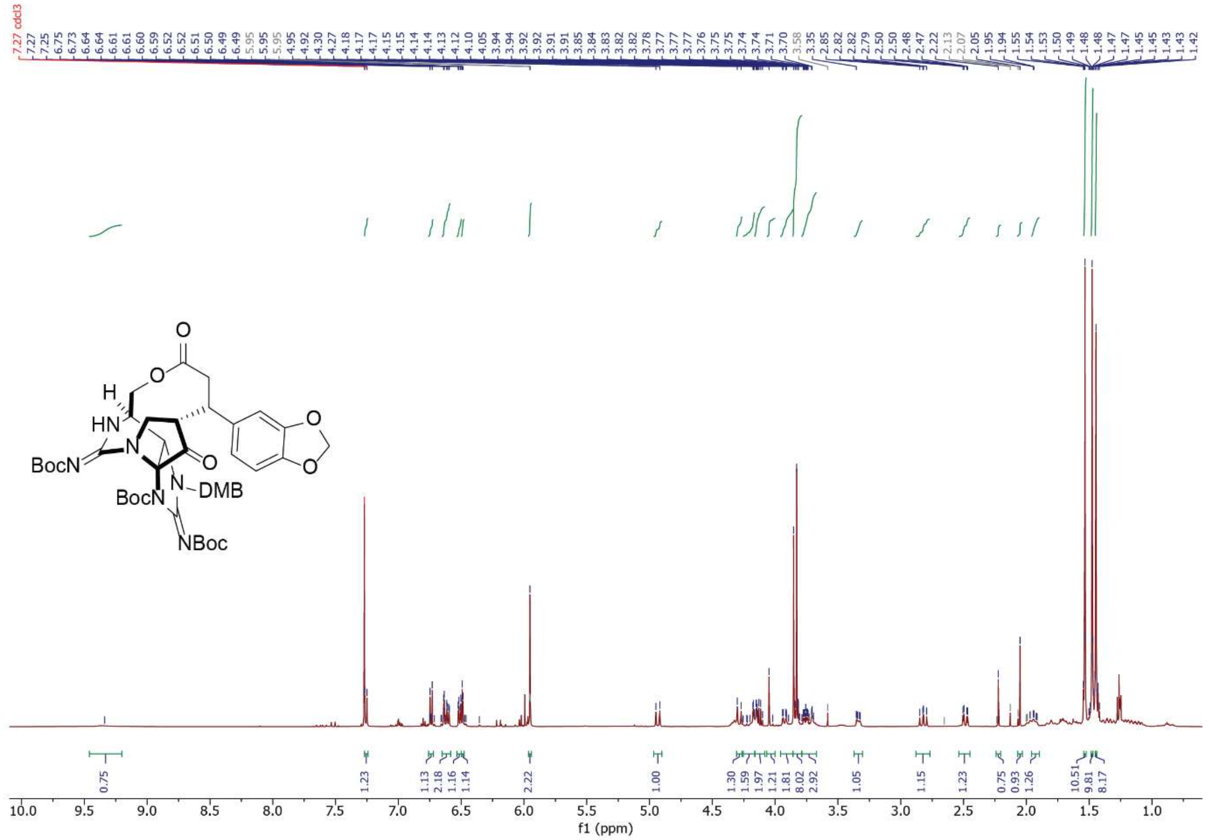
7.27 cdcl3
7.27
7.25
6.75
6.73
6.64
6.64
6.61
6.61
6.60
6.59
6.52
6.52
6.51
6.50
6.49
6.49
5.95
5.95
5.95
4.95
4.92
4.30
4.27
4.18
4.17
4.17
4.15
4.15
4.14
4.14
4.13
4.12
4.10
4.05
3.94
3.94
3.92
3.92
3.91
3.91
3.85
3.84
3.83
3.82
3.82
3.78
3.77
3.77
3.77
3.76
3.75
3.75
3.74
3.74
3.71
3.70
3.58
3.35
2.85
2.82
2.82
2.79
2.50
2.50
2.48
2.47
2.22
2.13
2.07
2.05
1.95
1.94
1.55
1.54
1.53
1.50
1.49
1.48
1.48
1.47
1.47
1.45
1.45
1.43
1.43
1.42
O
H
O
HN
N
O
O
O
BocN
BocN
N
DMB
NBoc
0.75
1.23
1.13
2.18
1.16
1.14
2.22
1.00
1.30
1.59
1.97
1.21
1.81
8.02
2.92
1.05
1.15
1.23
0.75
0.93
1.26
10.51
9.81
8.17
10.0
9.5
9.0
8.5
8.0
7.5
7.0
6.5
6.0
5.5
5.0
4.5
4.0
3.5
3.0
2.5
2.0
1.5
1.0
f1 (ppm)

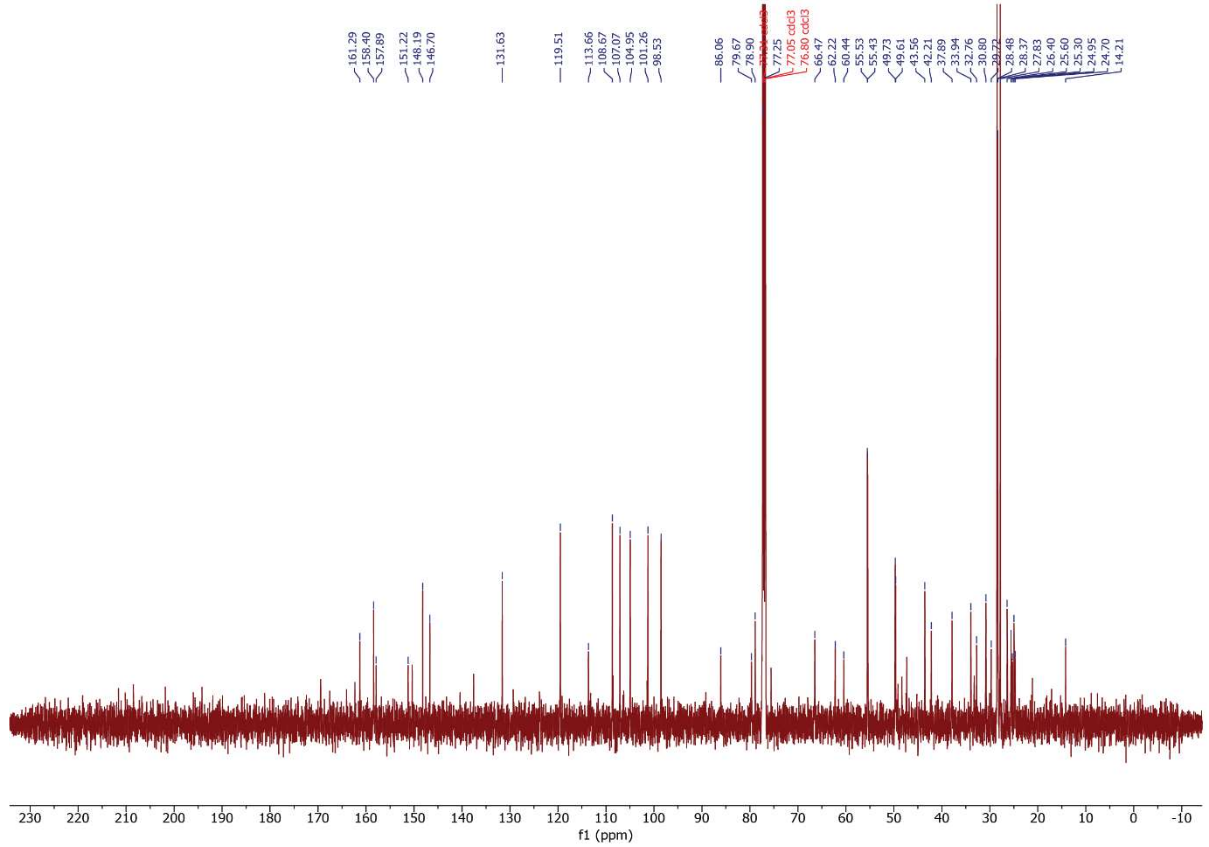
161.29
158.40
157.89
151.22
148.19
146.70
131.63
119.51
113.66
108.67
107.07
104.95
101.26
98.53
86.06
79.67
78.90
77.30 cdcl3
77.25
77.05 cdcl3
76.80 cdcl3
66.47
62.22
60.44
55.53
55.43
49.73
49.61
43.56
42.21
37.89
33.94
32.76
30.80
29.72
28.48
28.37
27.83
26.40
25.60
25.30
24.95
24.70
14.21
230
220
210
200
190
180
170
160
150
140
130
120
110
100
90
80
70
60
50
40
30
20
10
0
-10
f1 (ppm)

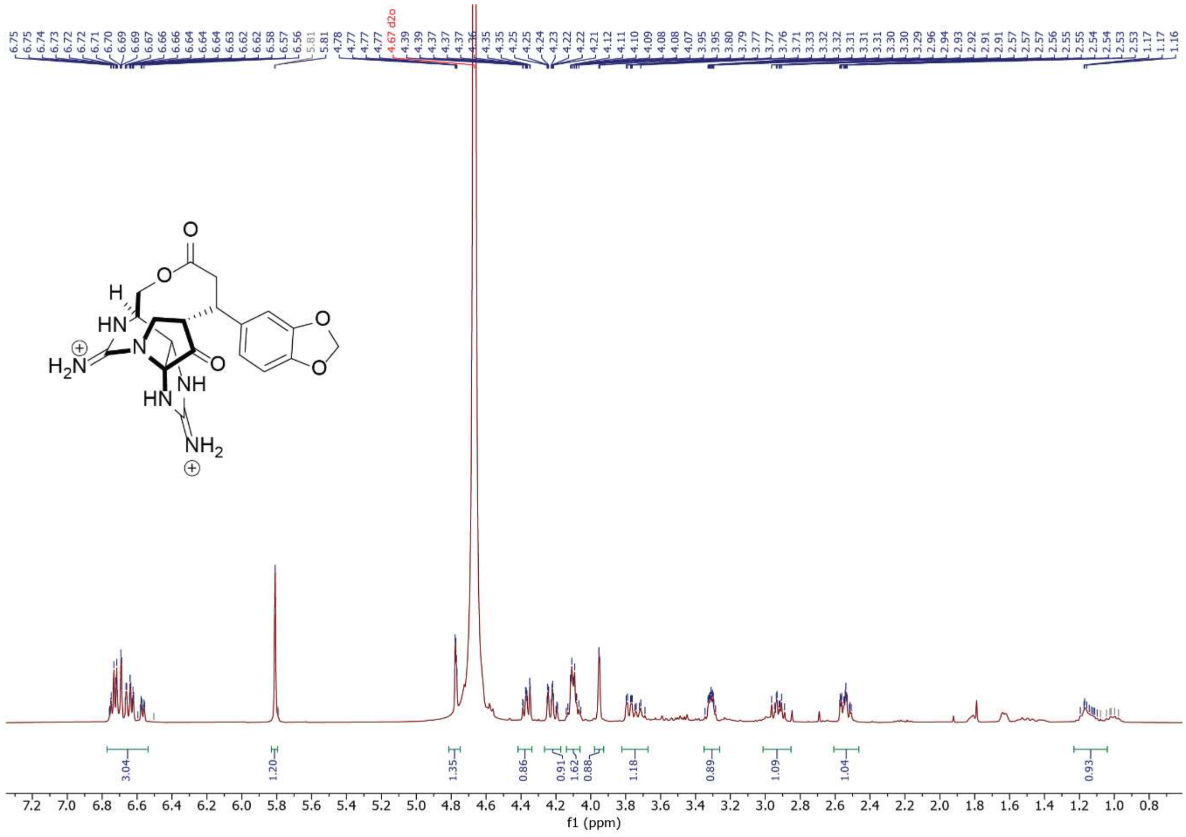
6.75 6.75 6.74 6.73 6.72 6.72 6.71 6.70 6.69 6.69 6.67 6.66 6.66 6.64 6.64 6.63 6.62 6.62 6.58 6.57 6.56 5.81 5.81 4.78 4.77 4.77 4.77 4.67 d2o 4.39 4.39 4.37 4.37 4.37 4.36 4.35 4.35 4.25 4.25 4.24 4.23 4.22 4.22 4.21 4.12 4.11 4.10 4.09 4.08 4.08 4.07 3.95 3.95 3.80 3.79 3.77 3.77 3.76 3.71 3.33 3.32 3.32 3.31 3.31 3.31 3.30 3.30 3.29 2.96 2.94 2.93 2.92 2.91 2.91 2.57 2.57 2.57 2.56 2.55 2.55 2.54 2.54 2.53 1.17 1.16
3.04 1.20 1.35 0.86 0.91 1.62 0.88 1.18 0.89 1.09 1.04 0.93
f1 (ppm)

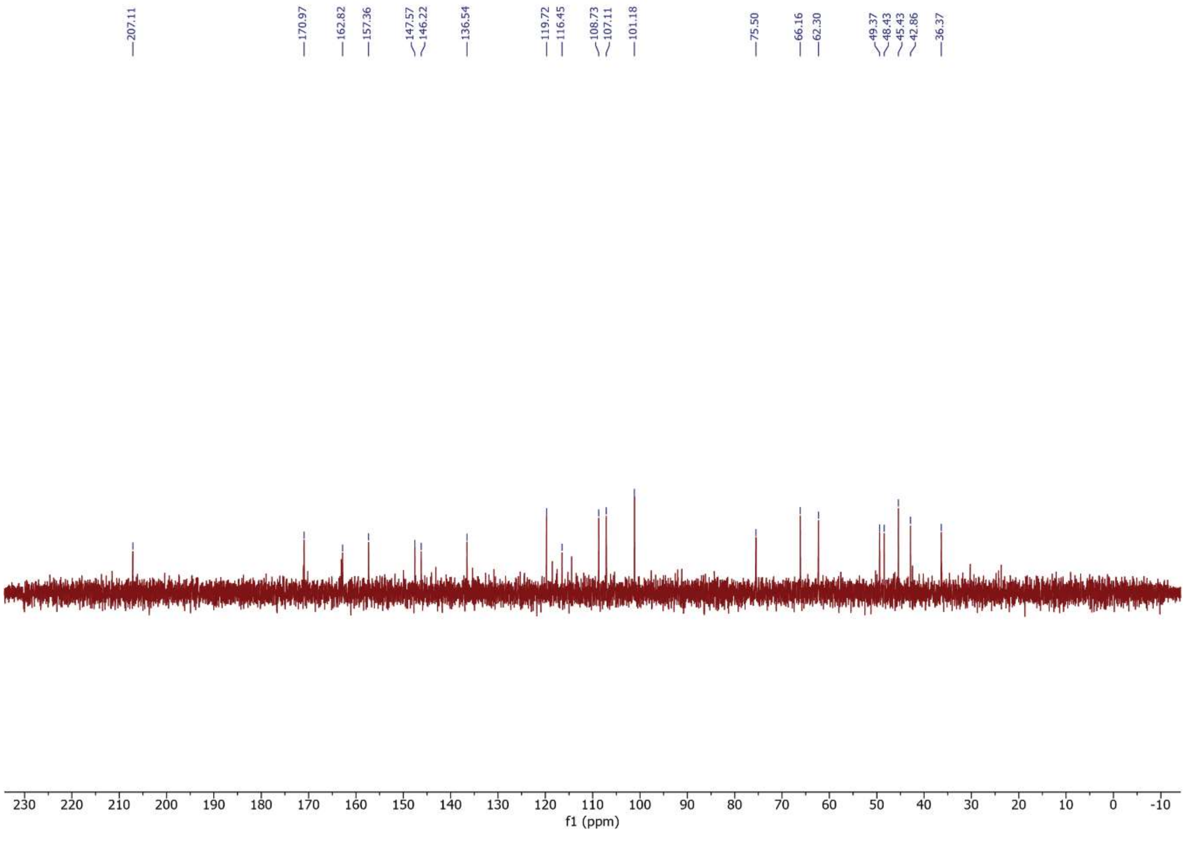
207.11 170.97 162.82 157.36 147.57 146.22 136.54 119.72 116.45 108.73 107.11 101.18 75.50 66.16 62.30 49.37 48.43 45.43 42.86 36.37
f1 (ppm)

1. (1) Teichert, R. W.; Schmidt, E. W.; Olivera, B. M. Constellation pharmacology: a new paradigm for drug discovery. Annu Rev Pharmacol Toxicol 2015, 55, 573-589. DOI: 10.1146/annurev-pharmtox-010814-124551.
2. (2) Teichert, R. W.; Smith, N. J.; Raghuraman, S.; Yoshikami, D.; Light, A. R.; Olivera, B. M. Functional profiling of neurons through cellular neuropharmacology. Proc Natl Acad Sci U S A 2012, 109 (5), 1388-1395. DOI: 10.1073/pnas.1118833109.
3. Lu, C.; Wu, C.; Ghoreishi, D.; Chen, W.; Wang, L.; Damm, W.; Ross, G. A.; Dahlgren, M. K.; Russell, E.; Von Bargen, C. D.; Abel, R.; Friesner, R. A.; Harder, E. D. *J. Chem. Theory Comput*. **2021**, 17, 4291–4300.
4. Gaussian 16, Revision C.01, Frisch, M. J.; Trucks, G. W.; Schlegel, H. B.; Scuseria, G. E.; Robb, M. A.; Cheeseman, J. R.; Scalmani, G.; Barone, V.; Petersson, G. A.; Nakatsuji, H.; Li, X.; Caricato, M.; Marenich, A. V.; Bloino, J.; Janesko, B. G.; Gomperts, R.; Mennucci, B.; Hratchian, H. P.; Ortiz, J. V.; Izmaylov, A. F.; Sonnenberg, J. L.; Williams-Young, D.; Ding, F.; Lipparini, F.; Egidi, F.; Goings, J.; Peng, B.; Petrone, A.; Henderson, T.; Ranasinghe, D.; Zakrzewski, V. G.; Gao, J.; Rega, N.; Zheng, G.; Liang, W.; Hada, M.; Ehara, M.; Toyota, K.; Fukuda, R.; Hasegawa, J.; Ishida, M.; Nakajima, T.; Honda, Y.; Kitao, O.; Nakai, H.; Vreven, T.; Throssell, K.; Montgomery, J. A., Jr.; Peralta, J. E.; Ogliaro, F.; Bearpark, M. J.; Heyd, J. J.; Brothers, E. N.; Kudin, K. N.; Staroverov, V. N.; Keith, T. A.; Kobayashi, R.; Normand, J.; Raghavachari, K.; Rendell, A. P.; Burant, J. C.; Iyengar, S. S.; Tomasi, J.; Cossi, M.; Millam, J. M.; Klene, M.; Adamo, C.; Cammi, R.; Ochterski, J. W.; Martin, R. L.; Morokuma, K.; Farkas, O.; Foresman, J. B.; Fox, D. J. Gaussian, Inc., Wallingford CT, **2016**.
5. Goerigk, L.; Grimme, S. *Phys. Chem. Chem. Phys*. **2011**, 13, 6670–6688.
6. Luchini, G. Alegre-Requena, J. V.; Funes-Adroiz, I.; Paton, R. S. *F1000Research*. **2020**, 9, 291.
7. NBO Version 3.1, E. D. Glendening, A. E. Reed, J. E. Carpenter, and F. Weinhold.
8. Breneman, C. M.; Wiberg, K. B. *J. Comput. Chem*. **1990**, 11, 361–373.
9. Hirshfeld, F. L. *Theor. Chim. Acta*. **1977**, 44, 129–138.
10. Lu, T.; Chen, F. *J. Theor. Comput. Chem*. **2012**, 11, 163–183.
11. Marenich, A. V.; Jerome, S. V.; Cramer, C. J.; Truhlar, D. G. *J. Chem. Theory Comput*. **2012**, 8, 527–541.
12. Wolinski, K.; Hinton, J. F.; Pulay, P. *J. Am. Chem. Soc*. **1990**, 112, 8251–8260.
13. Hilal, S. H.; Bornander, L. L.; Carreira, L. A. *QSAR Comb. Sci*. **2005**, *24*, 631–638.
14. Berge, A. H.; Pugh, S. M.; Short, M. I. M.; Kaur, C.; Lu, Z.; Lee, J-H.; Pickard, C. J.; Sayari, A.; Forse, A. C. *Nat. Comm*. **2022**, *13*, 7763.
